# Phosphonate and Thiasugar Analogues of Glucosamine-6-phosphate: Activation of the *glmS* Riboswitch and Antibiotic Activity

**DOI:** 10.1021/acschembio.3c00452

**Published:** 2023-10-04

**Authors:** Bjarne Silkenath, Dennis Kläge, Hanna Altwein, Nina Schmidhäuser, Günter Mayer, Jörg S. Hartig, Valentin Wittmann

**Affiliations:** † Department of Chemistry, University of Konstanz, 78457 Konstanz, Germany; ‡ LIMES Institute, Center for Aptamer Research & Development, University of Bonn, 53121 Bonn, Germany

## Abstract

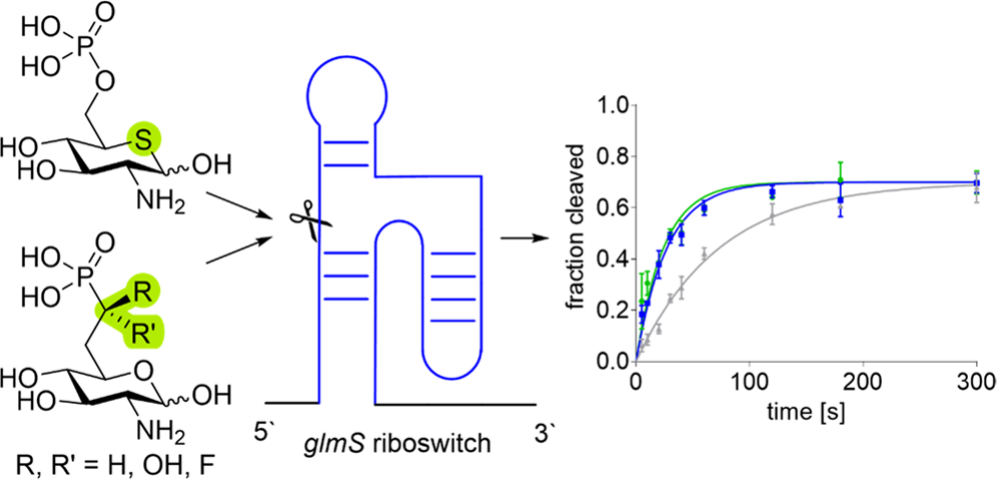

The *glmS* riboswitch is a motif found in 5′-untranslated regions of bacterial mRNA that controls the synthesis of glucosamine-6-phosphate (GlcN6P), an essential building block for the bacterial cell wall, by a feedback mechanism. Activation of the *glmS* riboswitch by GlcN6P mimics interferes with the ability of bacteria to synthesize its cell wall. Accordingly, GlcN6P mimics acting as *glmS* activators are promising candidates for future antibiotic drugs that may overcome emerging bacterial resistance against established antibiotics. We describe the synthesis of a series of phosphonate mimics of GlcN6P as well as the thiasugar analogue of GlcN6P. The phosphonate mimics differ in their p*K*_a_ value to answer the question of whether derivatives with a p*K*_a_ matching that of GlcN6P would be efficient *glmS* activators. We found that all derivatives activate the riboswitch, however, less efficiently than GlcN6P. This observation can be explained by the missing hydrogen bonds in the case of phosphonates and is valuable information for the design of future GlcN6P mimics. The thiasugar analogue of GlcN6P on the other hand turned out to be a *glmS* riboswitch activator with the same activity as the natural metabolite GlcN6P. The nonphosphorylated thiasugar displayed antimicrobial activity against certain bacilli. Therefore, the compound is a promising lead structure for the development of future antibiotics with a potentially novel mode of action.

## Introduction

**Figure 1:**
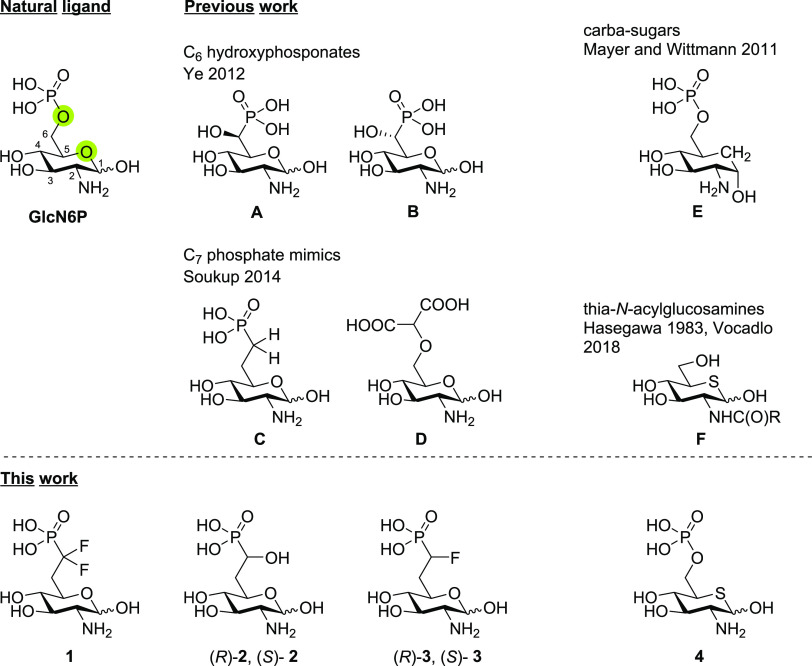
Natural ligand of the glmS ribozyme, GlcN6P, and mimics thereof. C-6 Hydroxyphosphonates **A** and **B** synthesized by Ye^21^ and C-7 methylene phosphonate **C** and carboxylate phosphate surrogate **D** synthesized by Soukup.^22^ Mayer and Wittmann yielded carbasugar analogue **E** of GlcN6P by replacement of the ring oxygen with a carbon atom.^23^ Thia-*N*-acylglucosamines **F** synthesized by Hasegawa^24^ and Vocadlo.^25,26^ Promising glmS riboswitch activators **1**–**4** synthesized and investigated in this work.

The development of multiple drug-resistant bacteria has taken an alarming speed and could yield a public health crisis on the scale of the recent COVID-19 pandemic or even worse if it remains unchecked.^1^ Therefore, the development of new antibiotics in the fight against drug-resistant bacteria, especially the development of antibiotics acting on unexploited targets in the bacterial metabolism, is of utmost importance.^2,3^ The *glmS* riboswitch is one of these potential targets.^4−10^ It is found in the 5′-untranslated regions of bacterial mRNA^9,11−15^ and controls the gene encoding for the enzyme glucosamine-6-phosphate synthase (GlmS).^4,16−18^ This enzyme catalyzes the synthesis of glucosamine-6-phosphate (GlcN6P, Figure 1) from glutamine and fructose-6-phosphate (Fru6P). GlcN6P can bind to the *glmS* riboswitch, catalyzing the self-cleavage of this RNA construct, which in return leads to the degradation of the downstream coding RNA by RNase J1.^19^ GlcN6P is essential for the cell wall synthesis of bacteria. Activation of the *glmS* riboswitch by a drug is desirable to interrupt the ability of bacteria to synthesize GlcN6P. A GlcN6P mimic acting as an *glmS* activator and therefore interfering with the bacterial ability to synthesize its cell wall represents a promising candidate for a future antibiotic drug.^5,20^

The structural requirements for GlcN6P mimics to catalyze the self-cleavage reaction of the *glmS* riboswitch have been thoroughly investigated in the past.^4,12,22,27−31^ Removal of the hydroxy group at the anomeric position, that has been shown to be recognized only in the α-orientation, is associated with a significant loss of activity.^27,32^ The 2-amino group of GlcN6P has been shown to be essential for an efficient cleavage.^28,32^ Inversion of the stereochemistry of the 3-hydroxy group results in a decrease of the self-cleavage rate constant *k*_obs_ by a factor of 3.5.^27^ Removal or inversion of the stereochemistry of the 4-hydroxy group leads to a total loss of activation.^22,28,32^ However, a position that can be potentially varied is the phosphate group at OH-6. Phosphate mimics have found widespread application in drug design^33−35^ and offer access to phosphatase-resistant GlcN6P mimetics. Indeed, this approach has been pursued previously. The Ye group synthesized the C_6_ hydroxyphosphonates **A** and **B** (Figure 1) that suffer from what the authors describe as massive loss of activation.^21^ The reduced activity might be explained by the shorter connection between the sugar ring and the phosphor atom (2 bonds) when compared to GlcN6P (3 bonds). The authors also argue that the different electrical properties of hydroxyphosphonates and phosphates might be the cause of the observed loss of activity.^21^ The Soukup group addressed the steric issue by synthesizing C_7_ methylene phosphonate **C** as well as malonic acid derivative **D** and a phosphoramidate (not shown) as phosphate surrogates.^36^ However, also, these compounds showed a strongly reduced initiation of the self-cleavage reaction being approximately one-seventh of that of the natural ligand GlcN6P.^36^ The authors argue that the reduced activity might be explained by the lower acidity of phosphonate **C** (p*K*_a2_ = 7.4) in comparison to GlcN6P (p*K*_a2_ = 6.2), which might result in a different ability to bind Mg^2+^ ions. Magnesium chelation has been suggested to be required for successful cleavage of the riboswitch.^37^ A systematic investigation of phosphonate analogues of GlcN6P with varying p*K*_a_ values and their ability to induce self-cleavage of the *glmS* riboswitch, however, has not been carried out.

A second position that can be varied is the ring oxygen being part of the hemiacetal of GlcN6P. Carba-sugar **E**, a GlcN6P derivative in which the ring oxygen is replaced with a methylene group, has proven to be an effective activator of the *glmS* riboswitch.^5,23^ Carba-GlcN6P derivatives with substituents in the carba position have also been synthesized.^38^ We hypothesized that the ring oxygen can also be replaced with a sulfur atom, while retaining the ability to initiate the self-cleavage reaction. Thia-*N*-acetylglucosamine **F** (R = Me) has been synthesized by Hasegawa^24^ and used by Vocadlo as an glycosyltransferase inhibitor in mammalian cells.^25,26^ Thia-glucosamine-6-phosphate **4** (thia-GlcN6P) has been proposed as a likely intermediate in the metabolism of thia-*N*-acylglucosamines **F** in mammals;^25^ however, it has not been synthesized and investigated up to now.

Here, we report the synthesis of two classes of GlcN6P mimics that explore the two possible sites of modification discussed above. The C_7_ phosphonate derivatives with difluoro (**1**), hydroxy ((*R*)-**2** and (*S*)-**2**), and monofluoro substitution ((*R*)-**3** and (*S*)-**3**) have the same length of the side chain as the natural *glmS* riboswitch activator GlcN6P and differ in their acidity. Fluorophosphonates have been reported to be very similar to the corresponding phosphates regarding their steric and electronic properties.^33,39,40^ Accordingly, the monofluorophosphonates (*R*)-**3** and (*S*)-**3** were expected to have a similar acidity as GlcN6P. Difluorophosphonate **1** on the other hand was expected to be more acidic, and the hydroxyphosphonates (*R*)-**2** and (*S*)-**2** were expected to be less acidic than GlcN6P. Furthermore, we report the synthesis of thia-GlcN6P **4** that explores the exchange of the ring oxygen with a sulfur atom. All compounds have been tested for their ability to activate the *glmS* riboswitch and induce self-cleavage. While the phosphonates, regardless of their acidity, turned out to be less efficient riboswitch activators, thia-GlcN6P **4** activated self-cleavage of the *glmS* riboswitch with the same efficiency as the natural metabolite GlcN6P. A detailed look at the published X-ray structure of the *glmS* riboswitch in complex with GlcN6P provided an explanation of the reduced activity of the phosphonates and allowed us to draw conclusions for the design of future riboswitch activators. In addition, we investigated the antimicrobial properties of the synthesized compounds and found thia-GlcN, the biochemical precursor of thia-GlcN6P **4**, to inhibit the growth of *Bacillus subtilis* and *Bacillus thuringiensis*.

## Results and Discussion

### Synthesis of α,α-Difluorophosphonate **1**

**Scheme 1:**
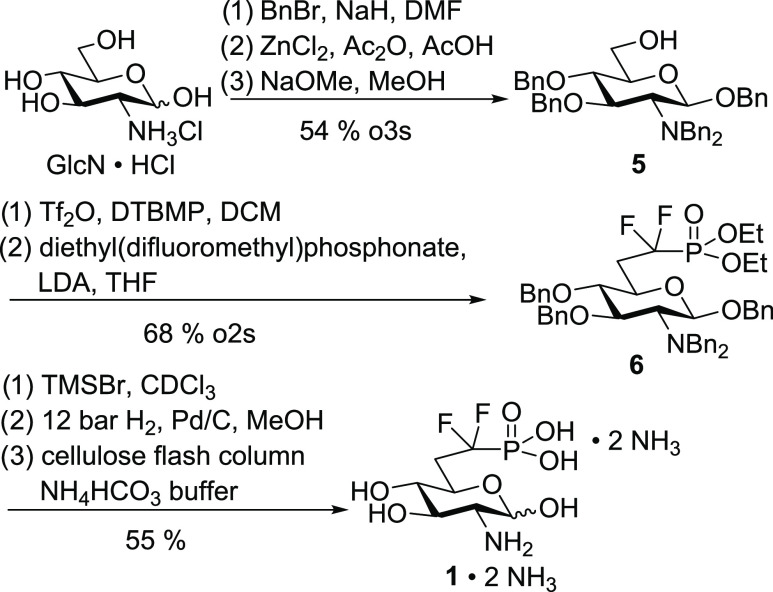
Synthesis of Difluorophosphonate **1**·2 NH_3_

To introduce phosphate mimics in the 6-position, the amino group in the 2-position and all hydroxy groups of glucosamine except for the primary one need to be protected. To achieve this, glucosamine hydrochloride was perbenzylated followed by acetolysis using zinc chloride in acetic anhydride and acetic acid to convert the benzyl ether in position 6 into an acetate (Scheme 1). De-*O*-acetylation with sodium methoxide gave primary alcohol **5** in a yield of 54% over three steps besides small amounts of the α-anomer. The synthesis of **5** was previously reported by Ye carrying out acetolysis with sulfuric acid.^21^ However, we opted for the use of zinc chloride^41^ because these conditions gave higher and more consistent yields in our hands. Alcohol **5** was activated with triflic anhydride and 2,6-di-*tert*-butyl-4-methylpyridine (DTBMP), and the obtained triflate was directly converted to difluorophosphonate **6** by reaction with diethyl(difluoromethyl)phosphonate and LDA in a yield of 68% over two steps. After deprotection of the phosphonate with trimethylsilyl bromide (TMSBr) in CDCl_3_, to facilitate reaction monitoring by NMR, the benzyl groups were cleaved off by hydrogenation at 12 atm of H_2_ under palladium catalysis. The obtained difluorophosphonate was purified by cellulose flash column chromatography using ammonium bicarbonate buffer as an eluent to give the diammonium salt **1**·2 NH_3_ in a yield of 55%.

### Synthesis of α-Hydroxyphosphonates (*R*)-**2** and (*S*)-**2**

**Scheme 2:**
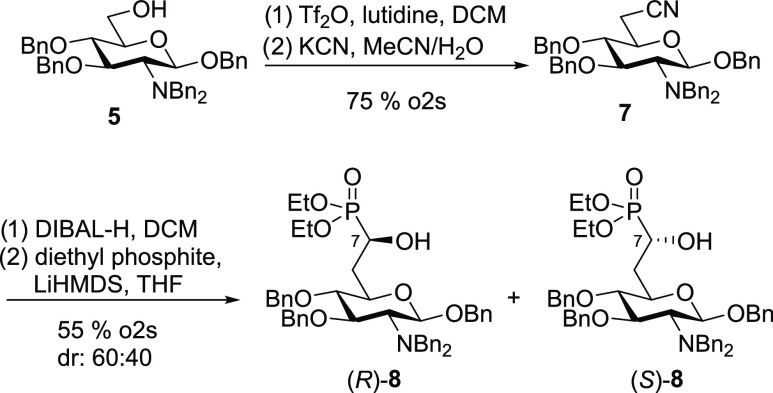
Synthesis of Hydroxyphosphonates (*R*)-**8** and (*S*)-**8** by C_1_ Elongation of **5**

The primary alcohol **5** was converted to the corresponding triflate as described before and then treated with potassium cyanide to give nitrile **7** in a yield of 75% over two steps (Scheme 2). Reduction of the nitrile with diisobutylaluminum hydride (DIBAL-H) to the corresponding aldehyde followed by an attack of diethyl phosphite using lithium bis(trimethylsilyl) amide (LiHMDS) as a base gave a separable mixture of the two diastereomers (*R*)-**8** and (*S*)-**8** in a ratio of 60:40 and a combined yield of 55%.

**Figure 2:**
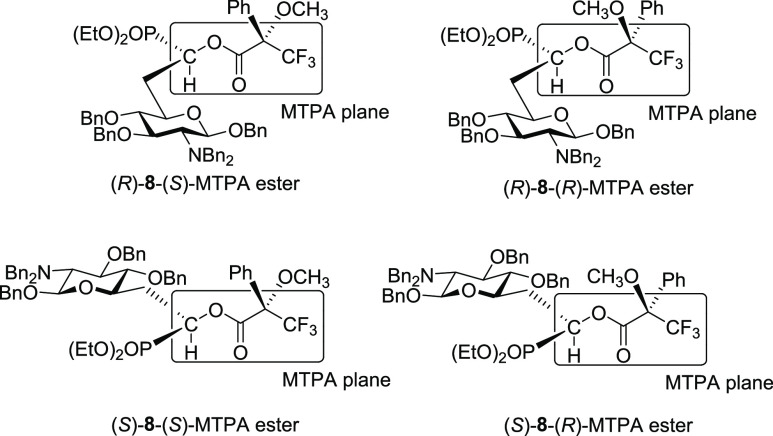
Synthesized Mosher esters of (*R*)-**8** and (*S*)-**8** that were used for the determination of the stereochemistry at C-7. According to Mosher rules, signals of nuclei on the same side of the MTPA plane as the Ph group are upfield-shifted relative to the signals of the isomer, in which these nuclei are on the opposite side of the MTPA plane as the Ph group. MTPA = methoxy-α-(trifluoromethyl)phenylacetic acid.

To determine the absolute configuration of the newly formed stereocenter at C-7, hydroxyphosphonates **8** were converted to the corresponding Mosher esters. Both for the major and minor isomers, we prepared the (*S*)- and the (*R*)-MTPA ester (Figure 2). After assignment of all proton signals in the ^1^H NMR spectra, we determined the chemical shift differences Δδ^SR^ = δ^S^ – δ^R^ of all signals for the (*S*)- and (*R*)-MTPA ester (Table S1).^42,43^ For the major isomer, all Δδ^SR^ values of the sugar resonances were negative and all Δδ^SR^ values of the phosphonate resonances (ethyl groups as well as ^31^P resonances) were positive. Accordingly, the major isomer was assigned to be (*R*)-**8**. Similarly, for the minor isomer, all Δδ^SR^ values had opposite signs, and this isomer was assigned to be (*S*)-**8**.

**Scheme 3:**
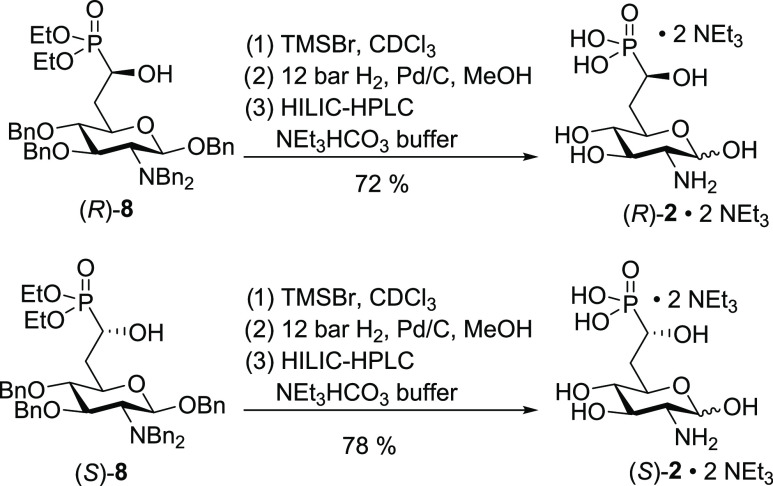
Deprotection of (*R*)-**8** and (*S*)-**8**

The final deprotection of (*R*)-**8** and (*S*)-**8** was achieved in each of the two steps (Scheme 3). Cleavage of the ethyl groups was achieved with TMSBr. Subsequent hydrogenation at 12 atm H_2_ with palladium on carbon affected benzyl deprotection. After purification by HILIC HPLC, the two diastereomers were obtained as bis(triethylammonium) salts (*R*)-**2**·2 NEt_3_ and (*S*)-**2**·2 NEt_3_ in a yield of 72 and 78%, respectively.

### Synthesis of α-Fluorophosphonates (*R*)-**3** and (*S*)-**3**

**Scheme 4:**
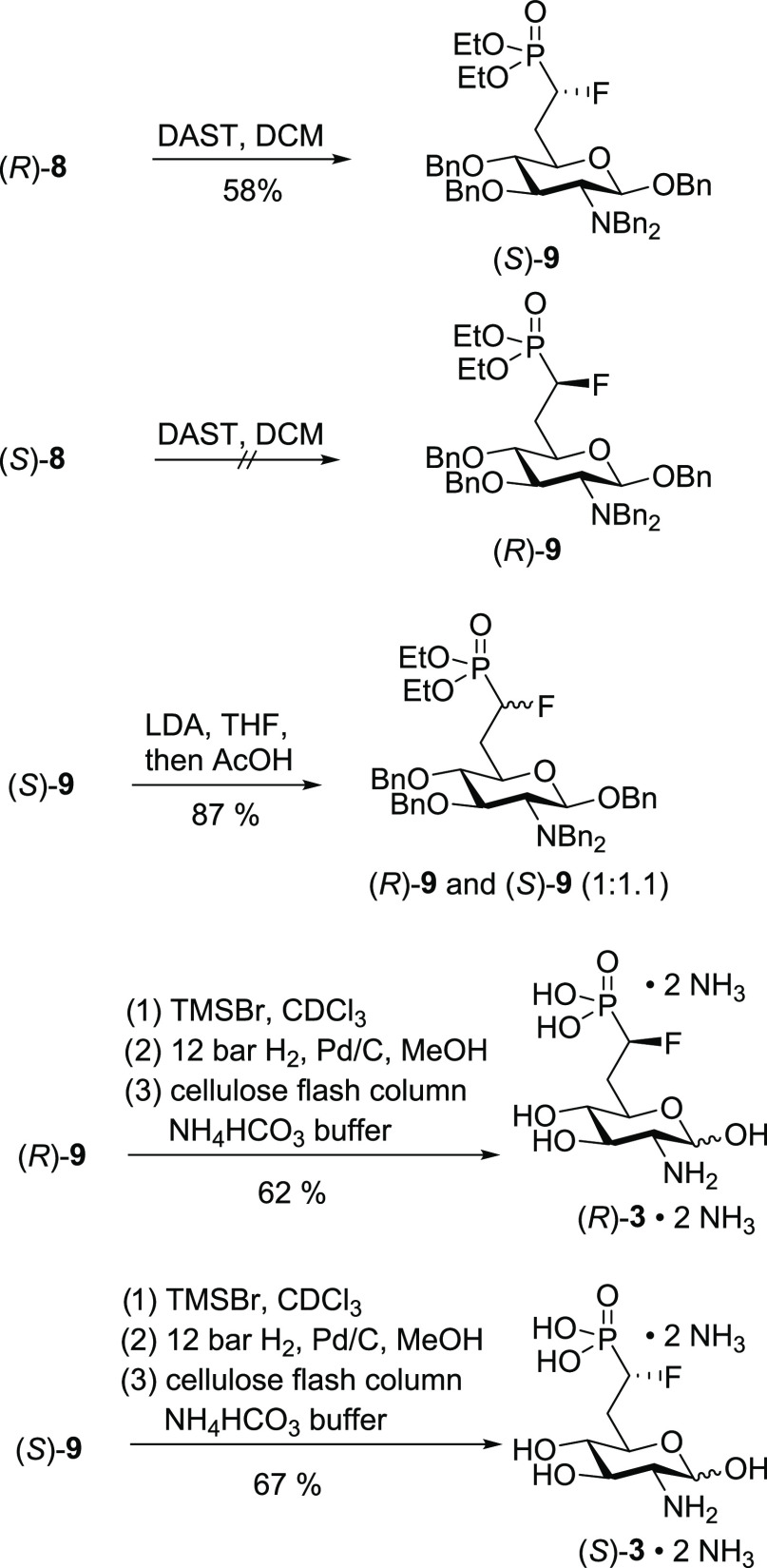
Synthesis of Fluorophosphonates (*R*)-**3** and (*S*)-**3**

Since the electronic and steric properties of fluorophosphonates have been reported to be very similar to those of phosphates,^39,40^ we deemed them promising modifications of GlcN6P. Monofluorophosphonates are accessible from the corresponding hydroxyphosphonates by deoxyfluorination.^33^ A typical reagent to substitute a hydroxy group with a fluoride under inversion of configuration is diethylaminosulfur trifluoride (DAST).^44^ When we treated the diastereomeric hydroxyphosphonates (*R*)-**8** and (*S*)-**8** with DAST, we observed that only (*R*)-**8** reacted with DAST to the corresponding fluoride, while (*S*)-**8** decomposed during the reaction (Scheme 4). Earlier, the Berkowitz group reported the synthesis of fluorophosphonate analogues of glucose 6-phosphate as substrate mimics for glucose 6-phosphate dehydrogenase. Interestingly, when they reacted the glucose analogues of (*R*)-**8** and (*S*)-**8** (OBn instead of NBn_2_ in position 2) with DAST, only the (*R*) diastereomer reacted smoothly to the (*S*)-configured fluoride under inversion of the configuration, whereas the (*S*)-configured hydroxyphosphonate decomposed during the reaction.^33^ Given the similarity of the two isomers of **8** to the hydroxyphosphonates investigated by Berkowitz, we assume that also in the case of (*R*)-**8**, an inversion of configuration takes place. Accordingly, the reaction product obtained from (*R*)-**8** in a yield of 58% is expected to be fluorophosphonate (*S*)-**9**.

We also investigated a large variety of alternative deoxyfluorination reagents to achieve the conversion of (*S*)-**8** to (*R*)-**9** including PyFluor,^45^ pentafluorobenzenesulfonyl fluoride,^45^ Deoxo-Fluor,^46^ Xtal-Fluor-M,^47^ and Xtal-Fluor-E.^47^ However, in all cases, either no reaction or a decomposition similar to the reaction with DAST occurred. To gain access to compound (*R*)-**9**, we performed an isomerization of (*S*)-**9** by treatment with LDA giving a 1:1.1 mixture of (*R*)-**9** and (*S*)-**9** upon workup using acetic acid that could be separated by flash chromatography. Deprotection of (*R*)-**9** and (*S*)-**9** was achieved as described above for (*R*)-**8** and (*S*)-**8** and gave the diastereomeric fluorophosphonates after purification by cellulose flash column chromatography using ammonium bicarbonate buffer as diammonium salts (*R*)-**3**·2 NH_3_ and (*S*)-**3**·2 NH_3_ in a yield of 62 and 67%, respectively.

### Determination of p*K*_a_ Values

**Figure 3:**
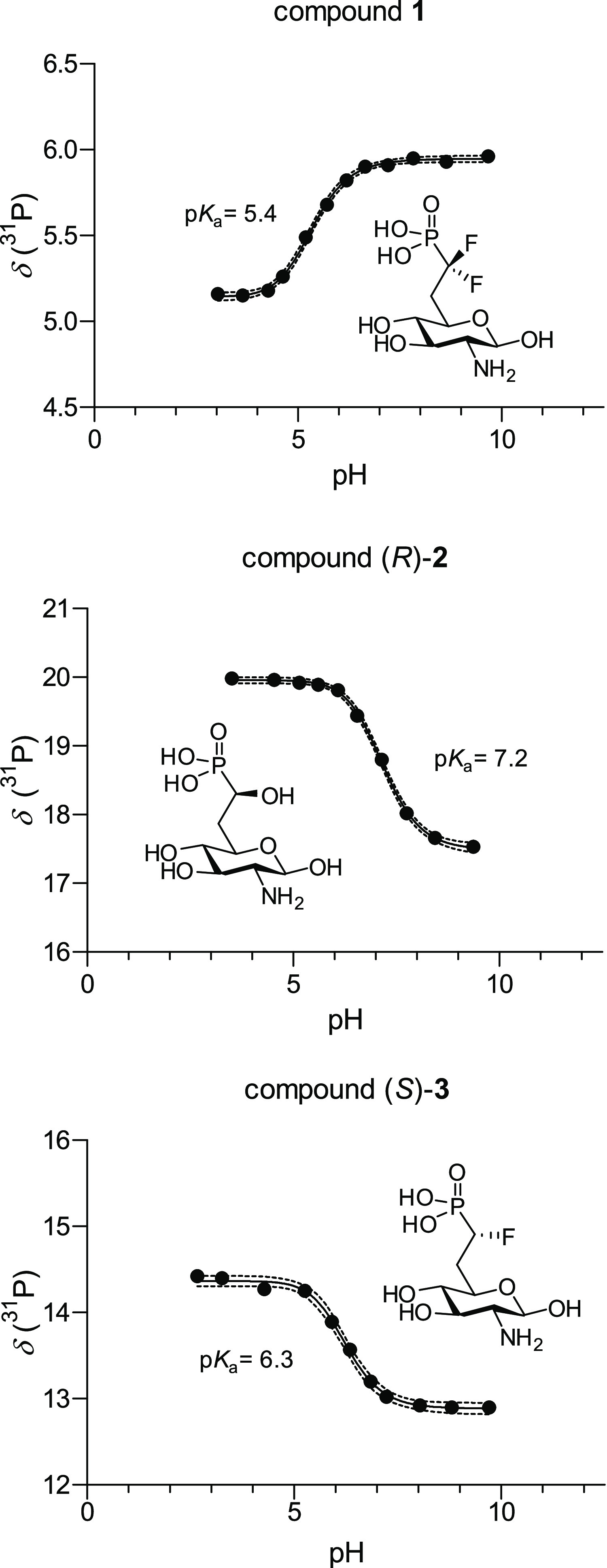
^31^P NMR titration curves of **1**, (*R*)-**2**, and (*S*)-**3**. Fitted sigmoidal function and 95% confidence interval.

The mechanism of the GlcN6P-induced self-cleavage of the *glmS* ribozyme involves the coordination of hydrated magnesium ions by the phosphate group existing in the dianion form. To estimate the ability of the newly synthesized phosphonates to coordinate magnesium, we determined their p*K*_a2_ values as a measure for the amount of dianions present at physiological pH. Since potentiometric acid–base titrations have the disadvantage that it is difficult or even impossible to distinguish the p*K*_a_ values of multiple functional groups within a molecule, especially when they are similar as in the case of modified phosphonates and the amine of glucosamine, we recorded ^31^P NMR spectra at a series of pH values and plotted the ^31^P shifts against the pH value (for details, see the Supporting Information). This allowed fitting of a sigmoidal function the point of inflection of which represents the p*K*_a_ value.^48^ Two literature-known compounds were also investigated and used as a control (Figure S1). The p*K*_a2_ value of GlcN6P was determined to be 6.2 ± 0.1, which is in accordance with the literature value of 6.2 reported by Soukup.^22^ Similarly, the p*K*_a2_ value of methylene phosphonate **C** was determined to be 7.5 ± 0.1, which is in accordance to the value of 7.4 reported by Soukup.^36^ Having shown the accuracy of the NMR-based p*K*_a_ determination, we investigated difluorophosphonate **1**, hydroxyphosphonate (*R*)-**2**, and monofluorophosphonate (*S*)-**3** (Figure 3). The p*K*_a2_ value of **1** was determined to be 5.4 ± 0.1, the p*K*_a2_ value of (*R*)-**2** was determined to be 7.2 ± 0.1, and the p*K*_a2_ value of (*S*)-**3** was determined to be 6.3 ± 0.1. We assumed that the stereochemistry at the α position of the phosphonate has no influence on the p*K*_a_ value. As anticipated, the synthesized phosphate mimics span a wide range of p*K*_a2_ values with difluorophosphonate **1** being a mimic that is more acidic than GlcN6P and hydroxyphosphonates **2** (similarly as literature-known **C**) being less acidic. The p*K*_a_ value of monofluorophosphonates **3** on the other hand nearly perfectly matches the one of the natural ligand GlcN6P. Thus, the synthesized library of phosphonates was well suited to study the effect of the p*K*_a2_ value of the phosphonate derivatives on activation of the *glmS* riboswitch (vide infra).

### Synthesis of Thia-glucosamine-6-phosphate **4**

**Scheme 5:**
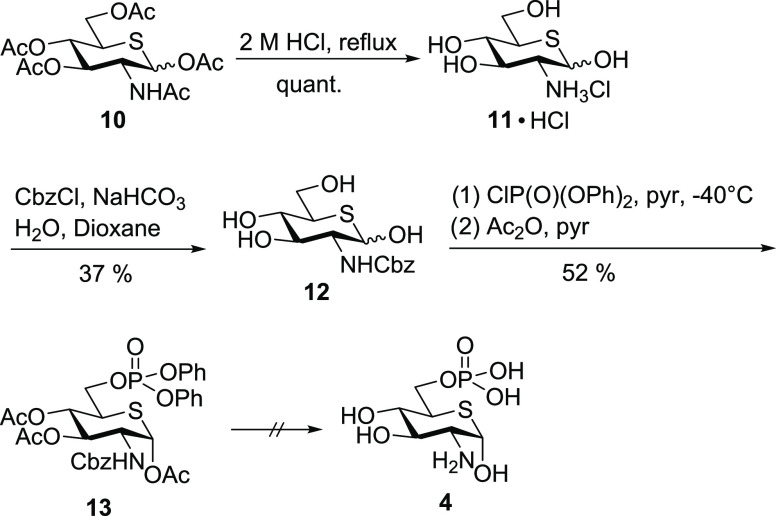
Initial Attempt to Synthesize Thia-GlcN6P **4**

Scheme 5 depicts the initial attempt to synthesize thia-GlcN6P **4**. Protected thia-*N*-acetylglucosamine derivative **10** was synthesized from GlcNAc in nine steps and a total yield of 31% following the procedure published by Hasegawa.^24^*O*- and *N*-Deacetylation was achieved quantitatively with 2 M HCl as reported by Vocadlo^25,26^ to give thia-glucosamine hydrochloride **11**·HCl. In a first approach toward thia-GlcN6P **4**, the 2-amino group was Cbz-protected to give **12** followed by the introduction of the phosphate with diphenyl chlorophosphate and subsequent acetylation to give **13**. The final deprotection steps included a hydrogenation with PtO_2_ catalyst to liberate the phosphate, followed by hydrogenation with Pd–C as a catalyst to remove the Cbz group. We chose this strategy because it proved successful in our previous synthesis of carbasugar analogues of glucosamine-6-phosphate.^38^ However, these conditions were not applicable to **13** presumably due to catalyst poisoning by the hemithioacetal.

**Scheme 6:**
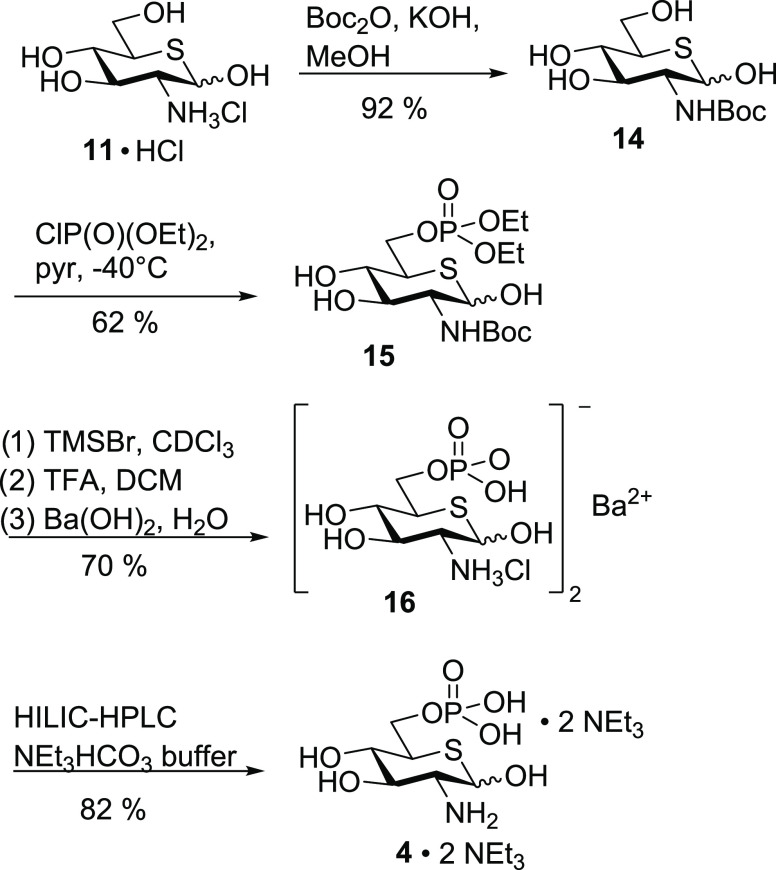
Synthesis of Bis(triethylammonium) Thia-GlcN6P **4**·2 NEt_3_

We therefore changed the protecting group strategy and employed Boc protection of the amine and ethyl ester protection of the phosphate. Thiasugar **11**·HCl was treated with Boc anhydride in the presence of KOH, yielding protected amine **14** in a yield of 92% (Scheme 6). Regioselective phosphorylation of the 6-position was achieved using diethyl chlorophosphate in pyridine to give thiasugar phosphate **15** in a yield of 62%. Following this strategy, the total deprotection could be carried out without the use of a metal catalyst and was easily achieved by treatment with TMSBr to cleave the diethyl phosphate, followed by addition of trifluoroacetic acid to achieve Boc deprotection. The crude phosphate was precipitated as the barium salt **16**, which was obtained in a yield of 70% over three steps. This salt was purified by HILIC HPLC using triethylammonium bicarbonate buffer as an eluent to give the pure bis(triethylammonium) salt **4**·2 NEt_3_.

### Activation of *glmS* Ribozyme Self-Cleavage

**Figure 4:**
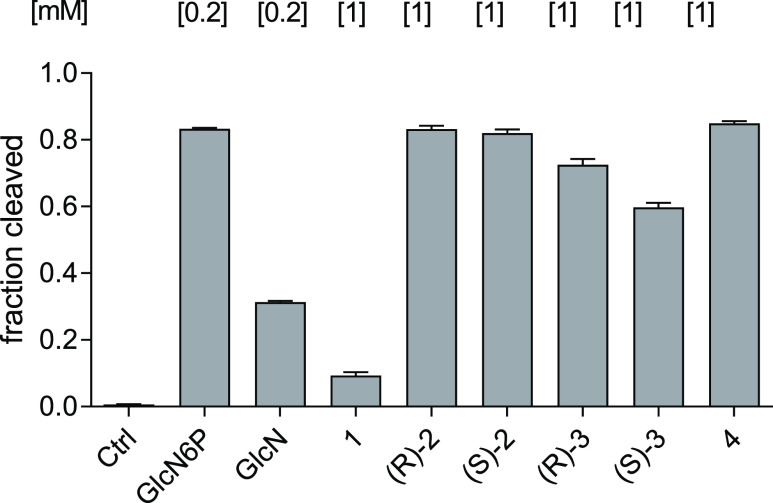
Initial assessment of the activity to activate self-cleavage of the *glmS* ribozyme from *B. subtilis* by the newly synthesized compounds **1**, (*R*)-**2**, (*S*)-**2**, (*R*)-**3**, (*S*)-**3**, and **4**. As a control, the RNA was incubated in the presence of 10 mM Mg^2+^ without any compound (Ctrl). GlcN6P and GlcN were used for comparison at a concentration of 0.2 mM. All new compounds were tested at 1 mM concentration and incubated for 30 min. The error bars display the standard deviation of triplicates.

To investigate the ability of the newly synthesized compounds to induce catalytic activity of the *glmS* ribozyme resulting in its self-cleavage, we performed ligand-dependent self-cleavage assays^23^ with a 5′-^32^P labeled ribozyme sequence from *B. subtilis*. For an initial activity assessment, compounds **1**, (*R*)-**2**, (*S*)-**2**, (*R*)-**3**, (*S*)-**3**, or **4** were incubated with the *glmS* ribozyme in the presence of 10 mM MgCl_2_. These experiments revealed that compounds (*R*)-**2**, (*S*)-**2**, and **4** resulted in efficient cleavage of the ribozyme at a concentration of 1 mM (Figure 4). Compounds (*R*)-**3** and (*S*)-**3** showed slightly diminished activity, and difluorophosphonate **1** caused only a minor induction of the self-cleavage reaction.

**Figure 5:**
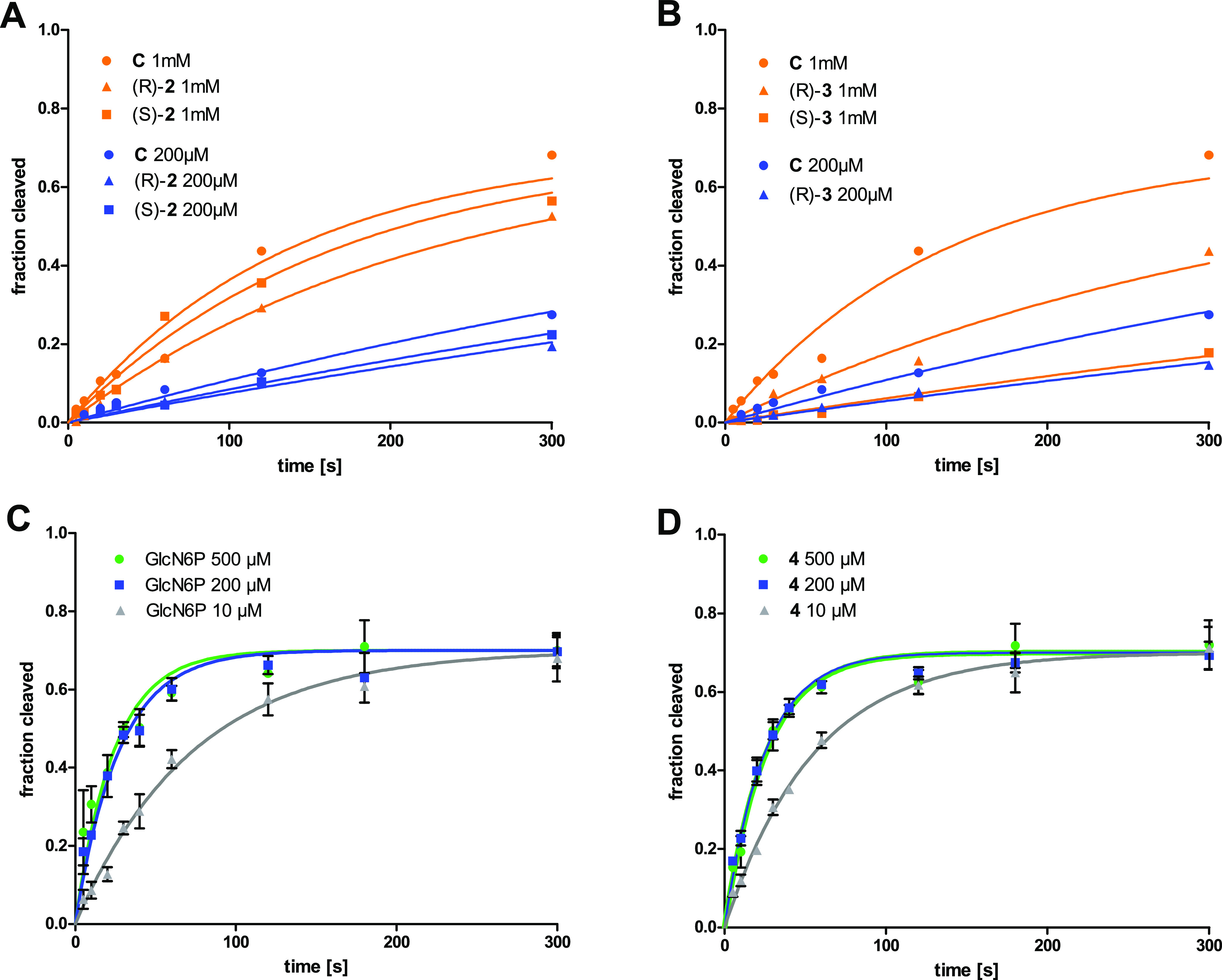
Kinetic measurements of the self-cleavage of 5′-^32^P-labeled *B. subtilisglmS* ribozyme induced by (A) hydroxyphosphonates (*R*)-**2** and (*S*)-**2** and methylene phosphonate **C**, (B) fluorophosphonates (*R*)-**3** and (*S*)-**3** and methylene phosphonate **C**, (C) GlcN6P, and (D) thia-GlcN6P **4**. All measurements performed at the same concentration are represented with the same color. The error bars in (C) and (D) represent the standard deviation of three independent measurements.

To gain further insights into the cleavage activation efficiency of the most promising compounds, we performed kinetic measurements with (*R*)-**2**, (*S*)-**2**, (*R*)-**3**, (*S*)-**3**, and **4**. The natural metabolite GlcN6P served as a control. In addition, we prepared the literature-known methylene phosphonate **C** and included it in the kinetic investigations. 5′-^32^P-labeled *B. subtilis* ribozyme was incubated with different concentrations of the activators followed by time-resolved determination of the fraction cleaved (Figures 5A,B and S2). Since it became visible that the self-cleavage induction observed for phosphonates **C**, (*R*)-**2**, (*S*)-**2**, (*R*)-**3**, and (*S*)-**3** was less efficient compared to GlcN6P, the measurements were carried out at activator concentrations ranging from 200 μM to 1 mM. Table 1 shows the determined apparent rate constants *k*_obs_. All phosphonates turned out to be activators of the riboswitch, albeit with lower efficiency than the natural metabolite GlcN6P. Phosphonate **C**, for example, has a *k*_obs_ of 0.332 min^–1^ at 500 μM, which is 7.5-fold slower compared to the value of GlcN6P (2.49 min^–1^). A similar reduction of the activity has been reported by Soukup.^22^ The hydroxy- and fluorophosphonates (*R*)-**2**, (*S*)-**2**, (*R*)-**3**, and (*S*)-**3** are even less active. Interestingly and against our expectation, the fluorophosphonates (*R*)-**3** and (*S*)-**3** have smaller *k*_obs_ values than the hydroxyphosphonates (*R*)-**2** and (*S*)-**2** although the fluorophosphonates with a value of 6.3 ± 0.1 perfectly match the p*K*_a2_ of natural GlcN6P (6.2 ± 0.1) (Table 1). This observation indicates that a matching p*K*_a_ value of a phosphonate is not sufficient for this functional group to act as an effective phosphate mimic that can induce riboswitch self-cleavage.

**Table 1:** Rate Constants *k*_obs_ of *B. subtilisglmS* Ribozyme Cleavage Induced by Compounds (*R*)-**2**, (*S*)-**2**, (*R*)-**3**, (*S*)-**3**, Methylene Phosphonate C, Thia-GlcN6P **4**, and GlcN6P

		*k*_obs_ [min^–1^]			
compound	p*K*_a2_	@ 10 μM	@ 200 μM	@ 500 μM	@ 1 mM
GlcN6P	6.2 ± 0.1	0.820 ± 0.04	2.21 ± 0.18	2.49 ± 0.28	n.d.
**C**	7.5 ± 0.1	n.d.	0.103 ± 0.004	0.332 ± 0.01	0.439 ± 0.05
(*R*)-**2**	7.2 ± 0.1	n.d.	0.069 ± 0.004	0.211 ± 0.01	0.270 ± 0.01
(*S*)-**2**		n.d.	0.078 ± 0.004	0.157 ± 0.01	0.362 ± 0.03
(*R*)-**3**		n.d.	0.049 ± 0.002	0.174 ± 0.01	0.173 ± 0.01
(*S*)-**3**	6.3 ± 0.1	n.d.	n.d.	0.091 ± 0.004	0.055 ± 0.003
**4**		1.09 ± 0.15	2.44 ± 0.12	2.36 ± 0.04	n.d.

**Figure 6:**
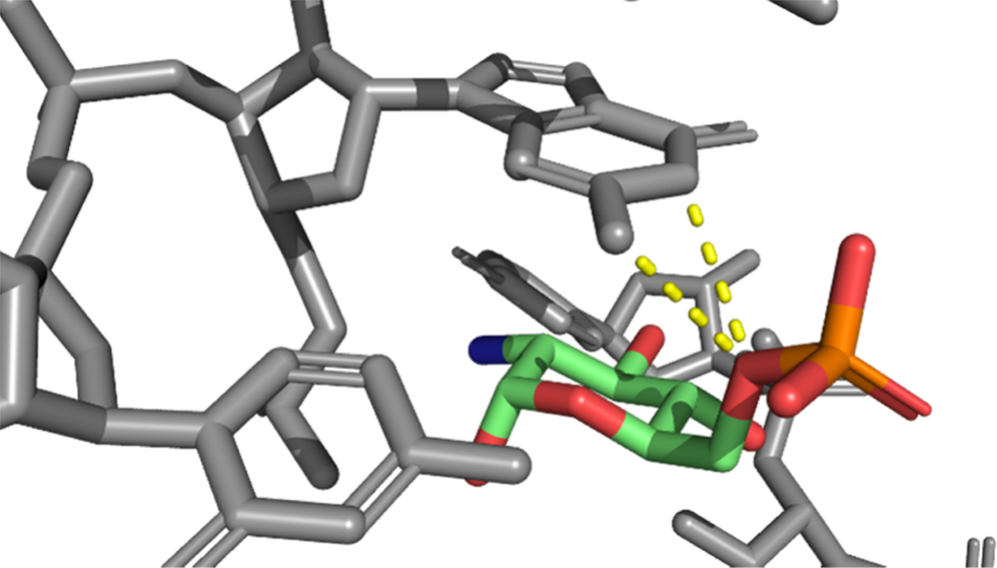
Crystal structure of the *glmS* ribozyme bound to GlcN6P (PDB code 2nz4).^37^ Two hydrogen bonds between guanine 1 and the oxygen connecting the carbohydrate core and phosphate are highlighted in yellow.

A possible explanation for the different activities of the synthesized phosphonates and the natural ligand GlcN6P becomes visible when examining the crystal structure of the *glmS* riboswitch from *Bacillus anthracis* in complex with GlcN6P (Figure 6).^37^ Binding of GlcN6P is achieved through recognition of both the phosphate and the sugar moiety, and it is reported that all three nonbridging oxygens of the phosphate make contacts with the hydrated Mg^2+^ ions, and one of these oxygens makes a direct contact to N1 of guanine 1 (G1).^37^ When we examined this structure, we realized that also, the bridging (phosphorylated) oxygen in the 6-position is involved in two hydrogen bonds to G1, one to N1 and one to the NH_2_ group at C2 (Figure 6). In the case of phosphonates, such a hydrogen bond is not possible. In hydroxyphosphonates and fluorophosphonates, the hydroxy and fluoro substituents, in principle, could take over the role as a hydrogen bond acceptor. However, in the complex with the ribozyme, neither of the two possible stereoisomers with either *S*- or *R*-configuration would position the OH or F substituent in a suitable position to make two hydrogen bonds. Therefore, the presence of oxygen at the 6-position seems to be more important than initially thought.

We next determined the apparent rate constants *k*_obs_ of the self-cleavage reaction induced by thia-GlcN6P **4** at 10, 200, and 500 μM (Figure 5C,D, Table 1) and found virtually identical rate constants as with the natural ligand GlcN6P. This finding is remarkable and demonstrates that thia-GlcN6P **4** is a very potent mimic of GlcN6P. Similar results were obtained with *glmS* ribozyme constructs from *Listeria monocytogenes* and *Clostridium difficile* (Figure S3), demonstrating that several *glmS* ribozymes accept thia-GlcN6P **4** as a ligand. With thia-GlcN6P **4**, we discovered a new artificial ligand of the *glmS* ribozyme that can activate the self-cleaving reaction, rivaling the activity of the natural ligand.

### Antimicrobial Activity of *glmS* Ligand Analogues

The promising properties of the GlcN6P mimics to induce self-cleavage of the *glmS* ribozyme, especially that of thia-GlcN6P **4**, prompted us to investigate whether these compounds possess antimicrobial activity. Accordingly, we carried out growth inhibition assays. To be biologically active, the compounds must be taken up by the bacteria. For polar and charged compounds, such as the phosphonates and phosphates described in this work, we did not expect that they would passively diffuse into bacteria. Thia-GlcN **11** was expected to be a substrate for phosphotransferase transporter systems (PTSs) that couple active uptake with phosphorylation of the 6-hydroxy group, thereby producing thia-GlcN6P **4**. Therefore, we included the thiasugar in its unphosphorylated form. The same strategy was successful when the antimicrobial properties of carba-GlcN were investigated.^5^ Such a strategy is not possible for the non-natural phosphonates **1**, **2**, and **3**. Nevertheless, we included selected examples in our investigations as unprotected phosphonates, expecting that this might impede their uptake and potential antimicrobial activity.

To estimate the antimicrobial potential of the synthesized compounds, we performed filter disk assays. Chloramphenicol (*Cm*), a known antibiotic, was used as the positive control. GlcN, which is converted by the PTS system to the natural ligand GlcN6P during uptake, was expected to have no effect on bacterial growth and was also included. The results are shown in Figure S4. For the positive control, chloramphenicol, we observed a clear inhibition zone. As expected, hydroxyphosphonate (*R*)-**2**, fluorophosphonate (*S*)-**3**, thia-GlcN6P **4**, and GlcN did not result in any growth inhibition for all bacterial strains tested. For thia-GlcN **11**, however, we observed clear growth inhibition for *B. subtilis* and *B. thuringiensis*, while no growth inhibition was observed for *Escherichia coli*. The two *Bacillus* strains are known to contain a *glmS* ribozyme, whereas *E. coli* as a member of Gram-negative bacteria lacks this riboswitch. However, this correlation could be a coincidence, and further experiments are needed to shed light on the mechanism of the antibacterial effect of thia-GlcN **11**. To quantify the antibiotic activity, we determined the minimal inhibitory concentration (MIC) for both *Bacillus* strains. These experiments revealed a MIC of thia-GlcN **11** of 460 μg mL^–1^ toward *B. subtilis* and 1.15 mg mL^–1^ toward *B. thuringiensis*.

## Conclusions

In summary, we presented the synthesis and biological evaluation of a series of GlcN6P mimics with either phosphonate structure or ring oxygen replaced with sulfur. By varying the substitution pattern of the phosphonate methylene group (C-7 of the GlcN6p mimics), we generated mimics with varying acidity of the phosphonate group. Since it is known that the interaction of the phosphate of the natural metabolite GlcN6P with the hydrated Mg^2+^ ions present in the complex is important for the recognition by the ribozyme, we expected that a phosphonate mimic with the same p*K*_a2_ value could result in an efficient activator. However, it turned out that all synthesized phosphonate mimics, even the fluorophosphonate (*S*)-**3** with the same p*K*_a2_ as GlcN6P, were less active. A likely explanation became obvious when examining the known X-ray structure of the ribozyme in complex with GlcN6P. In this structure, contacts between O6 of GlcN6P and G1 are visible. In phosphonates, where the O6 atom is replaced with a carbon, these hydrogen bonds are missing. From this result, we conclude that phosphonate mimics are not a suitable approach to designing GlcN6P mimics. The thiasugar analogue thia-GlcN6P **4** on the other hand turned out to be a *glmS* riboswitch activator with the same activity as GlcN6P. Furthermore, the unphosphorylated thiasugar thia-GlcN **11**, which is supposed to be taken up by cells through the PTS under concomitant phosphorylation to yield thia-GlcN6P **4**, turned out to have antimicrobial activity against *B. subtilis* and *B. thuringiensis* and thus presents a promising starting point for the development of novel antibiotics.

## Materials and Methods

### General

Reactions were carried out under a nitrogen atmosphere if necessary, using the Schlenk technique. Dry solvents were prepared by common methods or purchased from Sigma-Aldrich or Acros Organics. Chemicals were purchased from Acros Organics, Sigma-Aldrich, TCI Chemicals Europe, abcr, or Carbosynth and used without further purification. Technical solvents were distilled prior to use. High-resolution mass spectra (HRMS) were recorded on a micrOTOF II ESI (Bruker) or an LTQ Orbitrap Velos mass spectrometer from Thermo Scientific. Data analysis and calculation of the expected masses were performed with Compass DataAnalysis 4.0 from Bruker. Samples were dissolved in water, acetonitrile, or mixtures of both. Preparative high-performance liquid chromatography (HPLC) was performed on an LC-20A device from Shimadzu containing the following components: degasser DGU-20A3, auto sampler SIL-20A, pumps LC-20AT, column oven CTO-20AC, controller CMB-20A, and photodiode array detector SPD-M20A. Columns and eluents are mentioned in the synthesis procedures. Data analysis was performed with LCsolution v. 1.25 from Shimadzu. Preparative flash column chromatography (FC) was carried out on silica gel 60 (Geduran Si 60; 0.040–0.063 mm particle size) from Merck. Solvent mixtures are given as the volume ratio (v/v). NMR spectra were recorded on an Avance III 400, an Avance III 600, or an Avance Neo 800 spectrometer from Bruker or a Lambda 400 or a Lambda 500 spectrometer from JEOL. The measurements were performed at RT. To assign signals, 2D NMR spectroscopy was performed (COSY, HSQC, ^1^H,^13^C-HMBC, ^1^H,^31^P-HMBC, NOESY). For data analysis, MestReNova version 12.0 from Mestrelab Resarch S.L. was used. Pseudomultiplets are marked with a “p”. Analytical thin-layer chromatography (TLC) was performed using silica-coated aluminum sheets (TLC Silica gel 60 F254) from Merck. Detection was carried out either by excitation of the fluorescence at 254 nm or by dipping in one of the following staining solutions and subsequent gentle heating. Anisaldehyde: ethanol (135 mL), conc. H_2_SO_4_ (5 mL), 4-anisaldehyde (3.7 mL), and glacial acetic acid (1.5 mL). Vanillin: ethanol (250 mL), conc. H_2_SO_4_ (2.5 mL), and vanillin (6 g). Potassium permanganate: 0.1% KMnO_4_ in 1 N NaOH.

### General Procedure A: Deprotection of Phosphonates Followed by Hydrogenation

The benzylated ethyl phosphonate is dissolved in CDCl_3_ (20 mL mmol^–1^). TMSBr (80 equiv) is added, the reaction is stirred overnight, and volatiles are removed under reduced pressure. The residue is dissolved in MeOH, and the solvent is removed under reduced pressure. This step is repeated three times. The crude deprotected phosphonate is dissolved in MeOH (20 mL mmol^–1^), and 10% Pd–C (water wet, 35% w/w of sugar) is added. The reaction mixture is placed into a laboratory autoclave and stirred under 10 bar hydrogen pressure until HPLC monitoring shows complete consumption of the starting material. The catalyst is removed by filtration through a plug of Celite followed by filtration through a regenerated cellulose syringe filter. The solvent is removed under reduced pressure to give the target compound. The compound is purified by HILIC HPLC or FC using a cellulose-stationary phase, followed by multiple cycles of lyophilization. Specific procedures are given for each compound.

### Synthesis of Hydroxyphosphonates

#### 2-((2*R*,3*R*,4*R*,5*R*,6*R*)-3,4,6-Tris(benzyloxy)-5-(dibenzylamino)tetrahydro-2*H*-pyran-2-yl)acetonitrile (**7**)

Alcohol **5** (5.7 g, 9.1 mmol) was dissolved in DCM (40 mL) and cooled to −40 °C. Lutidine (1.56 mL, 13.5 mmol) and trifluoromethanesulfonic anhydride (2.3 mL, 13.6 mmol) were slowly added. The resulting mixture was stirred for 45 min, and the reaction stopped by addition of 1 M NaHSO_4_ (150 mL). The aqueous layer was extracted with DCM (2 × 50 mL), and the combined organic layers were dried over MgSO_4_ and concentrated under reduced pressure. The resulting residue was used without further purification in the next step. The crude triflate (6.9 g, 9.1 mmol) was dissolved in MeCN (90 mL) at RT. KCN (5.9 g, 91 mmol) was suspended in water (15 mL) and added to the reaction mixture. The reaction was stirred overnight, the solvent was removed under reduced pressure, and the residue was purified by FC (petroleum ether/EtOAc = 8:1 to 5:1) to give **7** (4.3 g, 6.8 mmol, 75% o2s) as a colorless solid. *R*_f_ = 0.59 (petroleum ether/EtOAc = 5:1); ^1^H NMR (CDCl_3_, 500 MHz) δ [ppm] = 7.57–7.07 (m, 25H, arenes), 5.05 (d, 1H, *J* = 11.1 Hz, O-CHHPh), 4.97 (d, 1H, *J* = 11.6 Hz, O-CHHPh), 4.81 (m, 2H, O-CH_2_Ph), 4.69 (d, 1H *J* = 7.9 Hz, H-1), 4.66 (d, 1H, *J* = 11.6 Hz, O-CHHPh), 4.51 (d, 1H, *J* = 11.1 Hz, O-CHHPh), 3.92 (d, 2H, *J* = 13.7 Hz, N-CH_2_Ph), 3.77 (d, 2H, *J* = 13.7 Hz, N-CH_2_Ph), 3.73 (dd, 1H, *J* = 9.9, 8.4 Hz, H-3), 3.47 (ddd, 1H, *J* = 9.6, 8.6, 3.1 Hz, H-5), 3.32 (dd, 1H, *J* = 9.6, 8.4 Hz, H-4), 3.03 (dd, 1H, *J* = 9.9, 7.9 Hz, H-2), 2.66 (dd, 1H, *J* = 16.8, 3.1 Hz, H-6), 2.36 (dd, 1H, *J* = 16.8, 8.6 Hz, H-6); ^13^C NMR (CDCl_3_, 126 MHz) δ [ppm] = 139.5, 138.8, 137.6, 137.0, 129.0, 128.9, 128.8, 128.7, 128.5, 128.4, 128.3, 128.2, 127.6, 127.4, 127.0, 117.4 (CN), 100.3 (C-1), 81.5 (C-4), 81.1 (C-3), 75.2 (O-CH_2_Ph), 74.6 (O-CH_2_Ph), 70.9 (O-CH_2_Ph), 70.7 (C-5), 63.5 (C-2), 54.9 (2 × N-CH_2_Ph), 21.5 (C-6); HRMS (ESI) *m*/*z* calcd for C_42_H_42_N_2_O_4_: 639.3217 [*M* + H^+^], found: 639.3212.

#### Diethyl ((*R*)-**1**-hydroxy-2-((2*R*,3*R*,4*R*,5*R*,6*R*)-3,4,6-tris(benzyloxy)-5-(dibenzylamino)tetrahydro-2*H*-pyran-2-yl)ethyl)phosphonate ((*R*)-**8**) and Diethyl ((*S*)-**1**-hydroxy-2-((2*R*,3*R*,4*R*,5*R*,6*R*)-3,4,6-tris(benzyloxy)-5-(dibenzylamino)tetrahydro-2*H*-pyran-2-yl)ethyl)phosphonate ((*S*)-**8**)

Nitrile **7** (2.8 g, 4.4 mmol) was dissolved in DCM (60 mL) and cooled to −78 °C. DIBAL-H (1 M in toluene, 13.2 mmol, 13.2 mL) was added slowly. The resulting mixture was stirred for 1 h, and the reaction was stopped by the addition of 1 M HCl (60 mL). The organic phase was washed with 1 M HCl (2 × 50 mL) and brine (50 mL), dried over MgSO_4_, and concentrated under reduced pressure. The crude aldehyde was used without further purification in the next step. The crude aldehyde (2.8 g, 4.4 mmol) was dissolved in THF (60 mL) and cooled to −78 °C. In another flask, diethyl phosphite (1.1 g, 7.8 mmol) was dissolved in THF (60 mL) and cooled to −78 °C. LiHMDS (1 M in THF, 5.6 mL, 5.5 mmol) was added to the phosphite, and the solution was stirred for 15 min. The cooled aldehyde solution was added slowly to the resulting mixture, and the reaction was stirred for 30 min and stopped by the addition of a saturated NH_4_Cl solution. The aqueous layer was extracted with Et_2_O (3 × 50 mL), and the combined organic layers were washed with brine (50 mL), dried over MgSO_4_, and concentrated under reduced pressure. The residue was purified by FC (petroleum ether/EtOAc = 1:1 to 1:3) to give **8** (1.9 g, 2.42 mmol, 55% o2s) as a colorless oil as a 3:2 separable mixture of diastereomers.

(*R*)-**8** (major isomer): *R*_f_ = 0.52 (petroleum ether/EtOAc = 1:2); ^1^H NMR (CDCl_3_, 500 MHz) δ [ppm] = 7.48–7.16 (m, 25H), 5.02 (d, 1H, *J* = 11.8 Hz, O-CHHPh), 4.88 (d, 1H, *J* = 11.6 Hz, O-CHHPh), 4.83 (d, 1H *J* = 11.8 Hz, O-CHHPh), 4.79 (d, 1H, *J* = 10.8 Hz, O-CHHPh), 4.69 (d, 1H, *J* = 8.3 Hz, H-1), 4.58 (m, 2H, O-CH_2_Ph), 4.17 (m, 5H, 2 × PCH_2_, CHOH), 3.93 (d, 2H, *J* = 13.7 Hz, N-CH_2_Ph), 3.78 (d, 2H *J* = 13.7 Hz, N-CH_2_Ph), 3.74 (dd, 1H, *J* = 10.1, 8.4 Hz, H-3), 3.55 (td, 1H, *J* = 9.4, 3.0 Hz, H-5), 3.34 (dd, 1H, *J* = 9.6, 8.4 Hz, H-4), 3.06 (dd, 1H, *J* = 16.7, 3.1 Hz, OH), 3.00 (dd, 1H, *J* = 10.0, 8.2 Hz, H-2), 2.39 (m, 1H, H-6), 1.91 (m, 1H, H-6), 1.32 (td, 6H, *J* = 7.1, 5.5 Hz, 2 × CH_3_); ^13^C NMR (CDCl_3_, 126 MHz) δ [ppm] = 139.7, 139.0, 138.0, 137.2, 129.0, 128.7, 128.6, 128.6, 128.5, 128.3, 128.3, 128.1, 128.0, 127.5, 127.4, 127.0, 101.2 (C-1), 83.2 (C-4), 81.2 (C-3), 75.3 (C-5) 75.2 (O-CH_2_Ph), 74.6 (O-CH_2_Ph), 71.1 (O-CH_2_Ph), 63.4 (C-2), 62.8 (2 × CH_2_P), 55.1 (2 × N-CH_2_Ph), 33.5 (C-6), 16.7 (CH_3_); ^31^P NMR (CDCl_3_, 202 MHz) δ [ppm] = 23.68; HRMS (ESI) *m*/*z* calcd for C_46_H_54_NO_8_P: 780.3660 [*M* + H^+^], found: 780.3659.

(*S*)-**8** (minor isomer): *R*_f_ = 0.45 (petroleum ether/EtOAc = 1:2); ^1^H NMR (CDCl_3_, 400 MHz) δ [ppm] = 7.55–7.12 (m, 25H), 5.03 (d, 1H, *J* = 11.2 Hz, O-CHHPh), 4.94 (d, 1H, *J* = 11.8 Hz, O-CHHPh), 4.84 (d, 1H, *J* = 11.2 Hz, O-CHHPh), 4.80–4.67 (m, 3H, O-CHHPh, H-1), 4.54 (d, 1H *J* = 11.2 Hz, O-CHHPh), 4.23–4.08 (m, 5H, 2 × PCH_2_, CHOH), 3.94 (d, 2H, *J* = 13.8 Hz, N-CH_2_Ph), 3.85–3.67 (m, 4H, H-4, H-5, N-CH_2_Ph), 3.34 (m, 1H, OH), 3.28 (dd, 1H, *J* = 9.7, 8.3 Hz, H-3), 3.00 (dd, 1H, *J* = 10.1, 8.3 Hz, H-2), 2.24 (m, 1H, H-6), 1.83 (m, 1H, H-6), 1.31 (td, 6H, *J* = 7.1, 3.4 Hz, CH_3_); ^13^C NMR (CDCl_3_, 126 MHz) δ [ppm] = 139.8, 139.1, 138.2, 137.5, 129.00, 128.8, 128.6, 128.46, 128.4, 128.2, 128.1, 128.0, 127.8, 127.4, 126.8, 101.1 (C-1), 83.2 (C-3), 81.4 (C-4), 75.0 (O-CH_2_Ph), 74.7 (O-CH_2_Ph), 70.94 (O-CH_2_Ph), 70.86 (C-5), 64.6 (d, *J* = 163.6 Hz), 63.4 (C-2), 62.8 (2 × PCH_2_), 54.9 (2 × N-CH_2_Ph), 33.3 (C-6), 16.6 (CH_3_); ^31^P NMR (CDCl_3_, 202 MHz) δ [ppm] = 25.33; HRMS (ESI) *m*/*z* calcd for C_46_H_54_NO_8_P: 780.3660 [*M* + H^+^], found: 780.3653.

#### (2-((2*R*,3*S*,4*R*,5*R*)-5-Amino-3,4,6-trihydroxytetrahydro-2*H*-pyran-2-yl)-1-hydroxyethyl)phosphonic acid bis(triethylammonium) salt ((*R*)-**2**·2 NEt_3_)

Ethyl phosphonate (*R*)-**8** (300 mg, 384 μmol) was treated according to general procedure A. The residue was purified by HILIC HPLC (Phenomenex Luna 5 μm HILIC 200 Å, AXIA Pa, 250 × 21.20 mm^2^, 50% MeCN to 40% MeCN in 15 mM TEAB buffer pH 7.00 in 15 min, 10.0 mL min^–1^, ELSD) to yield bis(triethylammonium) hydroxyphosphonate (*R*)-**2**·2 NEt_3_ (132 mg, 276 μmol, 72%) as a colorless solid. ^1^H NMR (D_2_O, 500 MHz) δ [ppm] = 5.42 (d, 1H, *J* = 3.7 Hz, H-1 α isomer), 4.91 (d, 1H, *J* = 8.4 Hz, H-1 β isomer), 4.09 (ddd, 1H *J* = 10.4, 7.8, 3.4 Hz, 1 × H-5), 3.99–3.89 (m, 2H, HC-P), 3.86 (dd, 1H *J* = 10.6, 9.1 Hz, H-3 α isomer), 3.70 (ddd, 1H, *J* = 10.7, 7.6, 3.1 Hz, 1 × H-5), 3.66–3.59 (m, 1H, H-3 β isomer), 3.40 (m, 2H, 2 × H-4), 3.34–3.28 (dd, 1H, *J* = 10.6, 3.7 Hz, H-2 α isomer), 3.22 (q, 24H, *J* = 7.4 Hz, CH_2_N), 3.04–2.95 (dd, 1H, *J* = 10.4, 8.4 Hz, H-2 β isomer), 2.34 (m, 2H, H-6), 1.91–1.72 (m, 2H, H-6), 1.30 (t, *J* = 7.3 Hz, 36H, CH_3_); ^13^C NMR (D_2_O, 126 MHz) δ [ppm] = 93.1 (C-1 β), 89.3 (C-1 α), 74.7 (1 × C-5), 74.0 (C-4), 72.3 (C-3 β), 70.7 (1 × C-5), 69.7 (C-3 α), 57.0 (C-2 β), 54.5 (C-2 α), 46.8 (CH_2_N), 34.3 (C-6), 8.3 (CH_3_); ^31^P NMR (D_2_O, 202 MHz) δ [ppm] = 19.33; HPLC: *t*_R_ = 6.5 min (Phenomenex Luna 5 μm HILIC 200 Å, AXIA Pa, 250 × 21.20 mm^2^, 50% MeCN to 40% MeCN in 15 mM TEAB buffer pH 7.00 in 15 min, 10.0 mL min^–1^, ELSD); HRMS (ESI) *m*/*z* calcd for C_7_H_16_NO_8_P: 272.0541 [*M* – H^+^], found: 272.0543.

#### (2-((2*R*,3*S*,4*R*,5*R*)-5-Amino-3,4,6-trihydroxytetrahydro-2*H*-pyran-2-yl)-1-hydroxyethyl)phosphonic acid bis(triethylammonium) salt ((*S*)-**2**·2 NEt_3_)

Ethyl phosphonate (*S*)-**8** (250 mg, 321 μmol) was treated according to general procedure A. The residue was purified by HILIC HPLC (Phenomenex Luna 5 μm HILIC 200 Å, AXIA Pa, 250 × 21.20 mm^2^, 40% MeCN in 15 mM TEAB buffer pH 7.00 for 15 min, 10.0 mL min^–1^, ELSD) to yield the bis(triethylammonium) hydroxyphosphonate (*S*)-**2**·2 NEt_3_ (119 mg, 250 μmol, 82%) as a colorless solid. ^1^H NMR (D_2_O, 500 MHz) δ [ppm] = 5.31 (d, 1H, *J* = 3.7 Hz, H-1 α isomer), 4.80 (d, 1H, *J* = 8.4 Hz, H-1 β isomer), 3.94 (t, 1H *J* = 10.1 Hz, 1H, 1 × H-5), 3.77 (m, 4H, 2 × H-3, 2 × OCHP), 3.60–3.47 (m, 3H, 2 × H-3, 1 × H-5), 3.27–3.17 (m, 3H, H-2 β isomer, 2 × H-4), 3.14–3.05 (q, 24H, *J* = 7.3 Hz, CH_2_N) 2.90 (m, 1H, *J* = 10.6, 8.4 Hz, H-2 α isomer), 2.02 (m, 2H, 2 × H-6), 1.73 (m, 2H, 2 × H-6), 1.24–1.08 (t, 36H, *J* = 7.3 Hz, CH_3_); ^13^C NMR (D_2_O, 126 MHz) δ [ppm] = 93.0 (C-1 β), 89.2 (C-1 α), 73.6 (C-4), 72.2 (C-3), 71.9 (1 × C-6), 69.8, 67.4 (1 × C-6), 63.9 (C–P), 57.1 (C-2 α), 54.6 (C-2 β), 47.30 (CH_2_N), 33.5 (C-6), 7.6 (CH_3_); ^31^P NMR (D_2_O, 202 MHz) δ [ppm] = 20.10; HPLC: *t*_R_ = 7.5 min (Phenomenex Luna 5 μm HILIC 200 Å, AXIA Pa, 250 × 21.20 mm^2^, 40% MeCN in 15 mM TEAB buffer pH = 7.00 for 15 min, 10.0 mL min^–1^, ELSD); HRMS (ESI) *m*/*z* calcd for C_7_H_16_NO_8_P: 272.0541 [*M* – H^+^], found: 272.0544.

### Synthesis of Thia-glucosamine-6-phosphate

#### *tert*-Butyl ((3*R*,4*R*,5*S*,6*R*)-2,4,5-trihydroxy-6-(hydroxymethyl)tetrahydro-2*H*-thiopyran-3-yl)carbamate (**14**)

Thiasugar **11**·HCl (190 mg, 820 μmol) was dissolved in MeOH (3 mL). Boc_2_O (215 mg, 215 μmol) was added, followed by the addition of KOH (96 mg, 1.7 mmol). The resulting solution was stirred for 4 h. The solution was diluted with H_2_O (4 mL) and carefully neutralized by the addition of 0.1 M HCl. The solvent was removed under reduced pressure, and the residue was purified by FC (DCM/MeOH: 2–15% MeOH) to give **14** (188 mg, 637 μmol, 78%) as a colorless solid. ^1^H NMR (CD_3_OD, 400 MHz) δ [ppm] = 6.32 (d, 1H, *J* = 8.6 Hz, H-1 β isomer), 4.90 (d, 1H, *J* = 2.8 Hz, H-1 α isomer), 4.00–3.70 (m, 3H), 3.66–3.51 (m, 2H), 3.26 (m, 1H), 1.47 (s, 10H, CH_3_); ^13^C NMR (CD_3_OD, 101 MHz) δ [ppm] = 80.4, 76.8, 73.8, 73.6, 62.6, 61.2, 44.8, 28.7; HRMS (ESI) *m*/*z* calcd for C_11_H_21_NO_6_S: 318.0982 [*M* + Na^+^], found: 318.0978.

#### *tert*-Butyl ((3*R*,4*R*,5*S*,6*R*)-6-(((diethoxyphosphoryl)oxy)methyl)-2,4,5-trihydroxytetrahydro-2*H*-thiopyran-3-yl)carbamate (**15**)

Boc-protected thiasugar **14** (180 mg, 609 μmol) was dissolved in pyridine (5 mL) and cooled to −50 °C. Diethyl chlorophosphate (124 μL, 853 μmol) was slowly added. After 1.5 h, the reaction was stopped by the addition of MeOH (4 mL). Volatiles were removed under reduced pressure, and the crude product was purified by FC (DCM/MeOH: 0–10% MeOH) to give **15** (163 mg, 377 μmol, 62%) as a colorless solid. ^1^H NMR (CD_3_OD, 500 MHz) δ [ppm] = 4.91 (d, 1H *J* = 2.6 Hz, 1H, H-1), 4.41 (ddd, 1H *J* = 10.9, 4.8, 5.0 Hz, H-6), 4.30 (ddd, 1H, *J* = 10.9, 5.5, 2.2 Hz, H-6), 4.15 (m, 4H, CH_2_OP), 3.79–3.71 (m, 1H, H-2), 3.62–3.53 (m, 2H, H-3, H-4), 3.36 (ddd, 1H, *J* = 9.9, 4.8, 2.2 Hz, H-5), 1.45 (s, 9H, CH_3_–Boc), 1.35 (m, 6H, 2 × CH_3_); ^13^C NMR (CD_3_OD, 126 MHz) δ [ppm] = 157.6 (CO), 79.9 (C-quart), 75.0 (C-4), 73.5 (C-3), 73.2 (C-1), 67.2 (C-6), 65.1 (CH_2_OP), 60.8 (C-2), 42.5 (C-5), 28.3 (CH_3_–Boc), 16.0 (CH_3_-ethyl); ^31^P (CD_3_OD, 202 MHz,) δ [ppm] = −0.98; HRMS (ESI) *m*/*z* calcd for C_15_H_30_NO_9_PS: 454.1271 [*M* + Na^+^], found: 454.1270.

#### ((2*R*,3*S*,4*R*,5*R*)-5-Amino-3,4,6-trihydroxytetrahydro-2*H*-thiopyran-2-yl)methyl dihydrogen phosphate bis(triethylammonium) salt (**4**·2 NEt_3_)

Ethyl phosphate **15** (130 mg, 301 μmol) was dissolved in CDCl_3_ (3 mL). TMSBr (1 mL) was added, and the reaction mixture was stirred for 1 h. TFA (1 mL) was added to the reaction mixture, and the mixture was stirred for 5 min. The volatiles were removed under reduced pressure, and the residue was dissolved in H_2_O (2 mL), which was removed under reduced pressure. The crude residue was dissolved in 0.1 M HCl (1 mL), and the pH of the solution was adjusted to 4–5 using Ba(OH)_2_. EtOH was added, until a precipitate formed. The mixture was centrifuged, and the solvent was removed by decantation. The precipitation process was repeated three times. The precipitate of crude **16** was purified by HILIC-HPLC to yield bis(triethylammonium) thia-glucosamine-6-phosphate **4**·2 NEt_3_ (101 mg, 211 μmol, 70%) as a colorless solid.

To facilitate signal assignment, we recorded 800 MHz ^1^H NMR and 201 MHz ^13^C NMR spectra with NEt_3_ suppression. In the Supporting Information, spectra without NEt_3_ suppression are also depicted. α-Isomer: ^1^H NMR (D_2_O, 800 MHz) δ [ppm] = 5.13 (d, 1H, *J* = 3.1 Hz, H-1), 4.18 (ddd, 1H, *J* = 11.5, 6.8, 4.8 Hz, H-6), 3.90 (ddd, 1H, *J* = 11.5, 5.6, 2.5 Hz, H-6), 3.76 (pt, 1H, *J* = 9.8 Hz, H-3), 3.71 (pt, 1H, *J* = 9.8 Hz, H-4), 3.55 (dd, 1H, *J* = 9.8, 3.1 Hz, H-2), 3.27 (m, 1H, H-5); ^13^C NMR (D_2_O, 201 MHz) δ [ppm] = 72.9 (C-4), 70.3 (C-3), 70.0 (C-1), 62.6 (C-6), 58.6 (C-2), 42.0 (C-5); ^31^P NMR (162 MHz, D_2_O) δ [ppm] = 3.29. β-Isomer: ^1^H NMR (D_2_O, 800 MHz) δ [ppm] = 4.97 (d, 1H *J* = 9.8 Hz, H-1), 4.10 (m, 1H, H-6), 3.95 (m, 1H, H-6), 3.66 (m, 1H, H-4 signal overlap with residual NEt_3_), 3.42 (pt, 1H, *J* = 9.7 Hz, H-3), 3.32 (m, 1H, H-2), 3.02 (m, 1H, H-5); ^13^C NMR (D_2_O, 201 MHz) δ [ppm] = 72.7 (C-3), 72.6 (C-4), 70.5 (C-1), 62.5 (C-6), 60.8 (C-2), 44.4 (C-5); ^31^P NMR (D_2_O, 162 MHz) δ [ppm] = 3.29; HPLC: *t*_R_ = 6.6 min (Phenomenex Luna 5 μm HILIC 200 Å, AXIA Pa, 250 × 21.20 mm^2^, 40% MeCN in 15 mM TEAB buffer pH 6.99 for 15 min, 10.0 mL min^–1^, ELSD); HRMS (ESI) *m*/*z* calcd for C_6_H_14_NO_7_PS: 274.0156 [*M* – H^+^], found: 274.0157.

## Supporting Information Available

Assignment of diastereomers by Mosher ester analysis, determination of p*K*_a_ values, synthesis and preparation of RNA, kinetic self-cleavage assay, antimicrobial activity of *glmS* ligand analogues, additional syntheses, and NMR spectra (PDF)
