# Supplementary material for: Phosphonate and Thiasugar Analogues of Glucosamine-6-phosphate: Activation of the glmS Riboswitch and Antibiotic Activity
Source: ACS Chem Biol. 2023 Oct 4;18(10):2324–34. doi: 10.1021/acschembio.3c00452 (PMC10594590; doi:10.1021/acschembio.3c00452)
Supplement: Supplementary file 1 — cb3c00452_si_001.pdf [file cb3c00452_si_001.pdf]

Supporting Information to

Phosphonate and Thiasugar Analogues of Glucosamine-6-phosphate: Activation of the *glmS* Riboswitch and Antibiotic Activity

Bjarne Silkenath,† Dennis Kläge,† Hanna Altwein,† Nina Schmidhäuser,† Günter Mayer,‡ Jörg S. Hartig,*,† and Valentin Wittmann*,†

† Department of Chemistry, University of Konstanz, 78457 Konstanz, Germany; ‡ LIMES Institute, Center for Aptamer Research & Development, University of Bonn, 53121 Bonn, Germany

* Corresponding authors: J. S. Hartig, Email: joerg.hartig@uni-konstanz.de; V. Wittmann, Email: mail@valentin-wittmann.de

Content

1. Assignment of Diastereomers by Mosher Ester Analysis 2
2. Determination of pK_a Values 2
3. Synthesis and Preparation of RNA 3
4. Kinetic Self-Cleavage Assay 4
5. Antimicrobial Activity of *glmS* Ligand Analogues 5
6. Syntheses 6
7. NMR Spectra 15
8. References 52

1. Assignment of Diastereomers by Mosher Ester Analysis

For determination of the stereochemistry at C-7 of the hydroxyphosphonates **8**, the (*S*)- and the (*R*)-MTPA ester of both diastereomers of compound **8** were prepared and the chemical shift differences $\Delta\delta^{SR} = \delta^{S} - \delta^{R}$ of all signals were determined. Assignment of the absolute configuration was carried out empirically as described.[1-2]

Table S1. Chemical shifts (in ppm) of ^{1}H and ^{31}P resonances of the synthesized (*S*)-MTPA esters (δ^{S}) and (*R*)-MTPA esters (δ^{R}). Chemical shift differences were determined according to the formula $\Delta\delta^{SR} = \delta^{S} - \delta^{R}$.

**Position**	**Major isomer, (*R*)-8**			**Minor isomer, (*S*)-8**		
	δ^{S}	δ^{R}	$\Delta\delta^{SR}$	δ^{S}	δ^{R}	$\Delta\delta^{SR}$
H1	4.63	4.71	-0.08	4.48	4.46	0.02
H2	2.92	2.95	-0.03	2.95	2.91	0.04
H3	3.67	3.73	-0.06	n.d.	n.d.	n.d.
H4	3.14	3.22	-0.08	3.23	3.16	0.07
H5	3.54	3.64	-0.1	3.63	3.54	0.09
H6	1.78	1.80	-0.02	1.94	1.86	0.08
H6’	2.43	2.51	-0.08	2.41	2.3	0.11
$P(OC\underline{H_2}CH_3)_2$	4.08	4.06	0.02	4.04	4.09	-0.05
$P(OCH_2C\underline{H_3})_2$	1.25	1.21	0.04	1.24	1.26	-0.02
^{31}P NMR	19.41	18.73	0.68	19.36	19.62	-0.26

2. Determination of *pK*$_a$ Values

The determination of the pK_a value of the phosphonates was carried out according to an adapted procedure published by Baker.[3] The compound of interest was dissolved at a concentration of 33 mM in a 90 % H_2O/10 % D_2O mixture. The pH was adjusted to approx. 3 and then stepwise increased to approx. 11. At each pH, ^{31}P NMR spectra were recorded using an NMR tube with a capillary containing 85 % phosphoric acid as external standard. The obtained ^{31}P NMR chemical shifts were plotted against the pH and a sigmoidal function was fitted through the data points the point of inflection of which represents the p*K*a value.

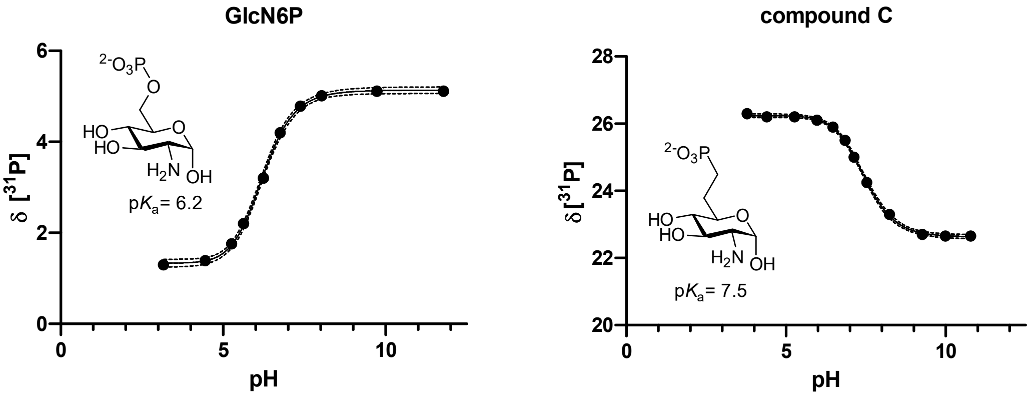

Figure S1. ^{31}P NMR titration curves of GlcN6P and methylene phosphonate **C**. Fitted sigmoidal function and 95 % confidence interval.

3. Synthesis and Preparation of RNA

The glmS-ribozyme was amplified from genomic DNA of *B. subtilis* by PCR using Phusion Plus DNA-Polymerase (Thermo Fischer). A T7 Promotor was introduced upstream using the following primers for amplification (fw: 5’-TAATACGACTCACTATAGGCCTATAATTATAGCGCC-3’) and (rv: 5’-AAGATCATGTGATTTCTC-3’). The PCR product was purified by DNA Clean & Concentrator kit (Zymo) according to manufacturers protocol. For *in vitro* transcription (37 °C, 3 h) with T7 RNA-polymerase 1 µg of DNA was used in 80 mM HEPES, 4 mM spermidine, 40 mM dithiothreitol (DTT), 24 mM $MgCl_2$ (pH7.5). The resulting RNA product was purified by denaturing polyacrylamide gel electrophoresis (PAGE). After gel extraction (10 mM HEPES 200 NaCl, 1 mM EDTA (pH7.5)) and filtering through glass whool, the RNA was precipitated adding 3 volumes ice cold EtOH (100%) (-80 °C, o/n). After centrifugation the pellet was washed with ice cold EtOH (70%). The purified RNA was dephosphorylated using recombinant shrimp alkaline phosphatase (rSAP, NEB) in 50 mM KOAc, 20 mM HEPES, 10 mM $Mg(OAc)_2$, 100 µg mL^{-1} BSA (pH 7.9). After heat inactivation (3 min, 75°C), the RNA was radioactively labelled with *γ*-^{32}P-ATP (10 mCi mL^{-1}, Hartmann Analytics) at the 5’-end using the T4 polynucleotide kinase (PNK, NEB) in 70 mM HEPES, 10 mM $MgCl_2$ and 5 mM DTT (pH 7.6). All mixtures were prepared on ice, and incubated at 37 °C for 45 min. The product was again purified by Urea-PAGE followed by gel extraction and precipitation (-80 °C, 1h).

4. Kinetic Self-Cleavage Assay

The purified radioactively labelled RNA was dissolved in ultrapurified H_2O. From here, all steps were conducted on ice. The reaction buffer (50 mM HEPES (pH 7.5), 200 mM KCl and 10 mM $MgCl_2$) was mixed with different concentrations (10 µM, 200 µM, 500 µM, 1 mM) of the respective compounds. After RNA addition the reaction was incubated at 37 °C. The cleavage reaction was stopped at different time points (5-300 s) adding PAGE loading dye (9 M Urea, 20 % w/v Sucrose, 0.1 % w/v SDS, 0.05 % w/v bromophenol blue, 0.05% w/v xylene cyanol, 90 mM Tris-HCl, 90 mM Borate, 1 mM EDTA). Samples were stored at -20 °C until they were analysed on a 10 % Urea-PAGE (40 W, 30 min). The respective bands were detected using Typhoon FLA 7000 phosphorimager (GE Healthcare Bio-Sciences AB). Data analysis was done using ImageJ and the Prism (GraphPad) software. The rate constants (k_{obs}) were determined by plotting the fraction cleaved in dependence of time and the resulting curves were obtained using a pseudo-first order association kinetic fit.

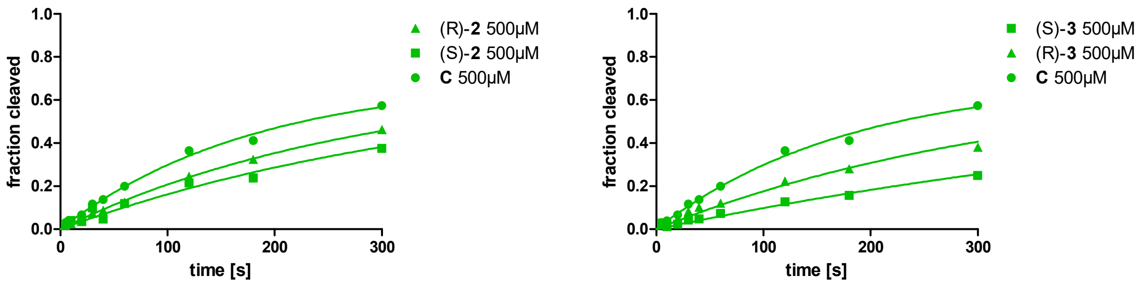

Figure S2. Kinetic measurements of the self-cleavage of 5'-^{32}P-labeled *B. subtilis glmS* ribozyme induced by hydroxyphosphonates (*R*)-**2** and (*S*)-**2** and methylene phosphonate **C** (left) and fluorophosphonates (*R*)-**3** and (*S*)-**3** and methylene phosphonate **C** (right).

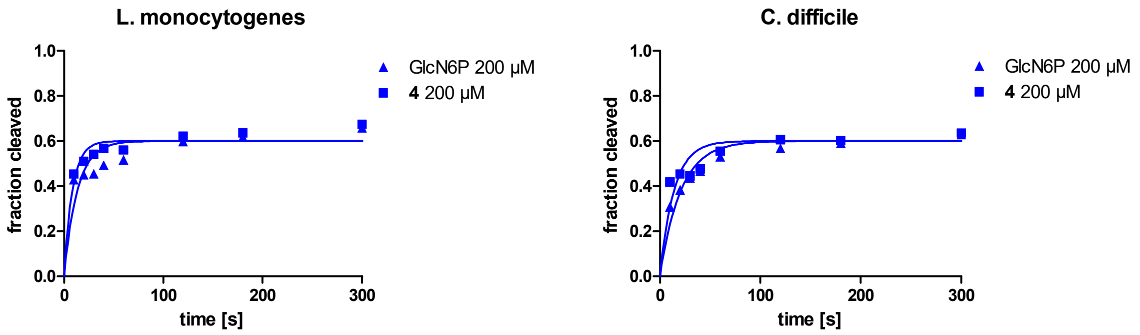

Figure S3. Kinetic measurements of the self-cleavage of 5'-^{32}P-labeled *glmS* ribozymes from *Listeria monocytogenes* (left) and *Clostridium difficile* (right) induced by GlcN6P and thia-GlcN6P **4**.

5. Antimicrobial Activity of *glmS* Ligand Analogues

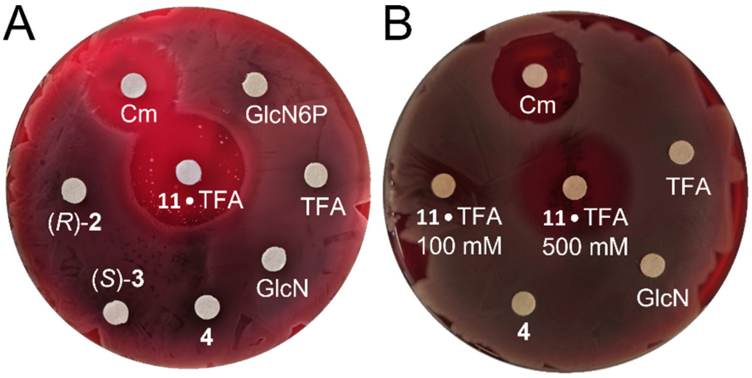

Figure S4. Filter disk assay on Mueller Hinton agar plates. A) *B. subtilis* wt 168 plated out and tested with 10 µL of the respective compound on a filter disc at a concentration of 100 mM. B) *B. thuringiensis* plated out and tested with 10 µL of the respective compound on a filter disc at a concentration of 500 mM. Cm: chloramphenicol at a concentration of 9.3 mM for both strains. TFA (trifluoroacetic acid) was tested alone since thia-GlcN **11** was employed as TFA salt.

6. Syntheses

(2-((2*R*,3*S*,4*R*,5*R*,6*R*)-5-Amino-3,4,6-trihydroxytetrahydro-2*H*-pyran-2-yl)-1,1-difluoroethyl)phosphonic acid diammonium salt (1)

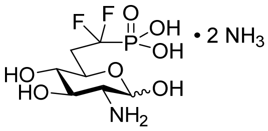

Ethyl phosphonate **6** (100 mg, 125 µmol) was treated according to general procedure A. The residue was purified by cellulose FC (eluent: 50 mM NH_4HCO_3/MeCN/*i*PrOH 2:1:2) to yield the diammonium difluorophosphonate **1** • 2 NH_3 (22.4 mg, 68 µmol, 55 %) as a colorless solid.

^{1}H NMR (D_2O, 600 MHz) δ [ppm] = 5.37 (d, 1H, *J* = 3.6 Hz, H-1 alpha), 4.90 (d, 1H, *J* = 8.4 Hz, H-1 beta), 4.29 (pt, 1H, *J* = 9.5 Hz, H-5 alpha), 3.89 (pt, 1H, *J* = 9.4 Hz, H-5 beta), 3.84 (pt, 1H, *J* = 9.8 Hz, H-3 alpha), 3.62 (pt, 1H, *J* = 9.7 Hz, H-3 beta), 3.31 (m, 2H, H-4 alpha and beta), 3.27 (dd, 1H, *J* = 10.6, 3.6 Hz, H-2 alpha), 2.96 (dd, 1H, *J* = 10.6, 8.4 Hz, H-2 beta), 2.67 – 2.50 (m, 2H, H-6), 2.30 – 2.08 (m, 2H, H-6); ^{13}C NMR (D_2O,151 MHz) δ [ppm] = 93.0 (C-1 beta), 89.1 (C-1 alpha), 72.9 (C-4 alpha and beta), 72.2(C-3 beta), 70.7 (C-5 beta), 69.7 (C-3 alpha), 66.1 (C-5 alpha), 56.8 (C-2 beta), 54.3 (C-2 alpha), 35.0 (C-6); ^{19}F NMR (D_2O, 377 MHz) δ [ppm] = -109.2, -112.41; ^{31}P NMR (D_2O, 202 MHz) δ [ppm] = 5.9. HRMS (ESI) *m*/*z* calcd for $C_7H_{14}F_2NO_7P$: 292.0403 [*M*-H$^+$], found: 292.0405.

(*R*)-2-((2*R*,3*S*,4*R*,5*R*)-5-Amino-3,4,6-trihydroxytetrahydro-2*H*-pyran-2-yl)-1-fluoroethyl)phosphonic acid diammonium salt ((*R*)-3 • 2 NH_3)

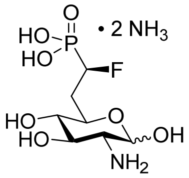

Ethyl phosphonate (*R*)-**9** (19 mg, 24 mg) was treated according to the general procedure A. The residue was purified by cellulose FC (eluent: 50 mM NH_4HCO_3/MeCN/*i*PrOH 2:1:2) to yield the diammonium fluorophosphonate (*R*)-**3** • 2 NH_3 (4.7 mg, 15.2 µmol, 62 %) as a colorless solid.

^{1}H NMR (D_2O, 500 MHz) δ [ppm] = 5.30 (d, *J* = 3.6 Hz, 1H), 4.80 (d, *J* = 8.2 Hz, 1H), 3.95 (ddd, *J* = 10.3, 7.1, 3.7 Hz, 1H), 3.73 (dd, *J* = 10.6, 9.1 Hz, 1H), 3.56 (ddd, *J* = 10.2, 6.9, 3.8 Hz, 1H), 3.50 (dd, *J* = 10.5, 8.8 Hz, 1H), 3.32 (td, *J* = 9.4, 5.1 Hz, 2H), 3.20 (dd, *J* = 10.6, 3.7 Hz, 1H), 2.88 (dd, *J* = 10.5, 8.4 Hz, 1H), 2.35 – 2.15 (m, 2H), 1.98 (dtt, *J* = 16.0, 12.3, 8.2 Hz, 1H); ^{13}C NMR (D_2O, 151 MHz) δ [ppm] = 93.0 (C-1 beta), 89.1 (C-1 alpha), 74.2, 73.4, 72.2, 70.1, 69.6 (C-3 alpha), 56.8 (C-2 beta), 54.4 (C-2 alpha), 32.5

(C-6); ^{19}F NMR (D_2O, 471 MHz) δ [ppm] = -199.13; ^{31}P NMR (D_2O, 202 MHz) δ [ppm] = 12.83 (dd, *J* = 65.4, 14.1 Hz); HRMS (ESI) *m/z* calcd for $C_7H_{15}FNO_7P$: 274.0497 [*M*-H$^+$]; found: 274.0499.

((*S*)-2-((2*R*,3*S*,4*R*,5*R*)-5-Amino-3,4,6-trihydroxytetrahydro-2*H*-pyran-2-yl)-1-fluoroethyl)phosphonic acid diammonium salt ((*S*)-3 • 2 NH_3)

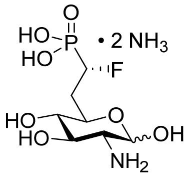

Ethyl phosphonate (*S*)-**9** (95 mg, 121 µmol) was treated according to the general procedure A. The residue was purified by cellulose FC (eluent: 50 mM NH_4HCO_3/MeCN/*i*PrOH 2:1:2) to yield the diammonium fluorophosphonate (*S*)-**3** • 2 NH_3 (25 mg, 81 µmol, 67 %) as a colorless solid.

^{1}H NMR (D_2O, 500 MHz) δ [ppm] = 5.43 (d, 1H, *J* = 3.7 Hz, H-1 alpha), 4.93 (d, 1H, *J* = 8.4 Hz, H-1 beta), 4.84-4.65 (m, 2H, HCF), 4.03 (ptd, 1H, *J* = 10.2, 1.9 Hz, 1x H-5), 3.87 (dd, 1H, *J* = 10.5, 9.1 Hz, H-3 alpha), 3.66 – 3.58 (m, 2H, H-3 beta, 1x H-5), 3.37 (pt, 2H, *J* = 9.4 Hz, 2x H-4), 3.33 (dd, 1H, *J* = 10.6, 3.7 Hz, H-2 alpha), 3.01 (dd, 1H, *J* = 10.6, 8.4 Hz, H-2 beta), 2.44– 2.31 (m, 2H, H-6), 1.97 – 1.79 (m, 2H, H-6); ^{13}C NMR (D_2O, 126 MHz) δ [ppm] = 93.2 (C-1 beta), 89.2 (C-1 alpha), 73.4 (C-4), 72.4, (C-3 beta) 71.8 (1x C-5), 69.9 (C-3 alpha), 67.2 (1x C-5), 57.1 (C-2 beta), 54.6 (C-2 alpha), 32.8 (C-6); ^{19}F NMR (D_2O, 471 MHz) δ [ppm] = -205.01; ^{31}P NMR (D_2O, 202 MHz) δ [ppm] = 12.96 (dd, *J* = 63.1, 26.8 Hz); HRMS (ESI) *m/z* calcd for $C_7H_{15}FNO_7P$: 274.0497 [*M*-H$^+$], found:274.0499.

((2*R*,3*S*,4*R*,5*R*,6*R*)-3,4,6-Tris(benzyloxy)-5-(dibenzylamino)tetrahydro-2*H*-pyran-2-yl)methanol (5)

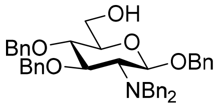

Zinc chloride (2.3 g, 16.7 mmol) was melted under vacuum in a Schlenk tube. After the salt was completely melted, it was rapidly cooled down to room temperature. Perbenzylated glucosamine[4] (2.4g, 3.33 mmol) was dissolved in a 5:1 mixture of acetic anhydride and acetic acid (15 mL) and the solution added to the fused zinc chloride. The reaction mixture was stirred overnight, and the reaction was stopped by the addition of water (100 mL). The aqueous layer was extracted with DCM (3 x 50 mL), and the combined organic layers were dried over $MgSO_4$, concentrated under reduced pressure, and purified by FC (petroleum ether/EtOAc = 9:1). The resulting acetate was dissolved in dry MeOH (50 mL), sodium methoxide (0.5 M in MeOH, 6 mmol) was added, and the mixture was stirred overnight. Amberlite IR-120® was added until a neutral pH value was reached and then

removed by filtration. The volatiles were evaporated, and the residue was purified by FC (petroleum ether/EtOAc = 3:1) to give **5** (1.45 g, 2.3 mmol, 69 % o2s) as a colorless oil. The analytical data were in accordance with the literature.[4]

Diethyl (1,1-difluoro-2-((2*R*,3*R*,4*R*,5*R*,6*R*)-3,4,6-tris(benzyloxy)-5-(dibenzylamino)tetrahydro-2*H*-pyran-2-yl)ethyl)phosphonate (6)

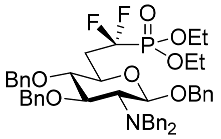

Alcohol **5** (950 mg, 1.5 mmol) was dissolved in DCM (20 mL) The solution was cooled to -40 °C and 2,6-di-tert-butyl-4-methylpyridine (464 mg, 2.3 mmol) was added. Trifluoromethanesulfonic anhydride (380 µL, 2.3 mmol) was slowly added. The solution was stirred for 1.5 h. The reaction was stopped by addition of 1 M $NaHSO_4$ (150 mL). The aqueous layer was extracted with DCM (2 x 50 mL) and the combined organic layers were dried over $MgSO_4$ and concentrated under reduced pressure. The resulting residue was dissolved in a mixture of petroleum ether/EtOAc and filtered over a short plug of silica. The solvent was removed under reduced pressure and the resulting crude triflate was used without further purification. A 2 M solution of lithium diisopropyl amide (3.8 mL, 7.5 mmol) was cooled to -78 °C. Diethyl (difluoromethyl) phosphonate (1.2 mL, 7.5 mmol) was dissolved in THF (2 mL) and cooled to -78°C. The LDA solution was slowly added to the phosphonate solution. The resulting solution was stirred for 10 min at -78 °C. The crude triflate (1.2 g, 1.5 mmol) was dissolved in THF (3 mL) and cooled to -78 °C. To this solution the solution of the deprotonated phosphonate was slowly added. The reaction was stopped by the addition of concentrated aqueous NH_4Cl-solution. The resulting slurry was allowed to warm to room temperature and was extracted with Et_2O (3x 25 mL). The combined organic layers were washed with brine (50 mL), dried over $MgSO_4$ and concentrated under reduced pressure. The residue was purified by FC (petroleum ether/EtOAc = 5:1) to give **6** (0.8 g, 1.0 mmol, 68 % o2s) as a colorless oil.

R_f = 0.37 (petroleum ether/EtOAc = 5:1); ^{1}H NMR ($CDCl_3$, 500 MHz) δ [ppm] = 7.55 – 7.09 (m, 25H), 5.06 (d, 1H *J* = 11.1 Hz, O-C<u>H</u>HPh), 4.96 (d, 1H, *J* = 11.7 Hz, O-C<u>H</u>HPh), 4.80 (m, 2H, O-C<u>H</u>HPh), 4.72 – 4.63 (m, 2H, O-C<u>H</u>HPh, H-1), 4.51 (d, *J* = 11.1 Hz, 1H, O-C<u>H</u>HPh), 4.24 (m, 4H, PC<u>H$_2$</u>), 3.91 (d, 2H, *J* = 13.7 Hz, N-C<u>H$_2$</u>Ph,), 3.83 – 3.65 (m, 4H, N-C<u>H$_2$</u>Ph, H-3, H-5), 3.24 (dd, 1H, *J* = 9.8, 8.3 Hz, H-4), 3.00 (dd, 1H, *J* = 10.1, 8.3 Hz, H-2), 2.62 – 2.41 (m, 1H, H-6), 2.16 (m, 1H, H-6), 1.34 (m, 6 H, CH_3); ^{13}C NMR ($CDCl_3$, 126 MHz) δ [ppm] = 139.8, 139.0, 138.0, 137.5, 129.1, 128.4, 128.3, 128.1, 128.0, 127.5, 127.4, 126.9, 100.1 (C-1), 82.1 (C-4), 81.5 (C-3), 75.0 (O-<u>C</u>H_2Ph), 74.7(O-<u>C</u>H_2Ph), 70.3(O-<u>C</u>H_2Ph), 69.0 (C-5), 64.7(2x PCH_2), 63.3 (C-2), 54.7 (2 x N-<u>C</u>H_2Ph), 35.7 (C-6), 16.5(CH_3); ^{19}F NMR ($CDCl_3$, 377 MHz) δ [ppm] = -108.9 (dddd,

J = 299.0, 107.6, 29.7, 10.7 Hz), -111.9 (dddd, J = 299.0, 107.6, 26.1, 12.2 Hz); ^{31}P NMR ($CDCl_3$, 202 MHz,) δ [ppm] = 7.28 (t, J = 107.6 Hz); HRMS (ESI) m/z calcd for $C_{46}H_{52}F_2NO_7P$: 800.3522 [M+H$^+$], found: 800.3516.

(*R*)-1-(Diethoxyphosphoryl)-2-((2*R*,3*R*,4*R*,5*R*,6*R*)-3,4,6-tris(benzyloxy)-5-(dibenzylamino)tetrahydro-2*H*-pyran-2-yl)ethyl (*S*)-3,3,3-trifluoro-2-methoxy-2-phenylpropanoate ((*R*)-8-(*S*)-MTPA ester)

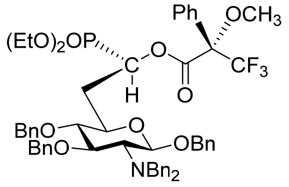

Alcohol (*R*)-**8** (20.0 mg. 25.6 µmol), DMAP (4.7 mg, 38.5 µmol), EDC*HCl (11.9 mg, 76.9 µmol) and (*S*)-MTPA acid (18.0 mg, 76.9 µmol) were dissolved in dry DCM (1 mL) and stirred at room temperature for two hours. The reaction was stopped by addition of aq. 0.2 M HCl solution (8 mL) and extracted with DCM (3x10 mL). The mixture was dried over $MgSO_4$ and the solvents were evaporated under reduced pressure. The crude product was purified using HPLC (Kinetex® C-18, 21.2 mm, 30 mL/min, 100% MeCN in 30 min) and isolated as a colorless oil (14.4 mg, 56%).

R_f = 0.35 (EtOAc/petroleum ether, 1:2); ^{1}H NMR (500 MHz, $CDCl_3$) δ [ppm] = 7.60-7.00 (m, 25H), 5.74 (dt, 1H, J = 8.5, 6.5 Hz, H-7), 5.29 (s, 1H), 4.99 (d, 1H, J = 11.2 Hz, CH_2-OBn), 4.95 (d, 1H, J = 11.7 Hz, CH_2-OBn), 4.77 (d, 1H, J = 11.2 Hz, CH_2-OBn), 4.69 (d, 1H, J = 11.2 Hz, CH_2-OBn), 4.65 (d, 1H, J = 4.6 Hz, CH2-OBn), 4.63 (d, 1H, J = 8.1 Hz, H1), 4.46 (d, 1H, J = 11.2 Hz, CH_2-OBn), 4.18-4.02 (m, 4H, CH_2-OEt), 3.91 (d, 2H, J = 13.8 Hz, CH_2-NBn), 3.75 (d, 2H, J = 13.8 Hz, CH2-NBn), 3.68 (dd, 1H, J = 10.0 Hz, J = 8.4 Hz, H-3), 3.58 (s, 3H, OMe), 3.54 (1H, m, H-5), 3.14 (dd, 1H, J = 9.7, 8.4 Hz, H-4), 2.93 (dd, 1H, J = 10.0, 8.1 Hz, H-2), 2.42 (m, 1H, H-6), 1.84- 1.72 (m, 1H, H-6), 1.25 (td, 6H, J = 7.0, 1.4 Hz); ^{13}C NMR (101 MHz, $CDCl_3$) δ [ppm] = 101.1 (C-1), 83.4 (C-4), 81.0 (C-3), 71.0 (C-5), 70.9 (Bn), 74.9 (Bn), 74.5 (Bn), 66.8 (C-7), 63.1 (C-2), 62.7 (C-10), 62.7 (C-8), 55.8 (Methoxy-Me), 54.6 (NBn), 32.6 (C-6), 16.5 (Me-OEt), 16.3 (Me-OEt); ^{19}F NMR (471 MHz, $CDCl_3$) δ [ppm] = - 71.28; ^{31}P NMR (162 MHz, $CDCl_3$) δ [ppm] = 19.29; HPLC: R_t = 4.4 min (Kinetex® C-18, 21.2 mm, 30 mL/min, 100% MeCN in 30 min).

(*R*)-1-(Diethoxyphosphoryl)-2-((2*R*,3*R*,4*R*,5*R*,6*R*)-3,4,6-tris(benzyloxy)-5-(dibenzylamino)tetrahydro-2*H*-pyran-2-yl)ethyl (*R*)-3,3,3-trifluoro-2-methoxy-2-phenylpropanoate ((*R*)-8-(*R*)-MTPA ester)

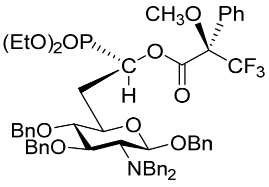

Alcohol (*R*)-**8** (20.0 mg. 25.6 µmol), DMAP (4.7 mg, 38.5 µmol, 1.5 eq.), EDC*HCl (11.9 mg, 76.9 µmol) and (*R*)-MTPA acid (18.0 mg, 76.9 µmol) were dissolved in dry DCM (1 mL) and stirred at room temperature for two hours. The reaction was stopped by addition of aq. 0.2 M HCl solution (8 mL) and extracted with DCM (3x10 mL). The mixture was dried over $MgSO_4$ and the solvents were evaporated under reduced pressure. The crude product was purified using HPLC (Kinetex® C-18, 21.2 mm, 30 mL/min, 100% MeCN in 30 min) and isolated as a colorless oil (12.4 mg, 47 %).

R_f = 0.30 (EtOAc/petroleum ether, 1:2); ^{1}H NMR (400 MHz, $CDCl_3$,) δ [ppm] = 7.60-7.12 (m, 25H), 5.72 (dt, 1H, *J* = 8.3, 6.1 Hz, H-7), 5.03 (d, 1H, *J* = 11.2 Hz, CH_2-OBn), 4.95 (d, 1H, *J* = 11.7 Hz, CH_2-OBn), 4.80 (d, 1H, *J* = 11.2 Hz, CH_2-OBn), 4.76 (d, 1H, *J* = 11.2 Hz, CH2-OBn), 4.72 (d, 1H, *J* = 8.2 Hz, H-1), 4.66 (d, 1H, *J* = 11.7 Hz, CH2-OBn), 4.51 (d, 1H, *J* = 11.3 Hz, CH2-OBn), 4.08 (m, 4H, NBn), 4.02-3.89 (m, 4H, CH2-OEt), 3.73 (d, 1H, *J* = 13.9 Hz, H-3), 3.64 (d, 1H, *J* = 14.0 Hz, H-5), 3.49 (s, 3H, OMe), 3.23 (dd, 1H, *J* = 9.5 Hz, 8.3 Hz, H-4), 2.96 (dd, 1H, *J* = 10.1 Hz, 8.2 Hz, H-2), 2.56-2.43 (m, 1H, H-6), 1.90-1.72 (m, 1H, H-6), 1.26 (t, 3H, *J* = 7.1 Hz, Me-OEt), 1.19 (t, 3H, *J* = 7.1 Hz, Me-OEt); ^{13}C NMR (101 MHz, $CDCl_3$) δ [ppm] = 101.2 (C-1), 83.0 (C-4), 81.0 (C-3) 74.7 (OBn), 74.4 (OBn), 71.2 (OBn), 71.3 (C-5), 70.6 (OBn), 66.4 (C-7), 63.1 (CH_2-OEt), 62.8 (C-2), 54.7 (NBn), 33.0 (C-6), 16.7 (Me-OEt); ^{19}F NMR (377 MHz, $CDCl_3$,) δ [ppm] = -71.53; ^{31}P NMR (162 MHz, $CDCl_3$) δ [ppm] = 18.78; HPLC: R_t = 4.5 min (Kinetex C-18, 30 mL/min, 100% MeCN in 30 min).

(*S*)-1-(Diethoxyphosphoryl)-2-((2*R*,3*R*,4*R*,5*R*,6*R*)-3,4,6-tris(benzyloxy)-5-(dibenzylamino)tetrahydro-2*H*-pyran-2-yl)ethyl (*S*)-3,3,3-trifluoro-2-methoxy-2-phenylpropanoate ((*S*)-8-(*S*)-MTPA ester)

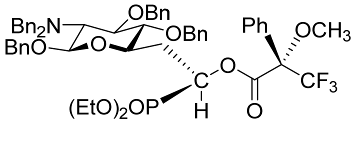

Alcohol (*S*)-**8** (20.0 mg, 25.6 µmol), DMAP (4.7 mg, 38.5 µmol), EDC*HCl (11.9 mg, 76.9 µmol) and (*S*)-MTPA acid (18.0 mg, 76.9 µmol) were dissolved in dry DCM (1 mL) and stirred at room temperature for two hours. The reaction was stopped by addition of aq. 0.2 M HCl solution (8 mL) and extracted with DCM (3x10 mL). The mixture was dried over $MgSO_4$ and the solvents were evaporated under

reduced pressure. The crude product was purified using HPLC (Kinetex® C-18, 21.2 mm, 30 mL/min, 100% MeCN in 30 min) and isolated as a colorless oil (8.7 mg, 34 %).

^{1}H NMR (400 MHz, $CDCl_3$,) δ [ppm] = 7.68 – 7.16 (m, 30H), 5.83 (dd, 1H, *J* = 12.0, 7.9 Hz, H-7), 5.01 (d, 1H, *J* = 11.1 Hz, O-CHHPh), 4.96 (d, 1H, *J* = 11.9 Hz, O-CHHPh), 4.79 (m, 2H, O-CHHPh), 4.55 (t, 2H, *J* = 11.6 Hz, O-CHHPh), 4.48 (d, *J* = 8.3 Hz, 1H, H-1), 4.11 – 3.93 (m, 3H,), 3.87 (d, *J* = 13.7 Hz, 2H, N-CH_2Ph), 3.73 (d, *J* = 13.7 Hz, 2H, N-CH_2Ph), 3.63 (dd, 1H, *J* = 10.1, 8.3 Hz, H-5), 3.53 (s, 3H; OMe), 3.23 (pt, 1H, *J* = 8.9 Hz, H-4), 3.09 (pt, 1H *J* = 10.1 Hz, H-3), 2.95 (dd, 1H, *J* = 10.1, 8.3 Hz, H-2), 2.41 (m, 1H, 1x H-6), 1.94 (m, 1H, 1x H-6), 1.29 – 1.20 (m, 6H, CH_3); ^{31}P NMR ($CDCl_3$, 162 MHz) δ [ppm] = 19.36; ^{19}F NMR ($CDCl_3$, 377 MHz) δ [ppm] = -71.3.

(*S*)-1-(Diethoxyphosphoryl)-2-((2*R*,3*R*,4*R*,5*R*,6*R*)-3,4,6-tris(benzyloxy)-5-(dibenzylamino)tetrahydro-2*H*-pyran-2-yl)ethyl (*R*)-3,3,3-trifluoro-2-methoxy-2-phenylpropanoate ((*S*)-8-(*R*)-MTPA ester)

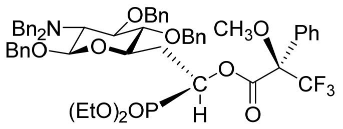

Alcohol (*S*)-**8** (20.0 mg, 25.6 µmol), DMAP (4.7 mg, 38.5 µmol, 1.5 eq.), EDC*HCl (11.9 mg, 76.9 µmol) and (*R*)-MTPA acid (18.0 mg, 76.9 µmol) were dissolved in dry DCM (1 mL) and stirred at room temperature for two hours. The reaction was stopped by addition of aq. 0.2 M HCl solution (8 mL) and extracted with DCM (3x10 mL). The mixture was dried over $MgSO_4$ and the solvents were evaporated under reduced pressure. The crude product was purified using HPLC (Kinetex® C-18, 21.2 mm, 30 mL/min, 100% MeCN in 30 min) and isolated as a colorless oil (9.3 mg, 36 %).

^{1}H NMR (400 MHz, $CDCl_3$,) δ [ppm] = 7.68 – 7.10 (m, 30H), 5.82 (ddd, 1H, *J* = 12.4, 7.4, 1.7 Hz, H-7), 5.00 (d, 1H, *J* = 11.9 Hz, O-CHHPh), 4.96 (d, 1H, *J* = 11.2 Hz, O-CHHPh), 4.76 (d, 1H, *J* = 11.1 Hz, O-CHHPh), 4.68 (dd, 2H, *J* = 13.6, 11.6 Hz, O-CHHPh), 4.50 – 4.45 (m, 2H O-CHHP, H-1), 4.09 (m, 4H, CH_2), 3.88 (d, 2H, *J* = 13.7 Hz, N-CH_2Ph), 3.81 – 3.72 (m, 2H, N-CH_2Ph,), 3.60 (s, 3H, OMe), 3.54 (dd, 1H, *J* = 10.1, 8.4 Hz, H-5), 3.16 (pt, *J* = 9.0 Hz, 1H, H-4), 2.93 (dd, 1H, *J* = 10.1, 8.2 Hz, H-2), 2.88 (m, 1H, H-3), 2.30 (m 1H, 1x H6), 1.86 (m, 1H, 1xH6), 1.36 – 1.15 (m, 6H, CH_3); ^{31}P NMR ($CDCl_3$, 162 MHz) δ [ppm] = 19.62; ^{19}F NMR ($CDCl_3$, 377 MHz) δ [ppm] = -70.81.

Diethyl ((*R*)-1-fluoro-2-((2*R*,3*R*,4*R*,5*R*,6*R*)-3,4,6-tris(benzyloxy)-5-(dibenzylamino)tetrahydro-2*H*-pyran-2-yl)ethyl)phosphonate ((*R*)-9)

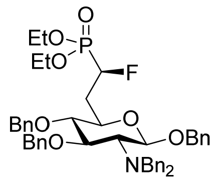

(*R*)-**9** was obtained from (*S*)-**9** by base-catalyzed isomerization. (*S*)-**9** (180 mg, 230 µmol) was dissolved in THF (3 mL) and cooled to -78 °C. A freshly prepared 2 M solution of lithium diisopropylamide (1.73 mL, 3.45 mmol) in THF was cooled to -78 °C and added slowly to the solution of compound (*S*)-**9**. After stirring for 1 h at -78 °C a solution of AcOH (395 µL, 6.1 mmol) in 1 mL THF was slowly added and the resulting mixture was allowed to reach room temperature. DCM (3 mL) was added, and the organic phase was washed with water (2x 1 mL) and brine (2 mL). The organic phase was dried over $MgSO_4$ and concentrated under reduced pressure. The residue was purified by FC (petroleum ether/EtOAc = 3:1 to 1:1) to give (*R*)-**9** (76 mg, 97 µmol, 42 %) and (*S*)-**9** (82 mg, 104 µmol, 45 %) as colorless oils.

(*R*)-**9**: R_f = 0.62 (petroleum ether/EtOAc = 1:2); ^{1}H NMR ($CDCl_3$, 400 MHz) δ [ppm] = 7.55 – 7.10 (m, 25H), 5.15 – 4.96 (m, 2H, O-CHHPh, PCHF), 4.93 (d, 1H, *J* = 11.7 Hz, O-CHHPh), 4.86 – 4.74 (m, 2H, O-CHHPh), 4.69 (d, 1H, *J* = 8.3 Hz, H-1), 4.64 (d, 1H, *J* = 11.7 Hz, O-CHHPh), 4.56 (d, 1H, *J* = 10.8 Hz, O-CHHPh), 4.22 – 4.12 (m, 4H, PCH_2), 3.93 (d, 2H, *J* = 13.7 Hz, N-CH_2Ph), 3.79 (d, 2H, *J* = 13.8 Hz, N-CH_2Ph), 3.72 (dd, 1H, *J* = 10.1, 8.3 Hz, H-3), 3.60 – 3.45 (m, 1H, H-5), 3.37 (pt, 1H, *J* = 9.0 Hz, H-4), 2.99 (dd, 1H, *J* = 10.1, 8.3 Hz, H-2), 2.49 – 2.28 (m, 1H, H-6), 2.31 – 2.12 (m, 1H, H-6), 1.33 (m, 6H, 2x CH_3); ^{13}C NMR ($CDCl_3$,101 MHz) δ [ppm] = 139.8, 139.0, 138.2, 137.6, 129.0, 128.7, 128.6, 128.5, 128.4, 128.3, 128.1, 128.0, 127.9, 127.5, 127.4, 126.9, 100.9 (C-1), 83.0 (C-4), 81.5 (C-3), 75.0 (O-CH_2Ph), 74.6 (O-CH_2Ph), 70.8 (O-CH_2Ph), 63.4 (2x PCH_2), 54.9 (2 x N-CH_2Ph), 32.6 (C-6), 16.7 (CH_3); ^{31}P NMR ($CDCl_3$, 162 MHz) δ [ppm] = 17.5 (d, *J* = 73.2 Hz); ^{19}F NMR ($CDCl_3$, 377 MHz) δ [ppm] =-205.9 (d, *J* = 73.1 Hz); HRMS (ESI) *m/z* calcd for $C_{46}H_{53}FNO_7P$: 782.3616 [M+H^+], found: 782.3613.

Diethyl ((*S*)-1-fluoro-2-((2*R*,3*R*,4*R*,5*R*,6*R*)-3,4,6-tris(benzyloxy)-5-(dibenzylamino)tetrahydro-2*H*-pyran-2-yl)ethyl)phosphonate ((*S*)-9)

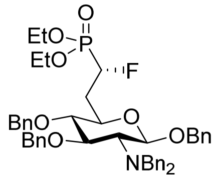

Diethylaminosulfur trifluoride (88.0 µL, 667 µmol) was dissolved with DCM (7 mL) and cooled to -78 °C. The alcohol (*R*)-**8** (260 mg, 333 µmol) was dissolved in DCM (3 mL) and cooled to -78 °C. The solution

of the alcohol was slowly added to the DAST solution. The reaction was allowed to warm to room temperature and was stirred for 1.5 h. The reaction was stopped by addition of a saturated aqueous $NaHCO_3$-solution (10 mL). The aqueous layer was extracted with DCM (3x10 mL). The combined organic layers were washed with brine (50 mL), dried over $MgSO_4$ and concentrated under reduced pressure. The residue was purified by FC (petroleum ether/EtOAc = 2:1 to 1:1) to give (*S*)-**9** (152 mg, 194 µmol, 58 %) as a colorless oil.

R_f = 0.54 (petroleum ether/EtOAc = 1:2); ^{1}H NMR ($CDCl_3$, 600 MHz) δ [ppm] = 7.55 – 7.12 (m, 25H), 5.10 – 4.95 (m, 2H, O-CHHPh, PCHF), 4.94 (d, 1H, *J* = 11.7 Hz, O-CHHPh), 4.85 (d, 1H, *J* = 11.1 Hz, O-CHHPh), 4.80 (d, 1H, *J* = 11.0 Hz, O-CHHPh), 4.73 – 4.61 (m, 2H, H-1, O-CHHPh), 4.56 (d, 1H *J* = 10.9 Hz, O-CHHPh), 4.20 (qd, 3H, *J* = 7.1, 6.5 Hz, PCH_2), 3.95 (d, 2H, *J* = 13.6 Hz, N-CH_2Ph), 3.83 – 3.75 (m, 3H, N-CH_2Ph, H-3), 3.50 (pt, 1H, *J* = 10.0 Hz, H-5), 3.28 (pt, 1H *J* = 9.4 Hz, H-4), 3.02 (dd, 1H, *J* = 10.1, 8.2 Hz, H-2), 2.51-2.42 (m,1H, H-6), 1.90 – 1.69 (m, 1H, H-6). 1.36 (td, 6H, *J* = 7.1, 4.3 Hz, 2x CH_3); ^{13}C NMR ($CDCl_3$,126 MHz) δ [ppm] = 139.6, 138.9, 137.9, 137.1, 128.9, 128.5, 128.4, 128.3, 128.1, 128.1, 127.9, 127.4, 127.3, 126.8, 100.7 (C-1), 84.8 (C-F), 83.1 (C-4), 81.3 (C-3), 75.0 (O-CH_2Ph), 74.6 (O-CH_2Ph), 70.8(O-CH_2Ph), 69.2 (C-5), 63.3 (C-2), 63.1 (2x PCH_2), 54.7 (2 x N-CH_2Ph), 32.6 (C-6), 16.5 (CH_3); ^{31}P NMR ($CDCl_3$, 162 MHz) δ [ppm] = 18.3; ^{19}F NMR ($CDCl_3$, 377 MHz) δ [ppm] = -213.9; HRMS (ESI) *m/z* calcd for $C_{46}H_{53}FNO_7P$: 782.3616 [M+H^+], found: 782.3614.

2-Carbobenzyloxyamino-2-deoxy-5-thio-D-glucopyranose (12)

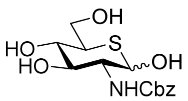

11 (23.3 mg, 0.10 mmol) was dissolved in degassed water (0.60 mL) and 1,4 dioxane (0.20 mL). Benzyl chloroformate (84 mg, 496 µmol) and sodium bicarbonate (84.5 mg, 1.01 mmol) were added, and the reaction mixture was stirred at 60 °C for 2.5 hours. The reaction mixture was allowed to cool to room temperature and the solvent was removed under vacuum. The residue was coevaporated with toluene (2 x 1 mL). The residue was purified by FC (DCM/MeOH = 7:1) to give **12** as a white solid (12.1 mg, 0.037 mmol, 37%).

R_f = 0.51 (DCM/MeOH = 5:1); ^{1}H-NMR (600 MHz, Methanol-d_4, 300 K) δ [ppm] = 7.43 – 7.26 (m, 5H, H-Ar), 5.11 (s, 2H, CH_2Ph), 4.92 (d, *J* = 2.8 Hz, 1H, α-anomer H-1), 3.90 (dd, *J* = 11.5, 3.8 Hz, 1H, H-6a), 3.86 (dd, *J* = 9.8, 2.9 Hz, 1H, H-2), 3.84 (dd, *J* = 11.4, 5.9 Hz, 1H, H-6b), 3.66 – 3.56 (m, 2H, H-3 and H-4), 3.26 (ddd, *J* = 9.9, 5.9, 3.8 Hz, 1H, H-5); ^{13}C-NMR (151 MHz, Methanol-d_4, 300 K) δ [ppm] = 158.6 (CO), 138.3 ($C_{quart.}$-Ar), 129.4 (C-Ar), 128.9 (C-Ar), 128.9 (C-Ar), 76.8 (C-4), 73.8 (C-1), 73.6 (C-3), 67.5 (CH_2Ph), 62.6 (C-6), 61.7 (C-2), 44.8 (C-1); HRMS calcd. for $C_{14}H_{19}NO_6S$ [M + Na]$^+$ *m/z*: 352.0825, found 352.0831.

2-Carbobenzyloxyamino-2-deoxy-6-*O*-diphenoxyphosphoryl-1,3,4-tri-*O*-acetyl-5-thio-D-glucopyranose (13)

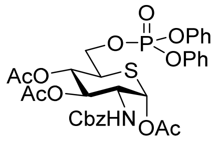

12 (846.90 mg, 2.57 mmol) was dissolved in dry pyridine (28 mL) and diphenyl phosphoryl chloride (0.80 mL, 3.86 mmol) was added dropwise at – 40 °C. The reaction mixture was stirred overnight and was allowed to warm up to room temperature. After completion of the reaction, acetic anhydride (1.5 mL, 15.89 mmol) was added, and the solution was stirred for 24 hours at room temperature. The reaction mixture was concentrated under high vacuum and the residue was purified by FC (petroleum ether/EtOAc = 1:1) to afford **13** as a white solid (0.91 g, 1.32 mmol, 52%).

R_f = 0.32 (petroleum ether/EtOAc = 1:1); ^{1}H-NMR (600 MHz, $CDCl_3$, 300 K) δ [ppm] = 7.40 – 7.29 (m, 9H, H-Ar), 7.24 – 7.16 (m, 6H, H-Ar), 5.95 (d, *J* = 3.1 Hz, 1H, α-anomer H-1), 5.34 (dd, *J* = 10.8, 9.5 Hz, 1H, H-4), 5.16 (dd, *J* = 10.9, 9.5 Hz, 1H, H-3), 5.15 – 5.09 (m, 1H, CH_2Ph-a), 5.07 – 4.97 (m, 1H, CH_2Ph-b), 4.40 – 4.28 (m, 3H, H-2 and 2x H-6), 3.57 – 3.47 (m, 1H, H-5), 2.12 (s, 2H, CH_3-1), 2.00 (s, 3H, CH_3-4), 1.91 (s, 2H, CH_3-3); ^{13}C-NMR (151 MHz, $CDCl_3$, 300 K): δ [ppm] = 171.3 ($CH_3\underline{C}OOC$-3), 169.3 ($CH_3\underline{C}OOC$-4), 168.8 ($CH_3\underline{C}OOC$-1), 155.4 (NH$\underline{C}OOCH_2$Ph), 150.42 ($C_{quart.}$-Ar), 150.37 ($C_{quart.}$-Ar), 136.1 ($C_{quart.}$-Ar), 130.0 (C-Ar), 128.7 (C-Ar), 128.5 (C-Ar), 128.4 (C-Ar), 125.7 (C-Ar), 120.23 (C-Ar), 120.20 (C-Ar), 72.8 (C-1), 71.7 (C-3 or C-4), 71.6 (C-3 or C-4), 67.4 (CH_2Ph), 66.3 (C-6), 57.1 (C-2), 41.0 (C-5), 21.2 (CH_3-1), 20.7 (CH_3-3 or CH_3-4), 20.6 (CH_3-3 or CH_3-4); ^{31}P-NMR (162 MHz, $CDCl_3$, 300 K): δ [ppm] = -12.39 (s, 1P); HRMS calcd. for $C_{32}H_{34}NO_{12}PS$ $[M + Na]^+$ *m/z*: 710.1432, found 710.1445.

7. NMR Spectra

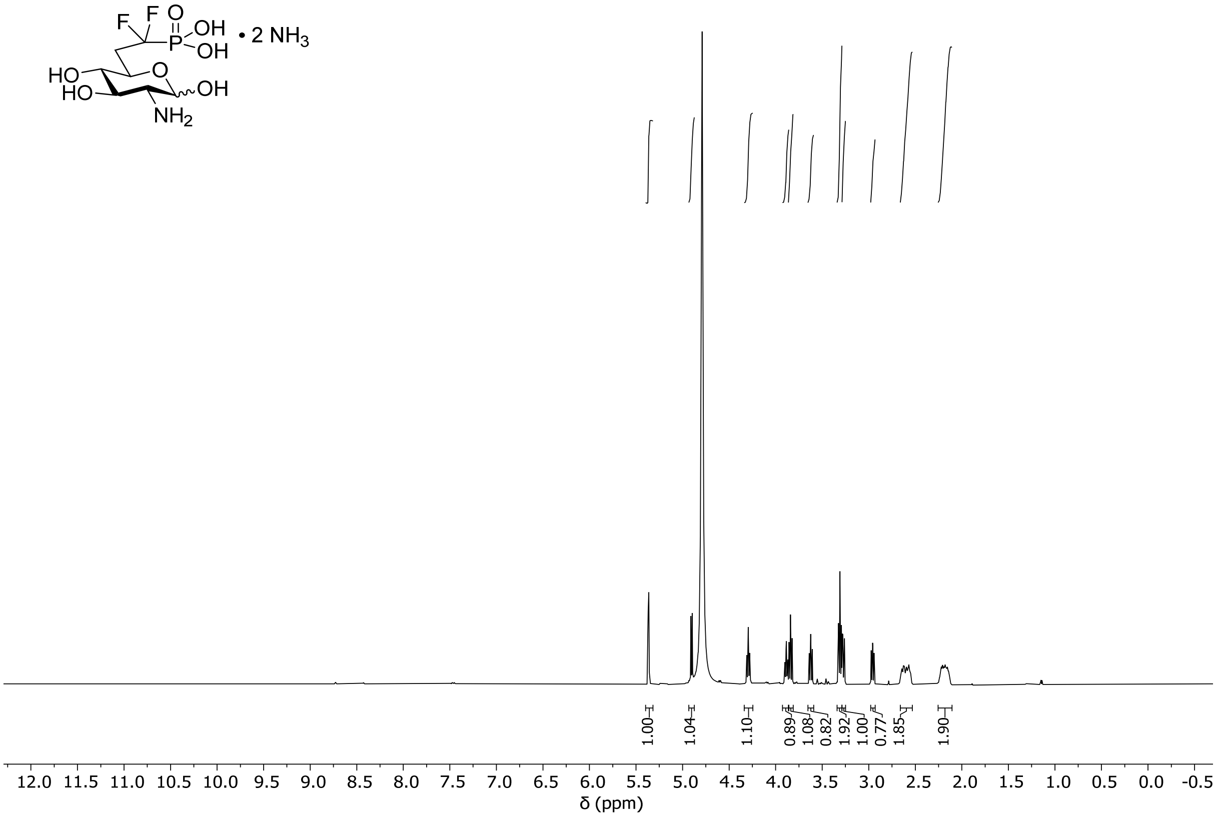

^{1}H NMR spectrum (600 MHz, D_2O) of **1** • 2 NH_3

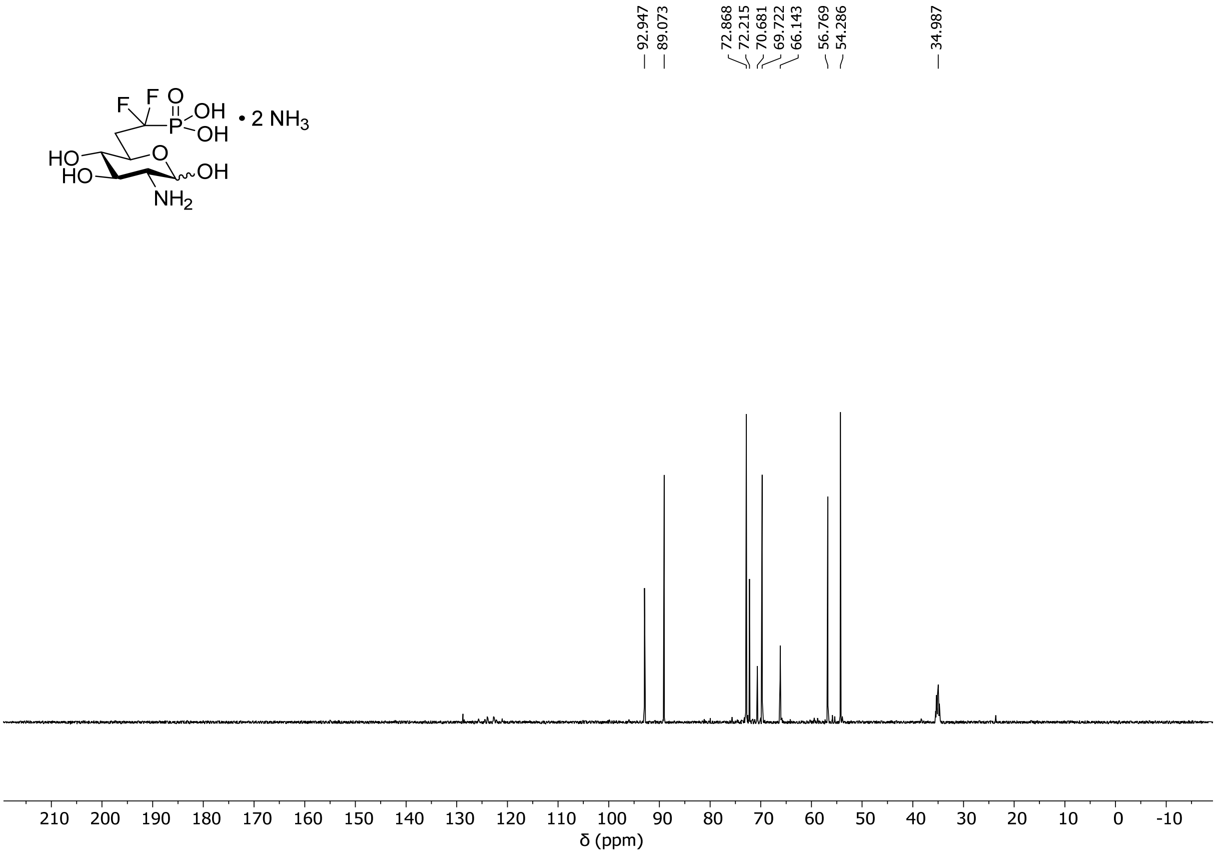

^{13}C NMR spectrum (151 MHz, D_2O) of **1** • 2 NH_3

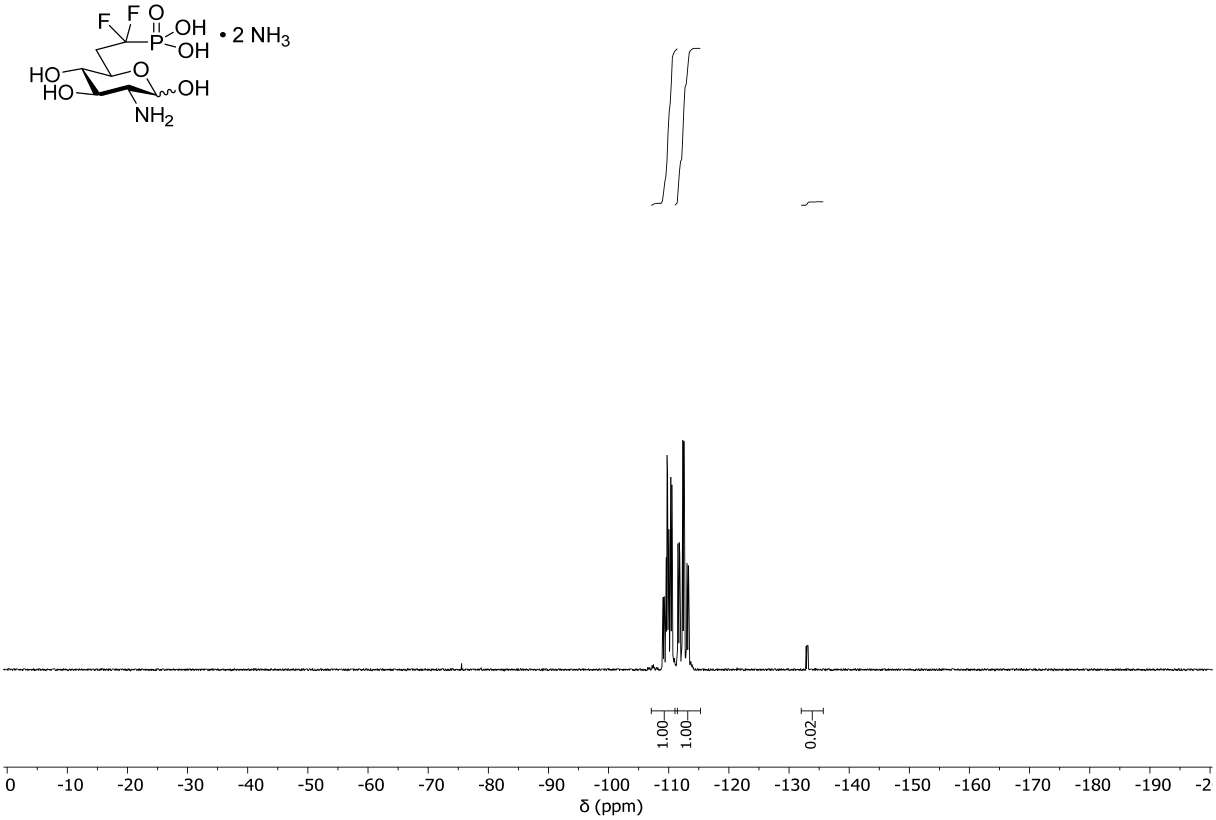

^{19}F NMR spectrum (377MHz, $CDCl_3$) of **1** • 2 NH_3

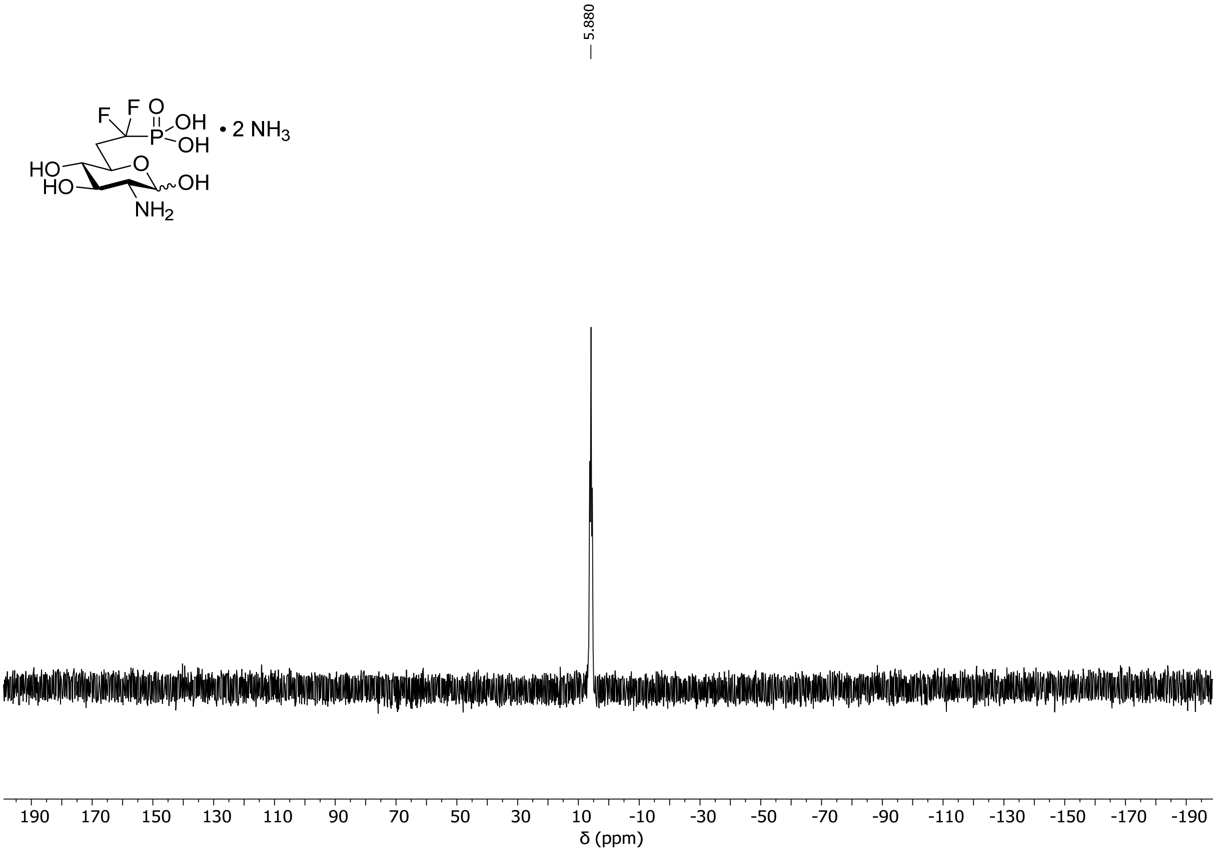

^{31}P NMR spectrum (202 MHz, D_2O) of **1** • 2 NH_3

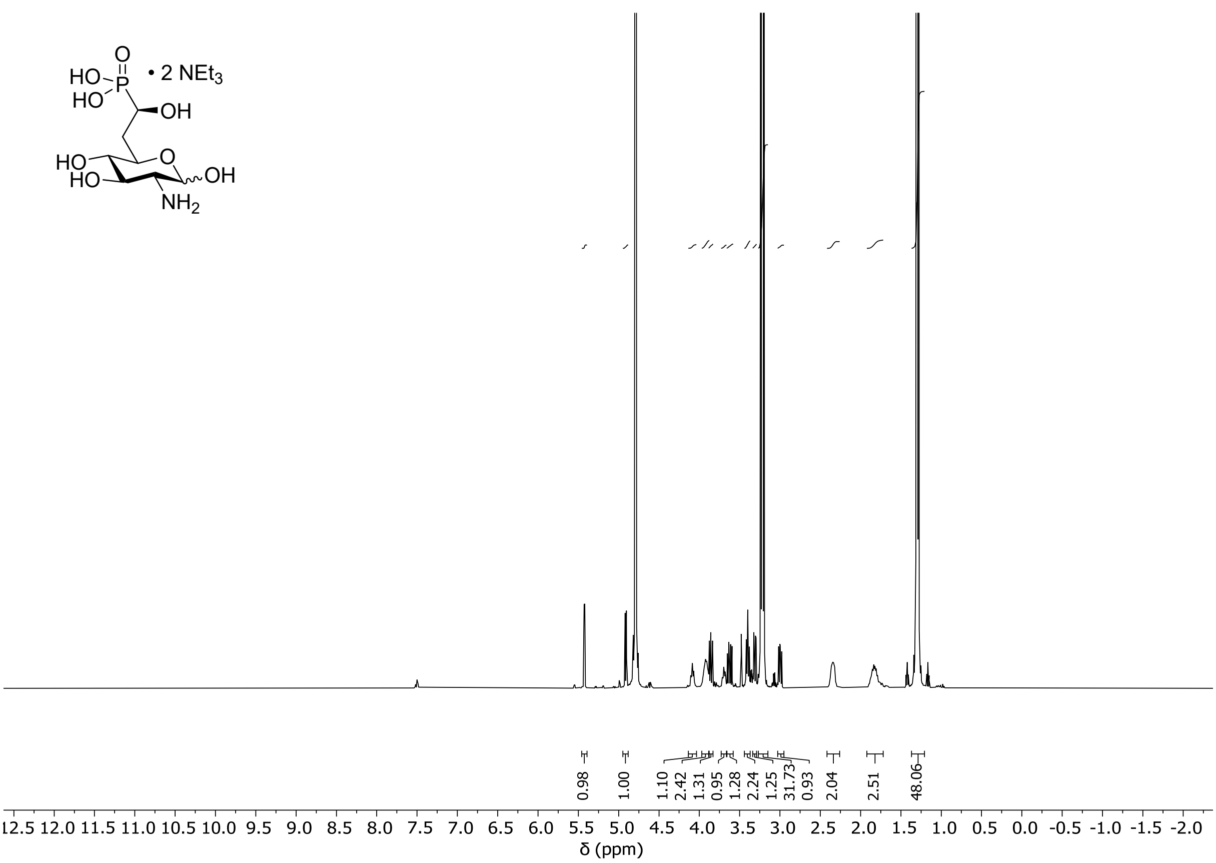

^{1}H NMR spectrum (500 MHz, D_2O) of (*R*)-**2** • 2 NEt_3

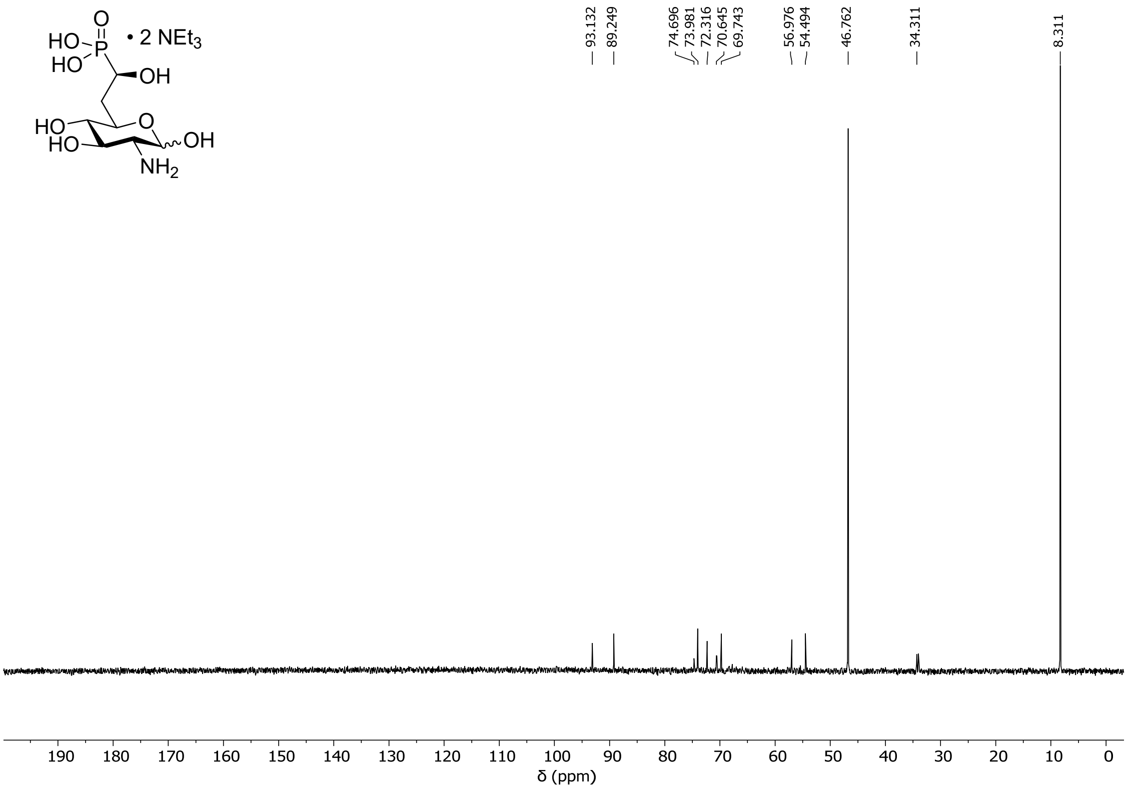

^{13}C NMR spectrum (126 MHz, D_2O) of (*R*)-**2** • 2 NEt_3

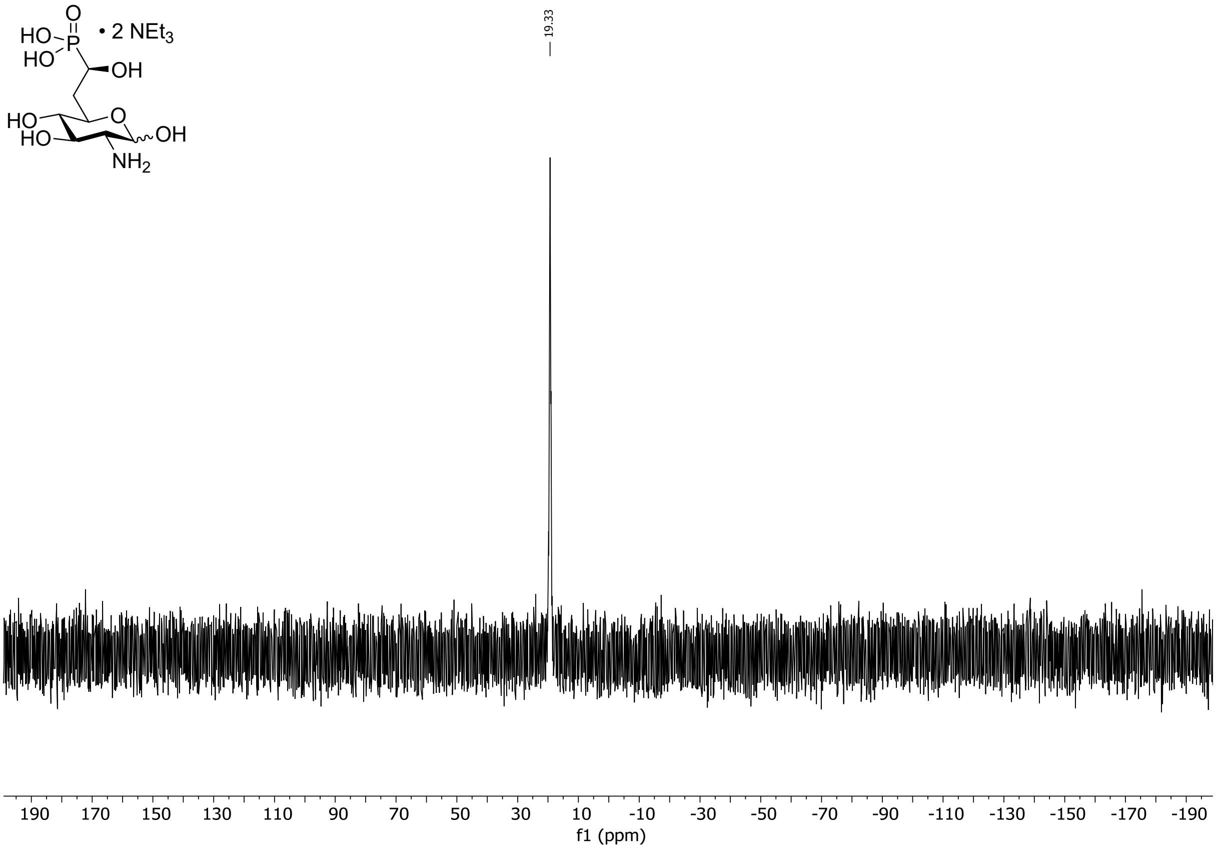

^{31}P NMR spectrum (202 MHz, D_2O) of (*R*)-**2** • 2 NEt_3

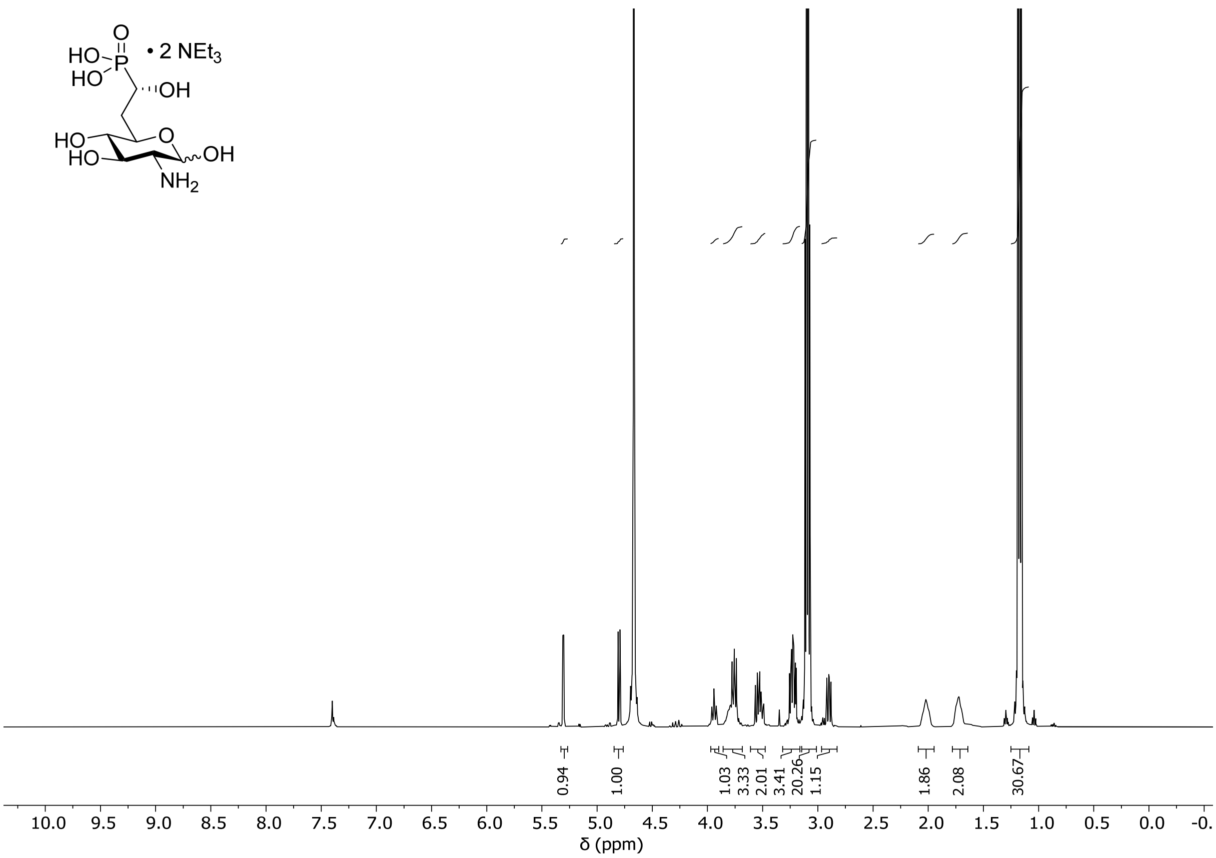

^{1}H NMR spectrum (500 MHz, D_2O) of (*S*)-**2** • 2 NEt_3

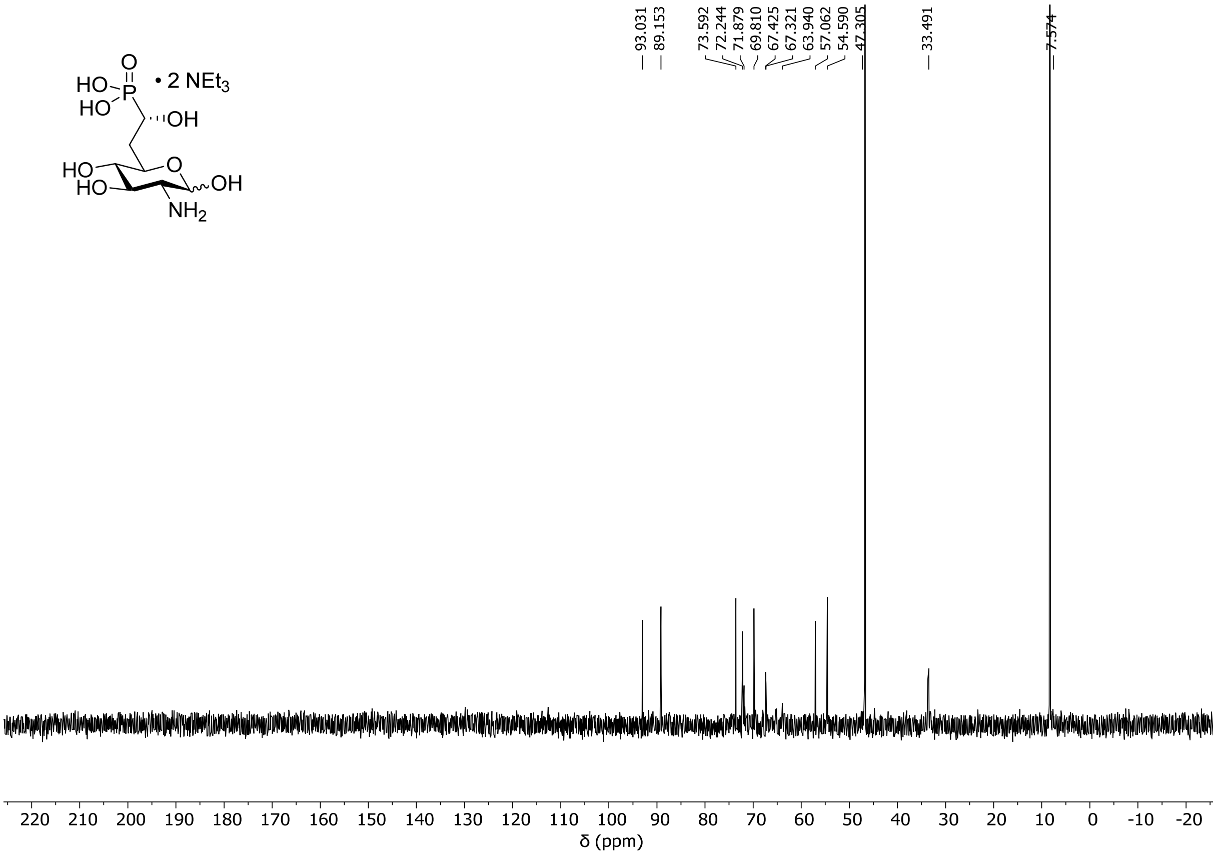

^{13}C NMR spectrum (126 MHz, D_2O) of (*S*)-**2** • 2 NEt_3

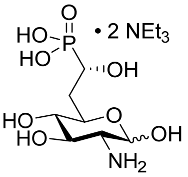

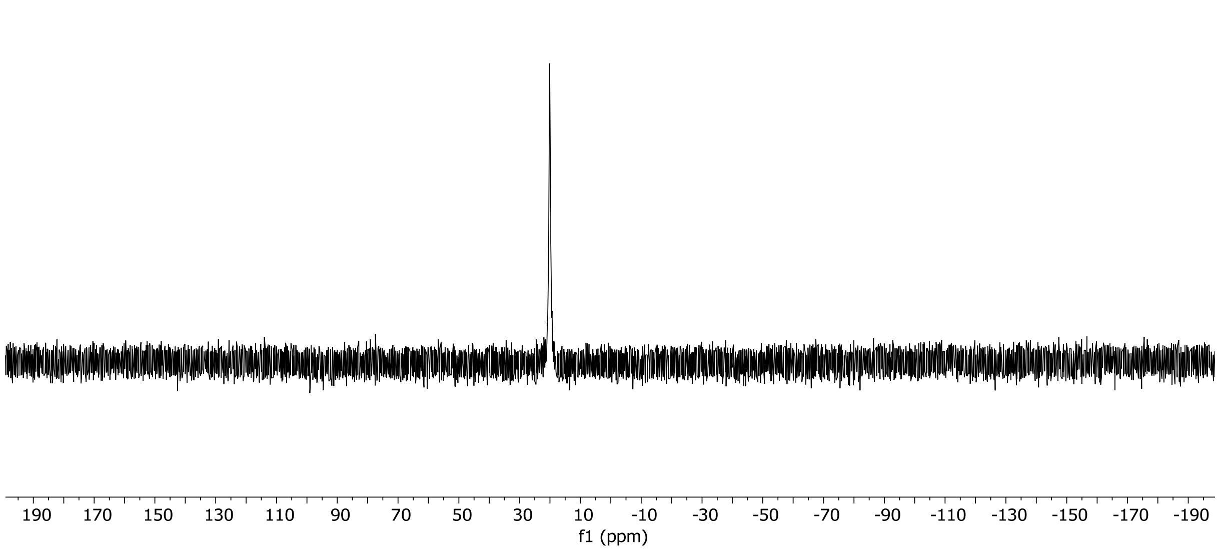

^{31}P NMR spectrum (202 MHz, D_2O) of (*S*)-**2** • 2 NEt_3

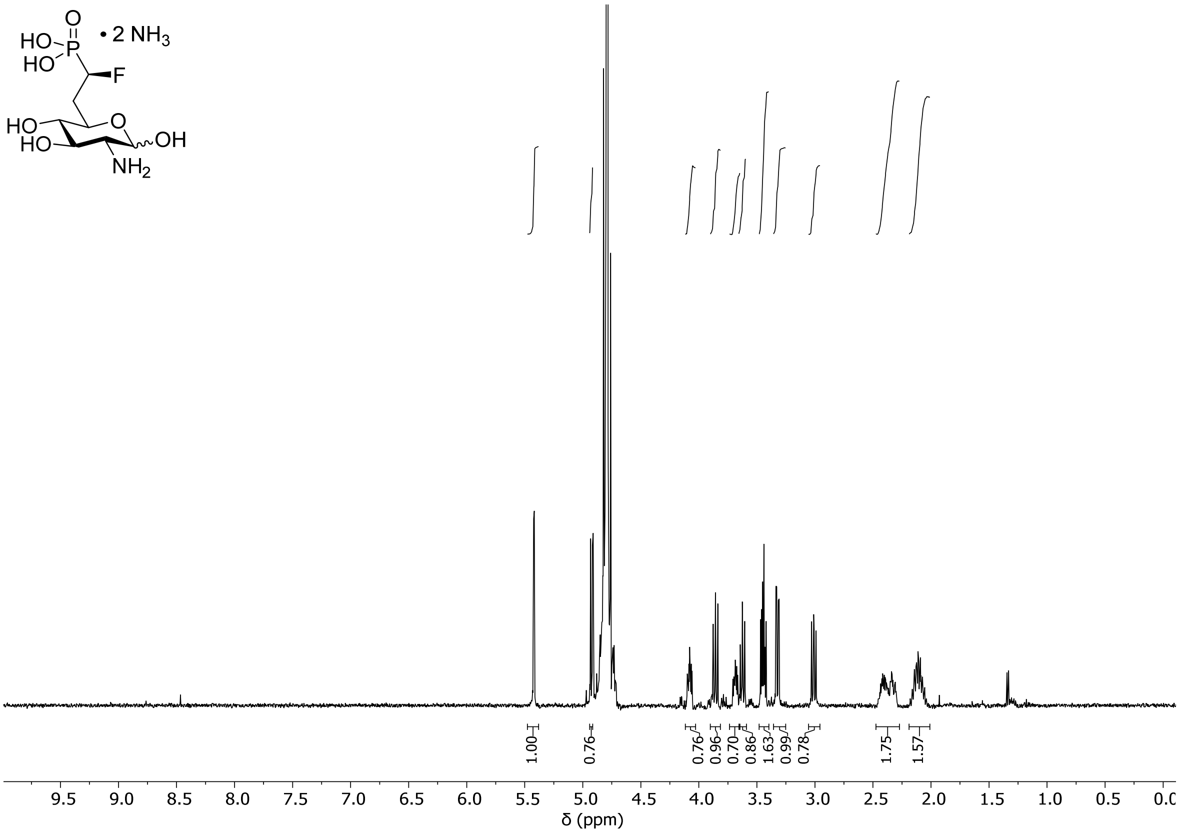

^{1}H NMR spectrum (500 MHz, D_2O) of (*R*)-**3** • 2 NH_3

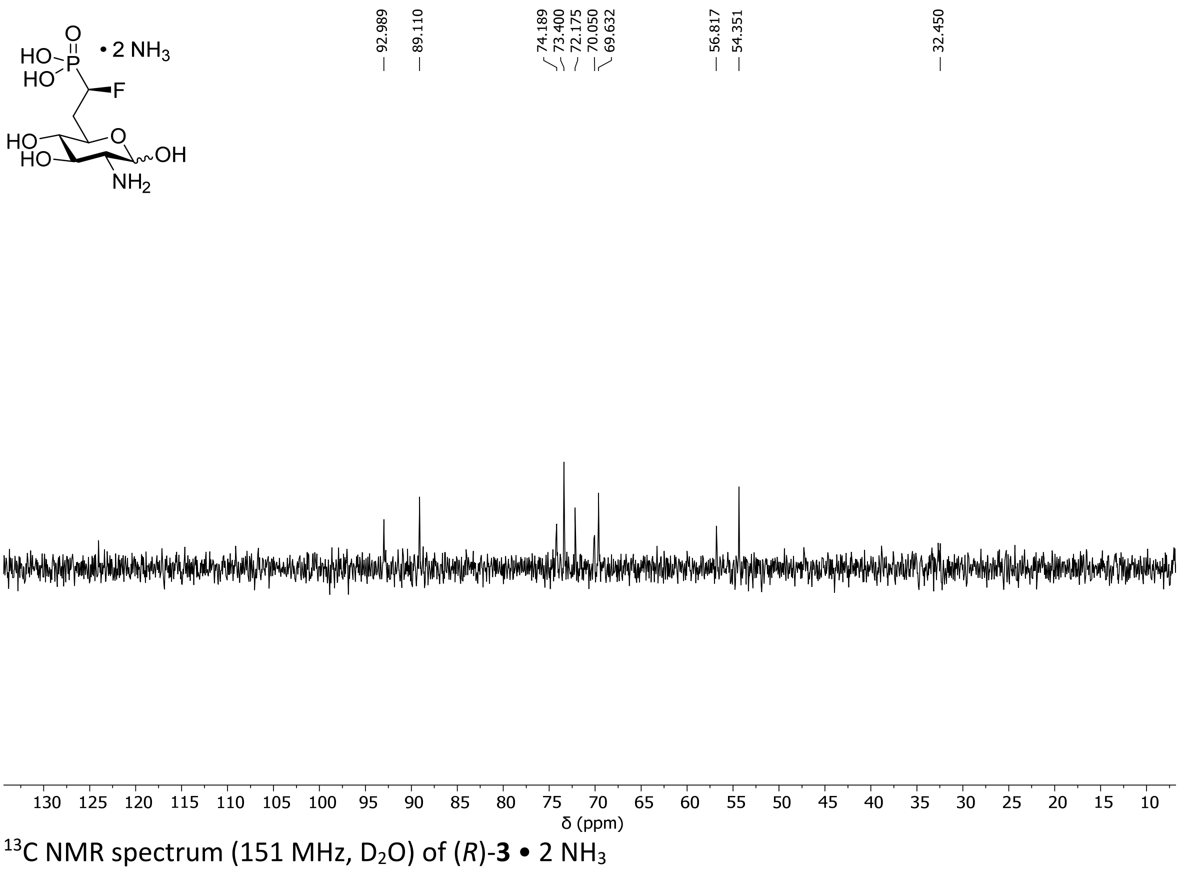

^{13}C NMR spectrum (151 MHz, D_2O) of (*R*)-**3** • 2 NH_3

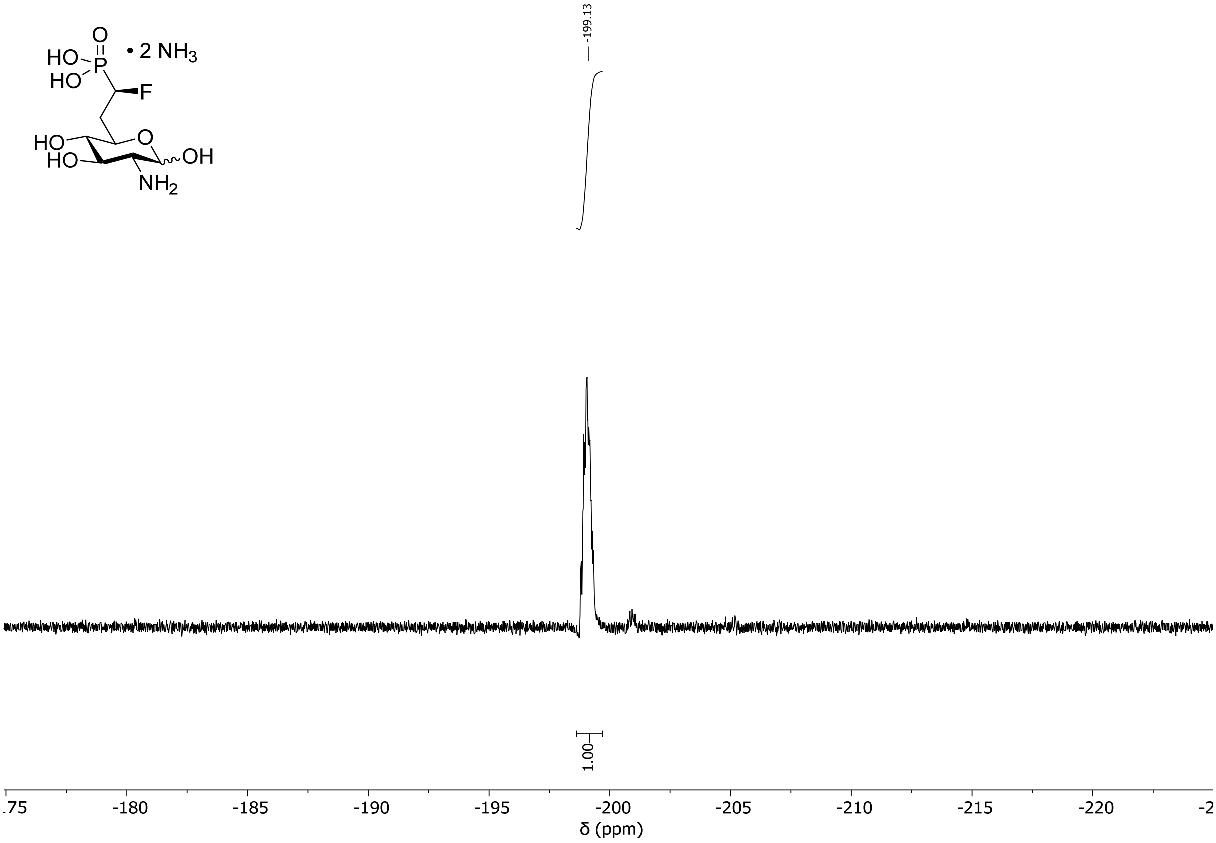

^{19}F NMR spectrum (471 MHz, D_2O) of (*R*)-**3** • 2 NH_3

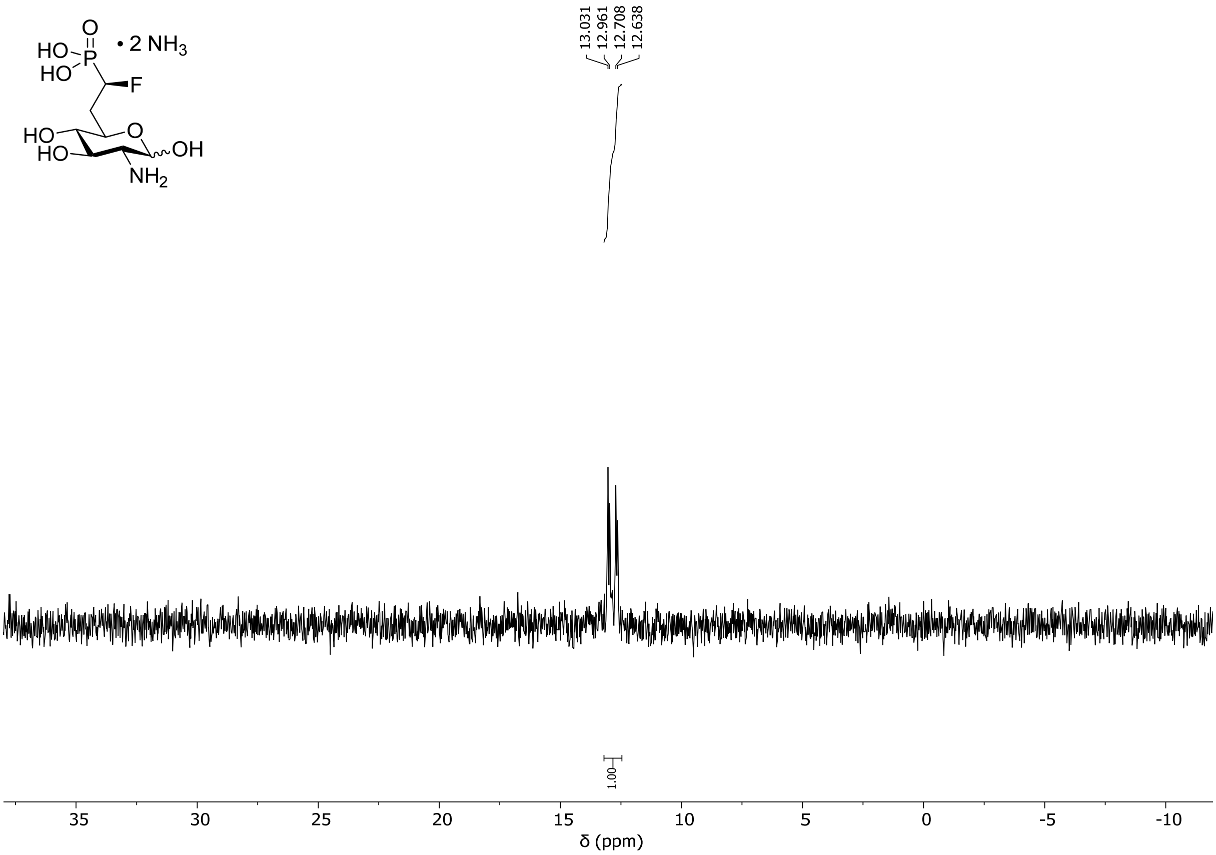

^{31}P NMR spectrum (202 MHz, D_2O) of (*R*)-**3** • 2 NH_3

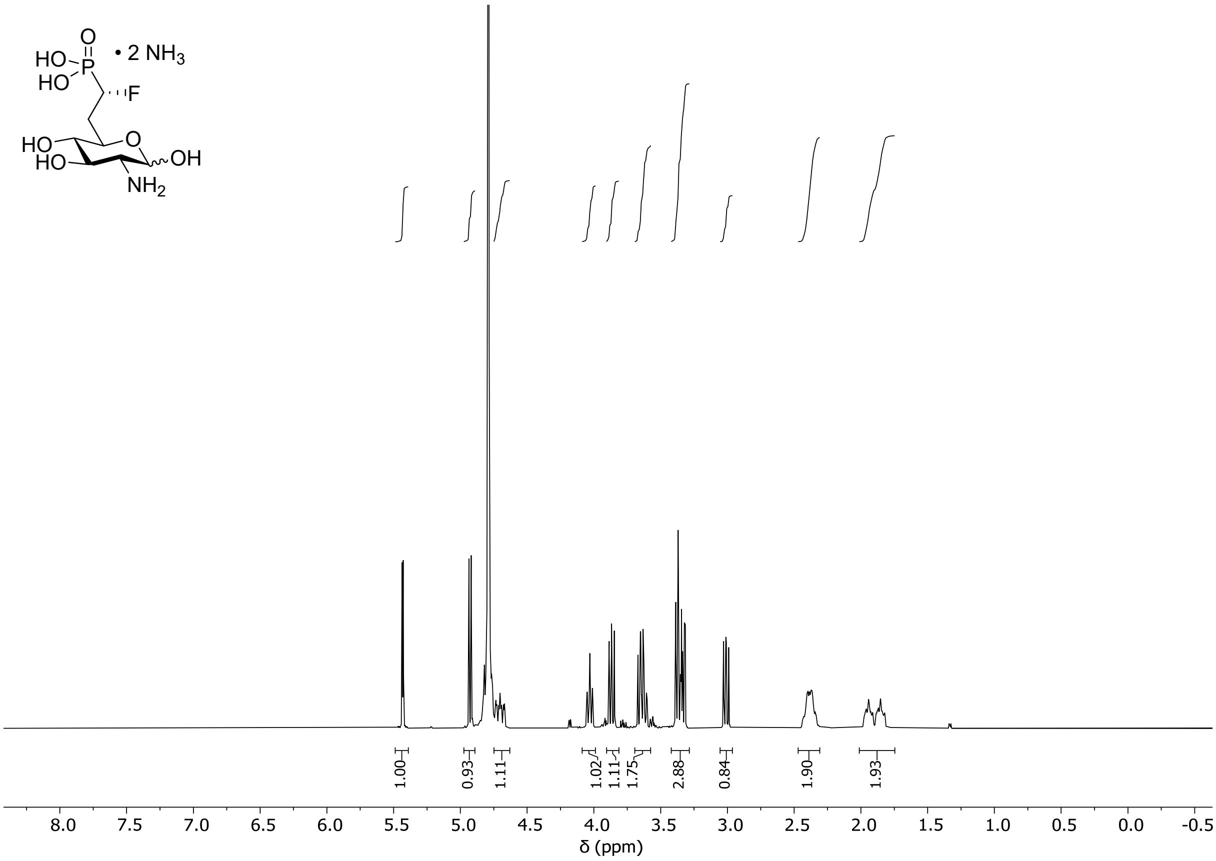

^{1}H NMR spectrum (500 MHz, D_2O) of (*S*)-**3** • 2 NH_3

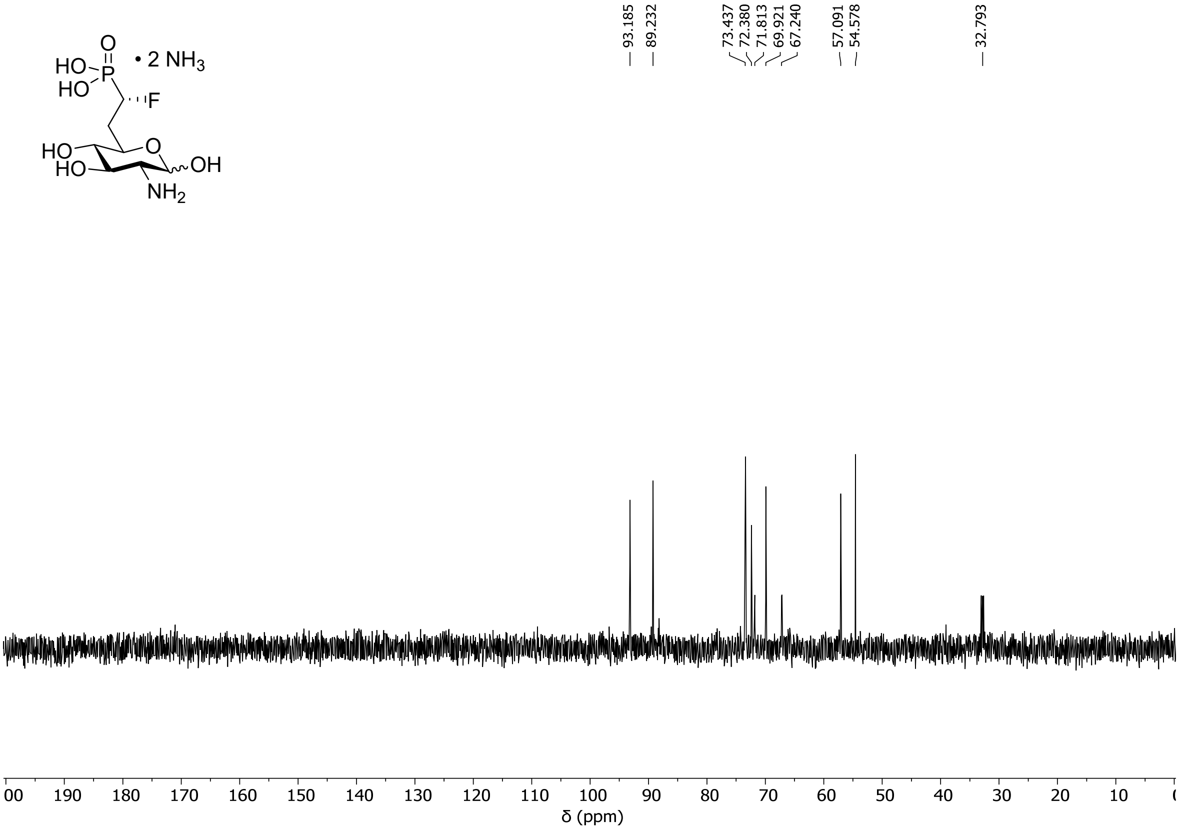

^{13}C NMR spectrum (121 MHz, D_2O) of (*S*)-**3** • 2 NH_3

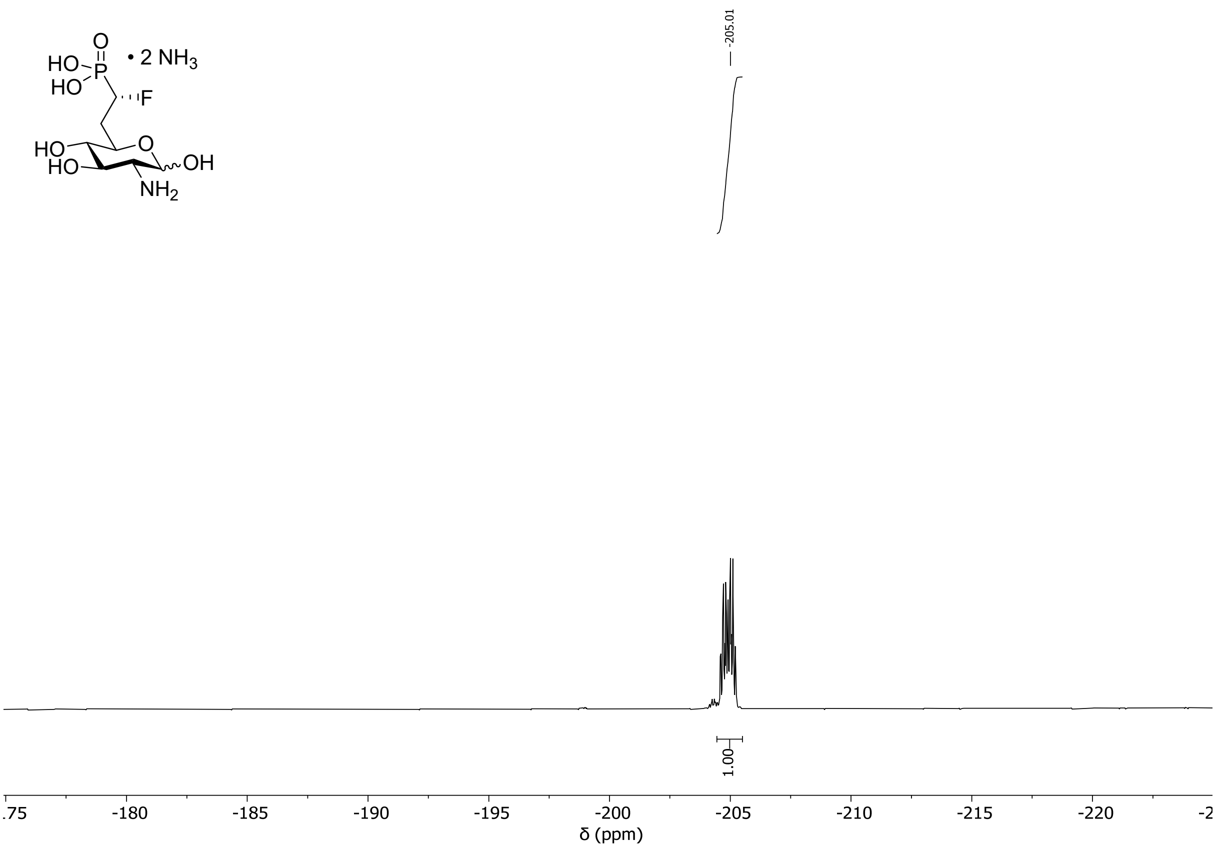

^{19}F NMR spectrum (471 MHz, D_2O) of (*S*)-**3** • 2 NH_3

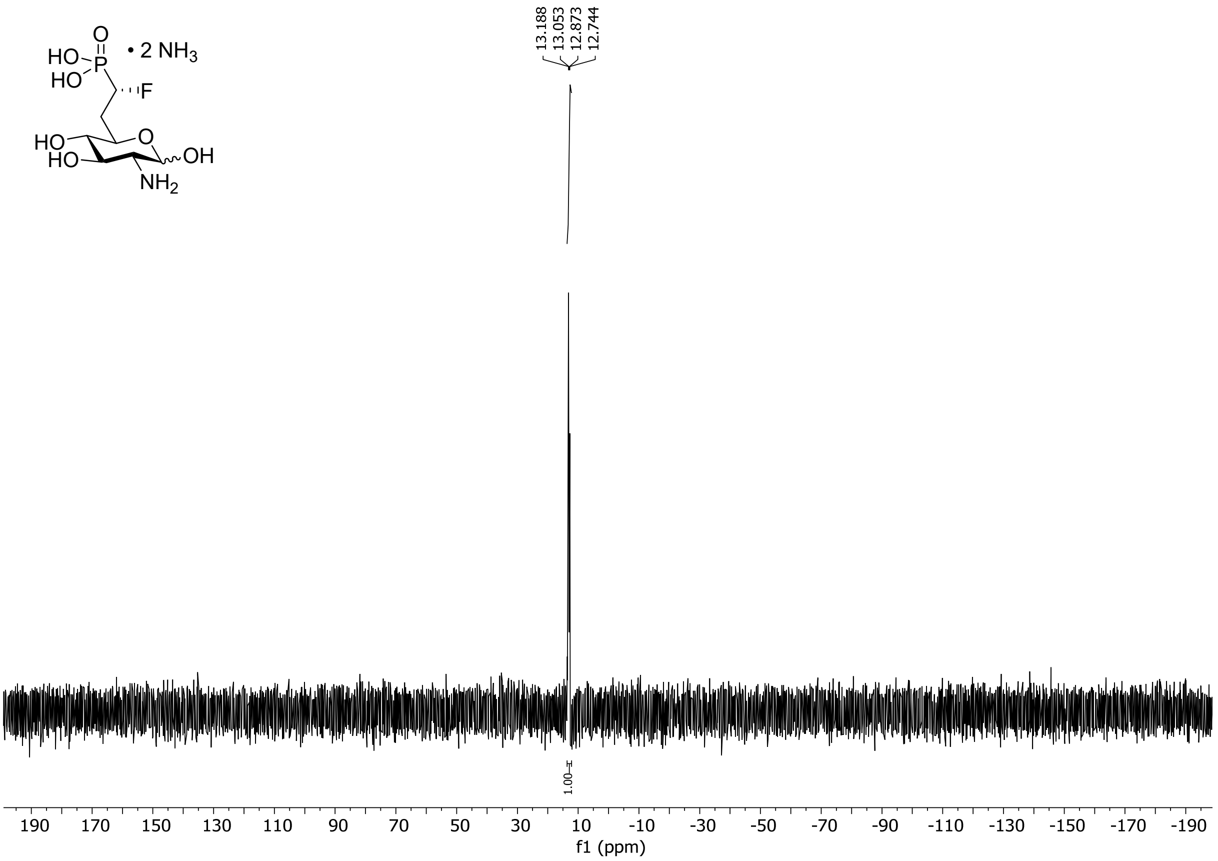

^{31}P NMR spectrum (202 MHz, D_2O) of (*S*)-**3** • 2 NH_3

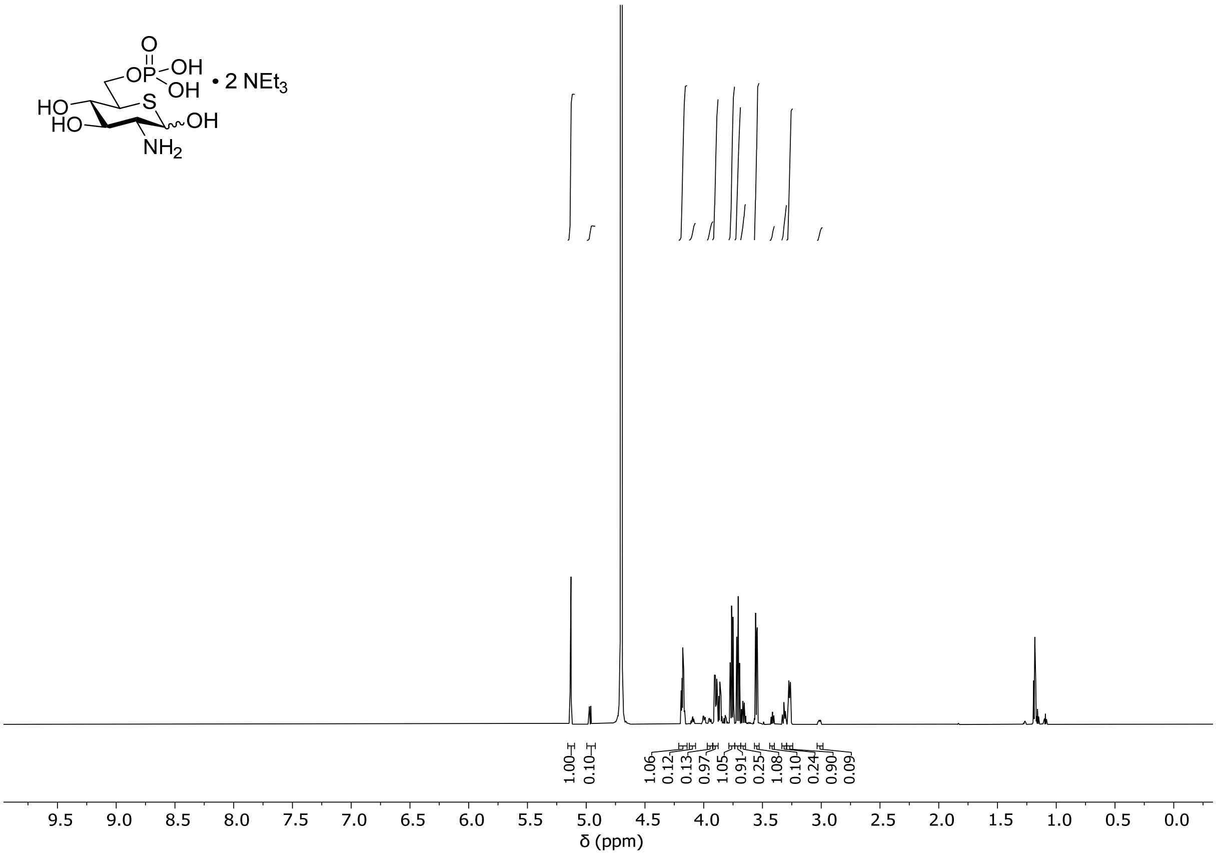

^{1}H NMR spectrum (800 MHz, D_2O) of **4** • 2 NEt_3 with NEt_3 suppression

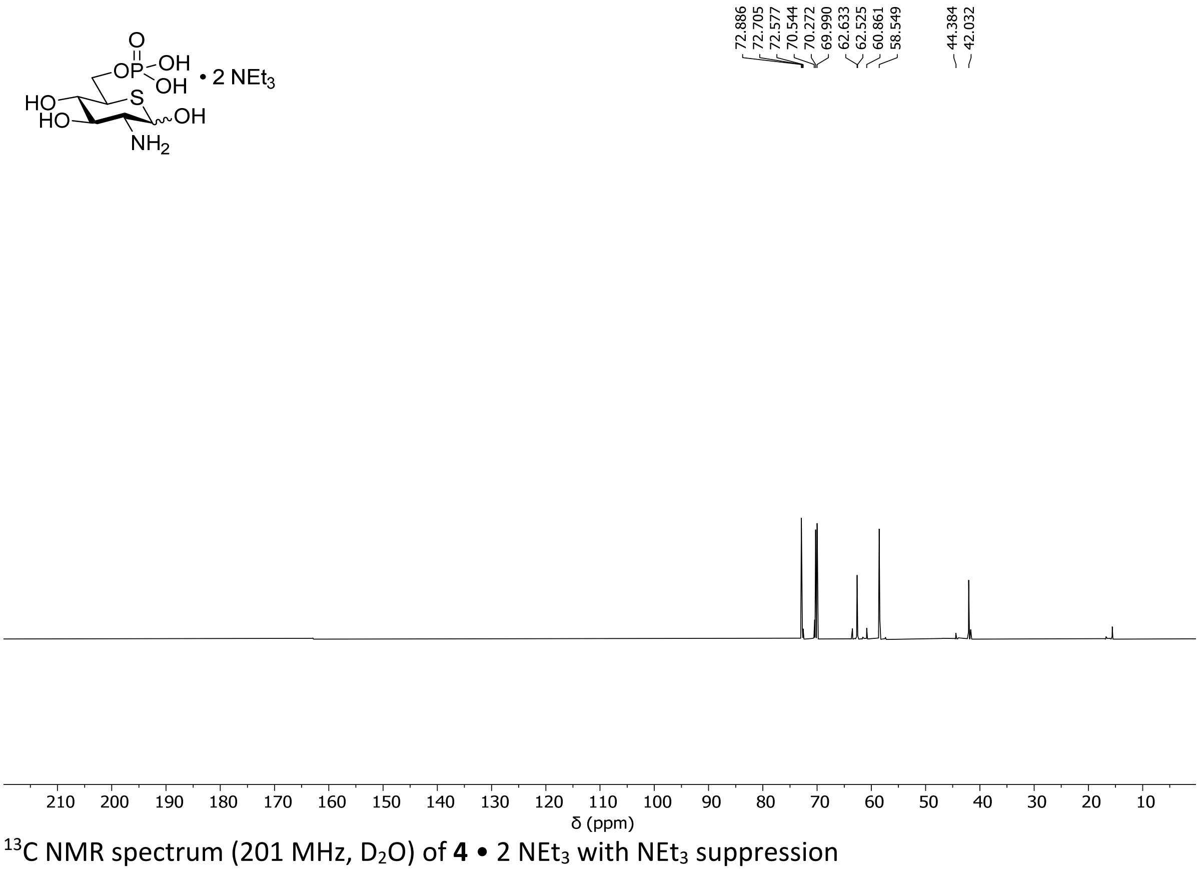

^{13}C NMR spectrum (201 MHz, D_2O) of **4** • 2 NEt_3 with NEt_3 suppression

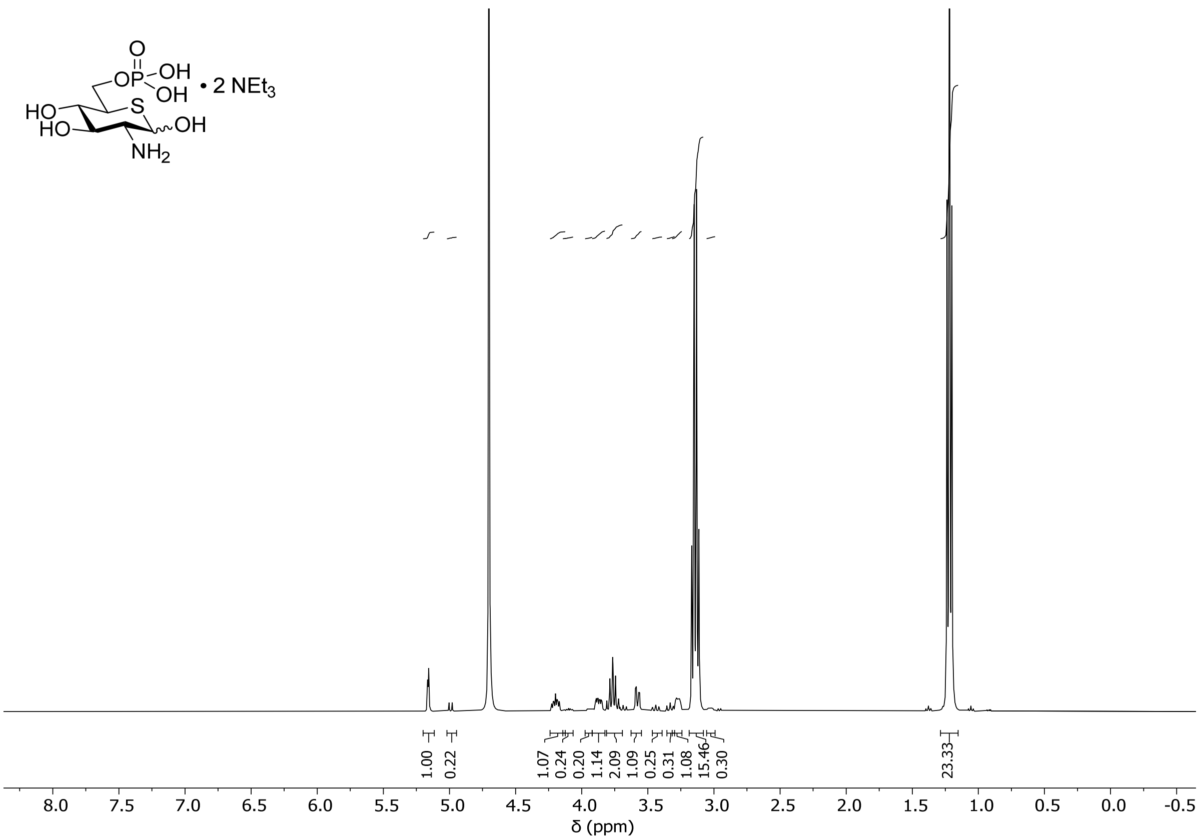

^{1}H NMR spectrum (400 MHz, D_2O) of **4** • 2 NEt_3 without NEt_3 suppression

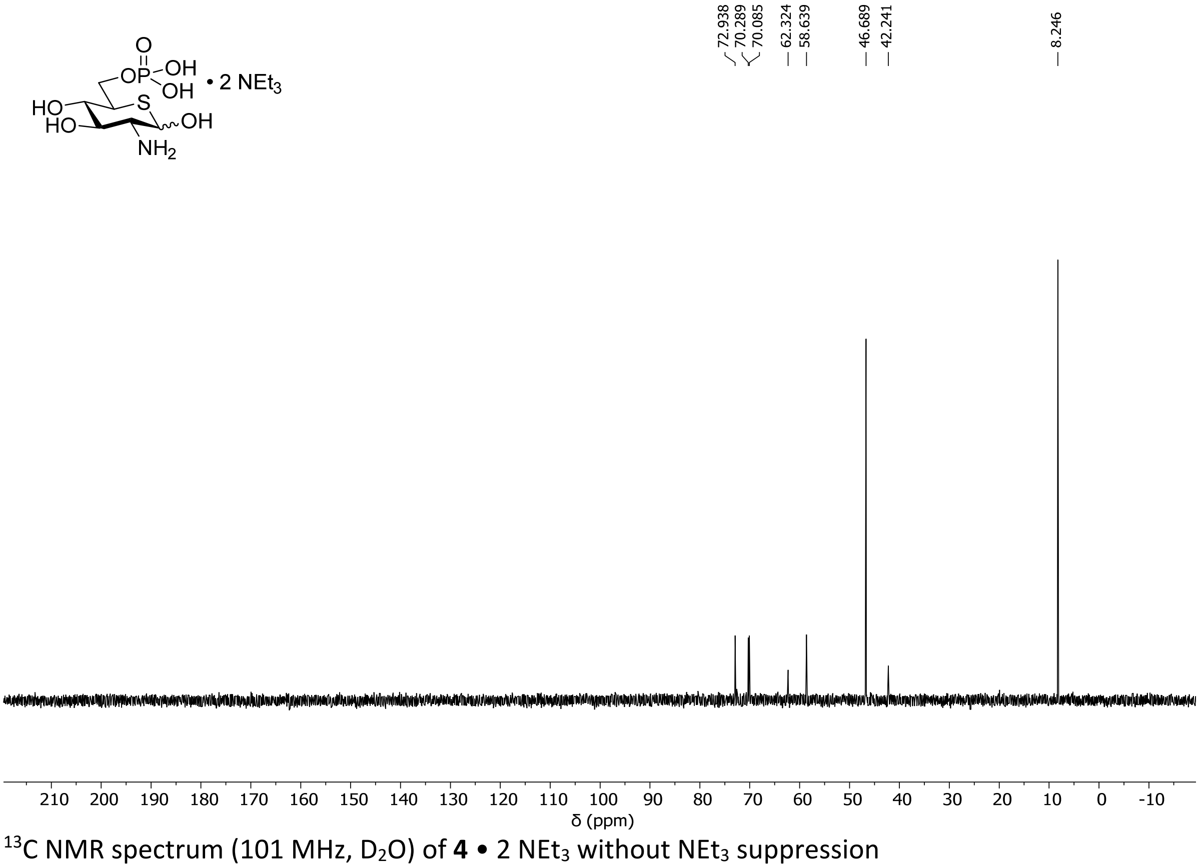

^{13}C NMR spectrum (101 MHz, D_2O) of **4** • 2 NEt_3 without NEt_3 suppression

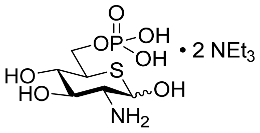

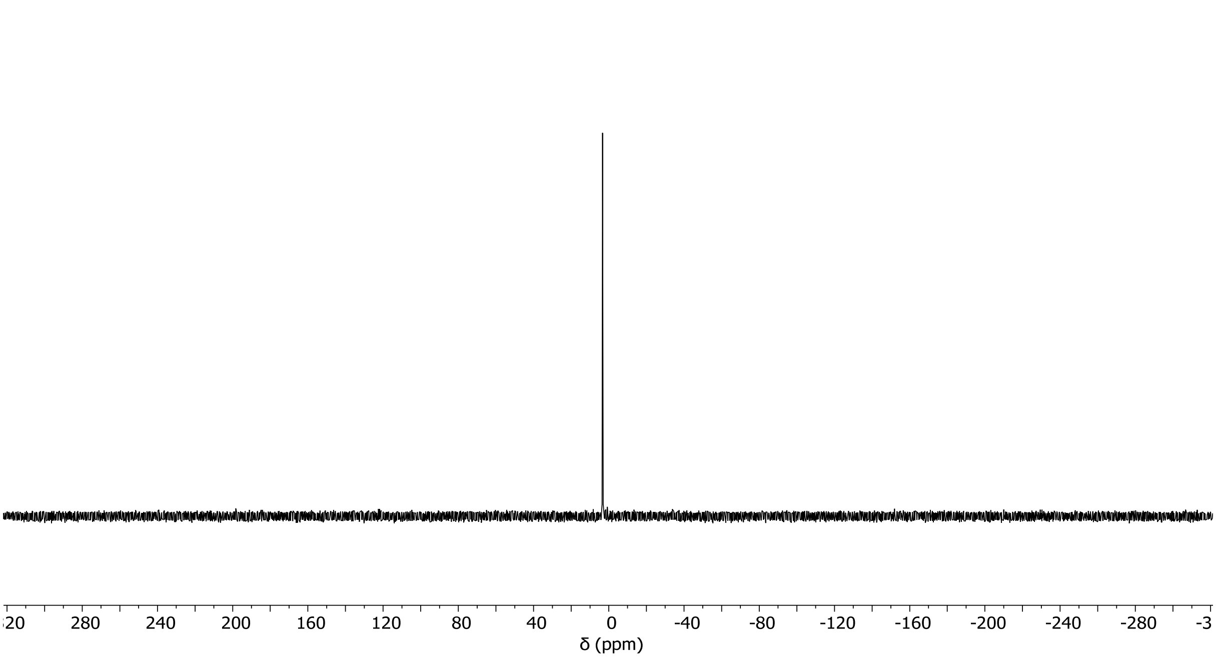

^{31}P NMR spectrum (162MHz, D_2O) of **4** • 2 NEt_3

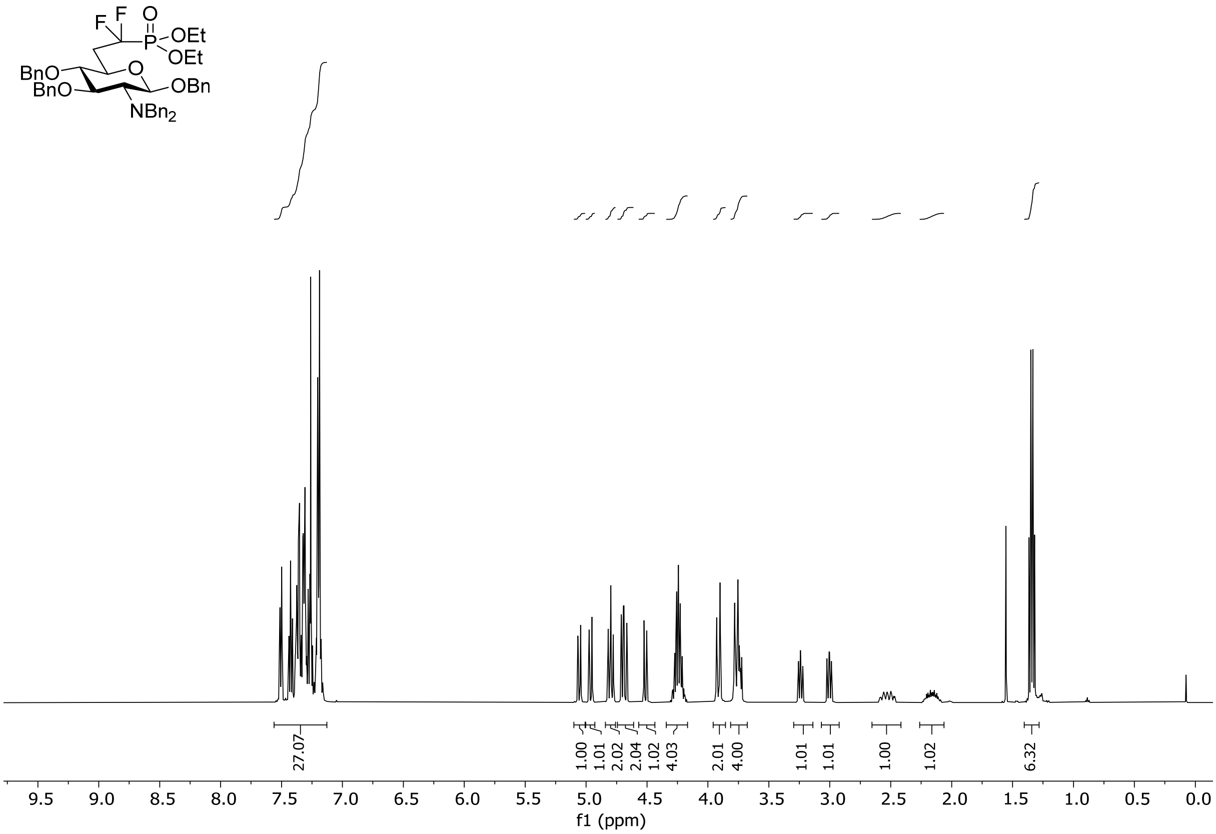

^{1}H NMR spectrum (500 MHz, $CDCl_3$) of **6**

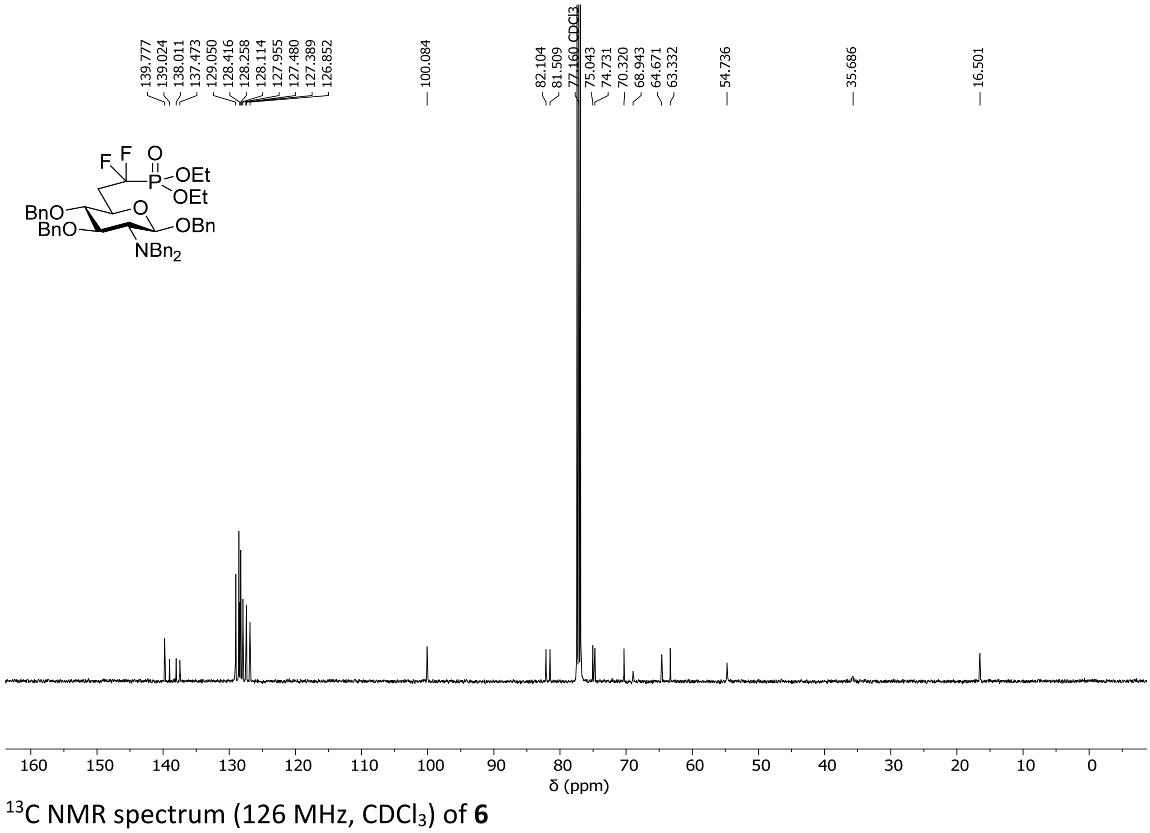

^{13}C NMR spectrum (126 MHz, $CDCl_3$) of **6**

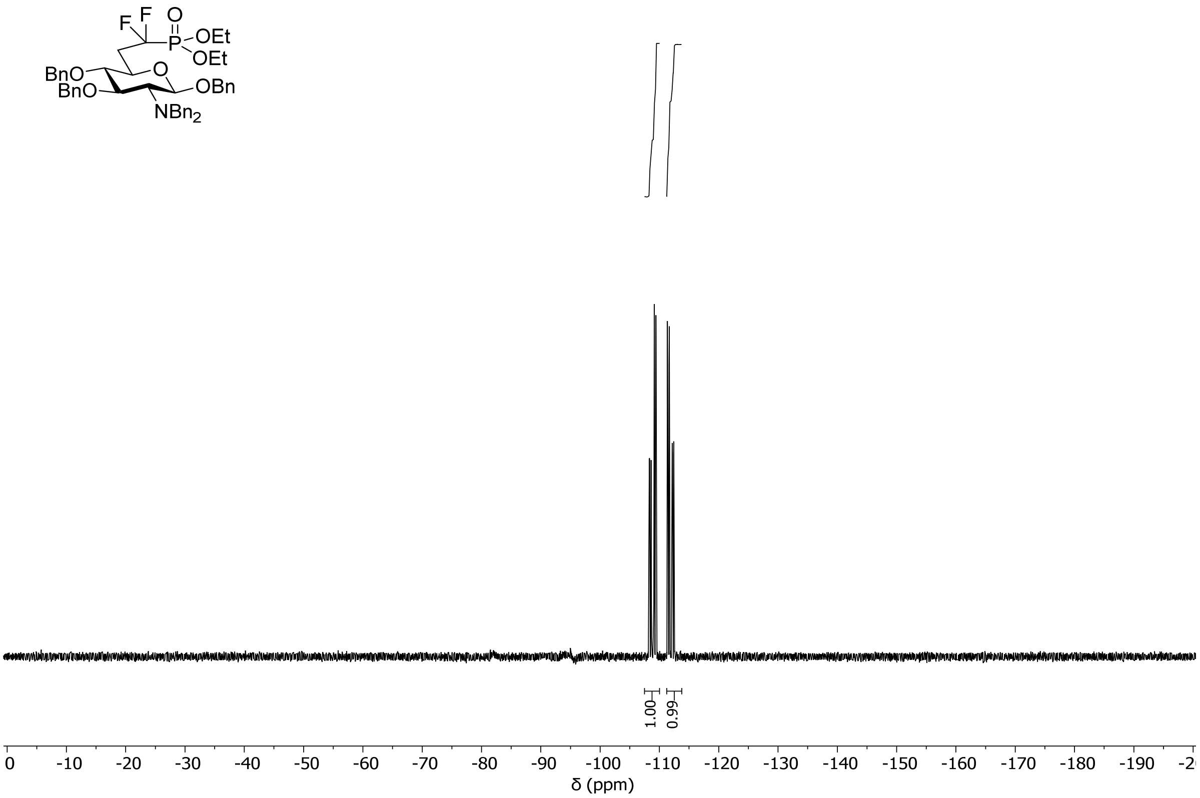

^{19}F NMR spectrum (377MHz, $CDCl_3$) of **6**

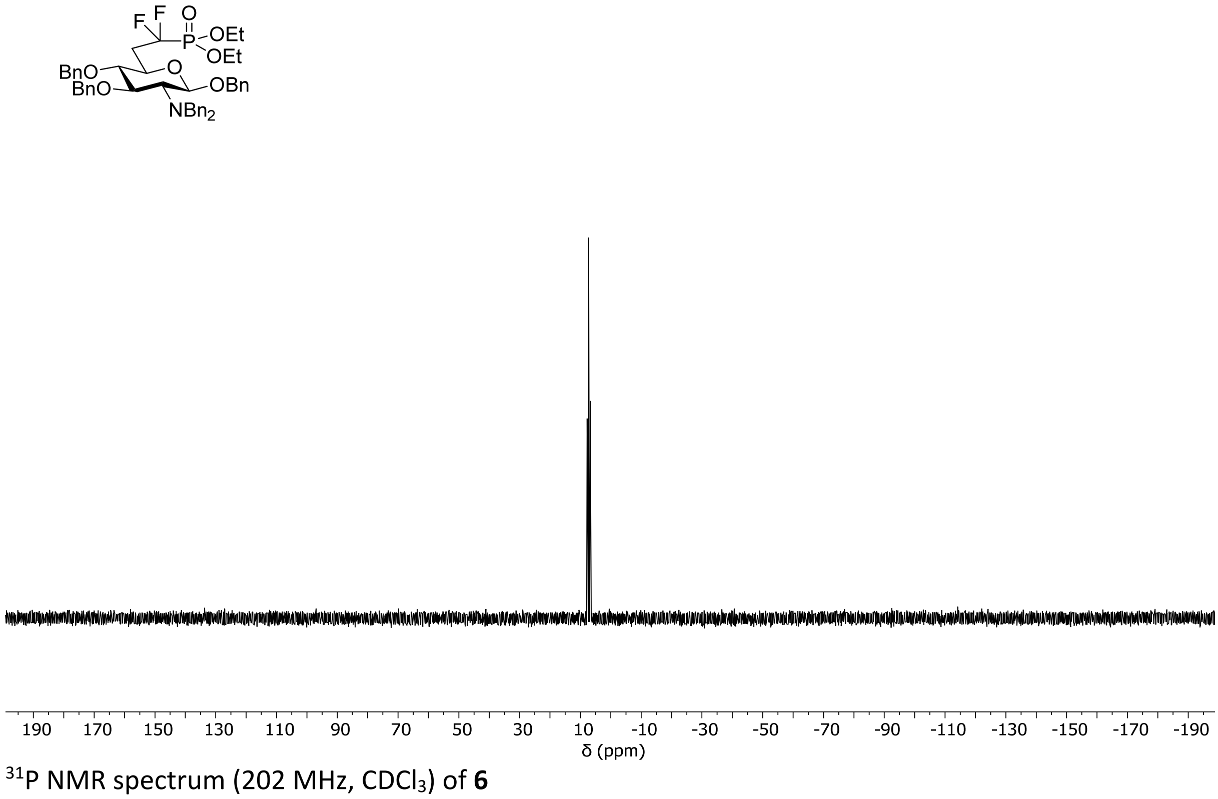

^{31}P NMR spectrum (202 MHz, $CDCl_3$) of **6**

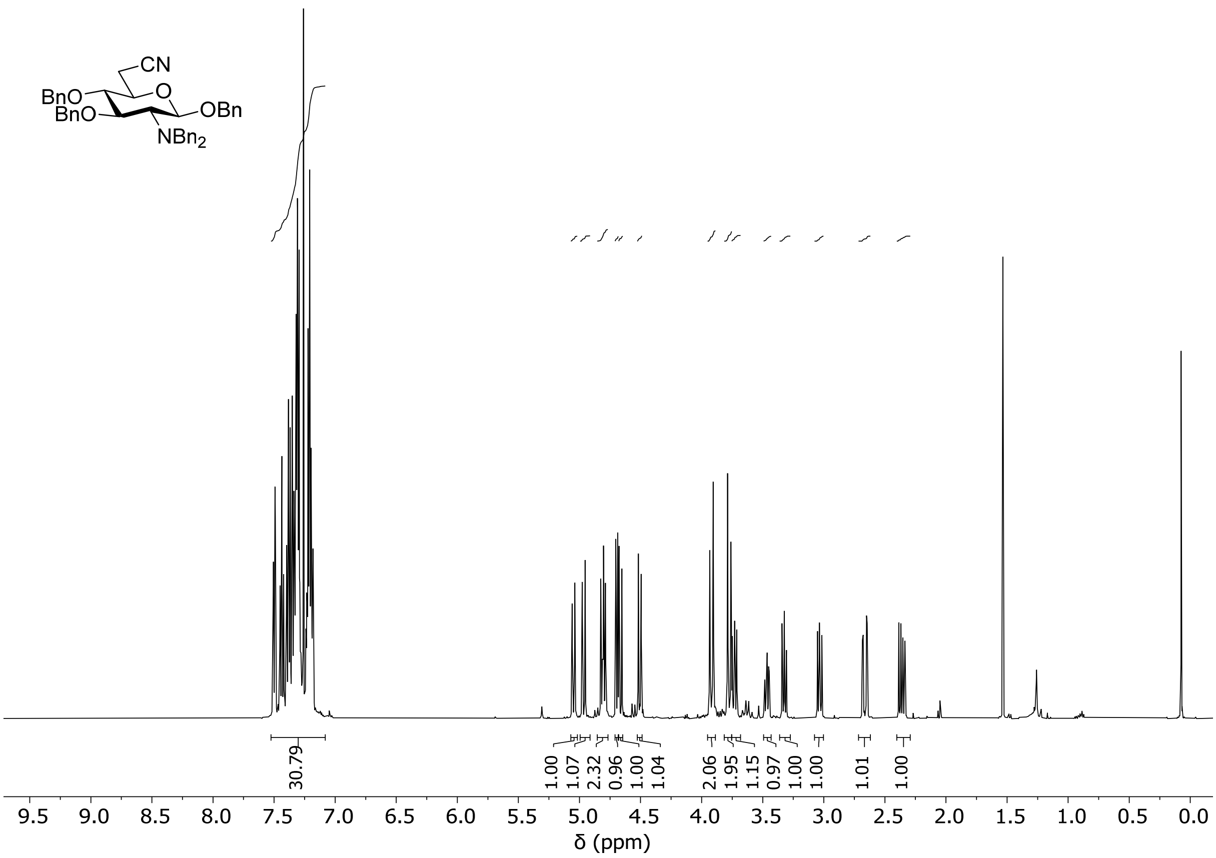

^{1}H NMR spectrum (500 MHz, $CDCl_3$) of **7**

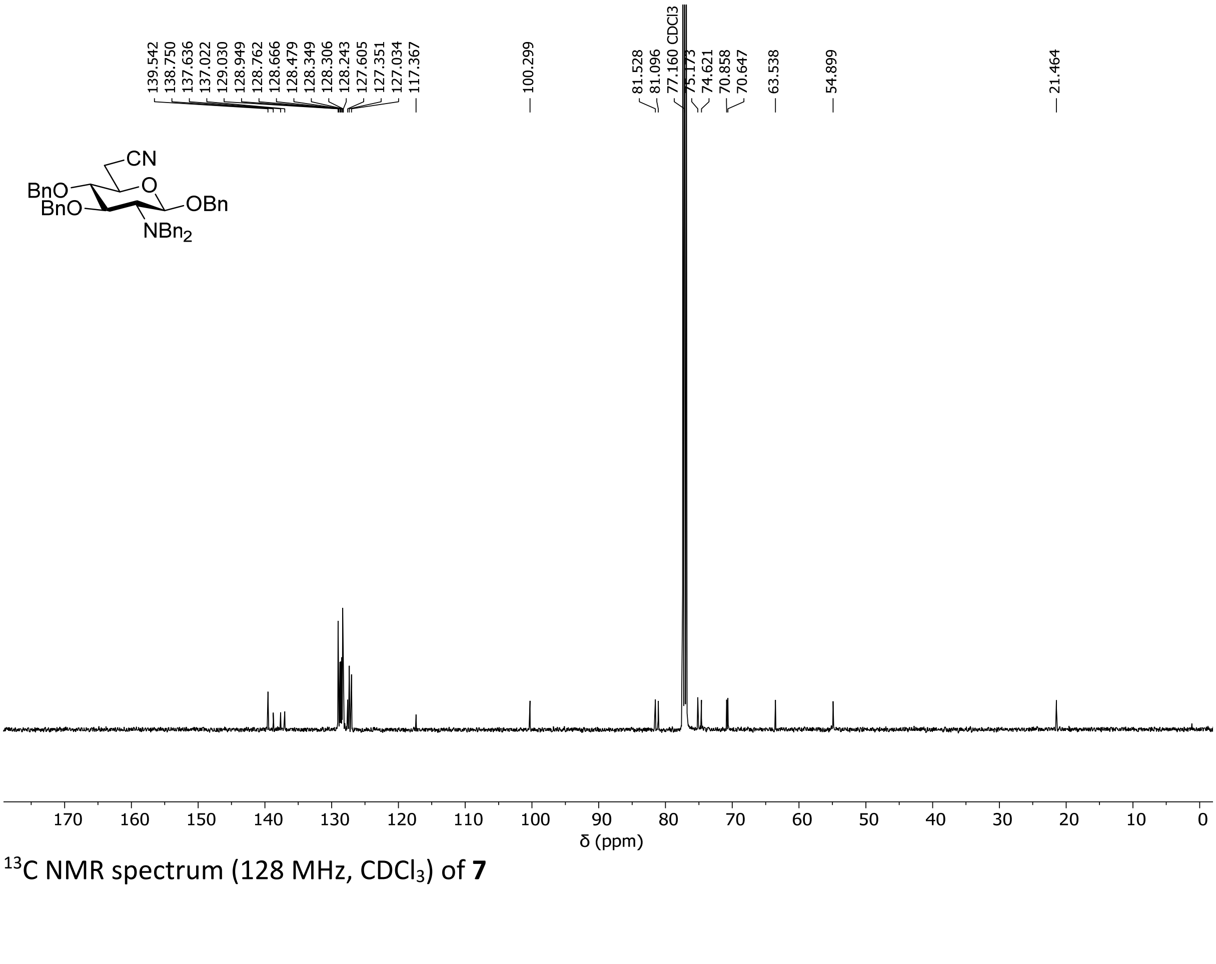

^{13}C NMR spectrum (128 MHz, $CDCl_3$) of **7**

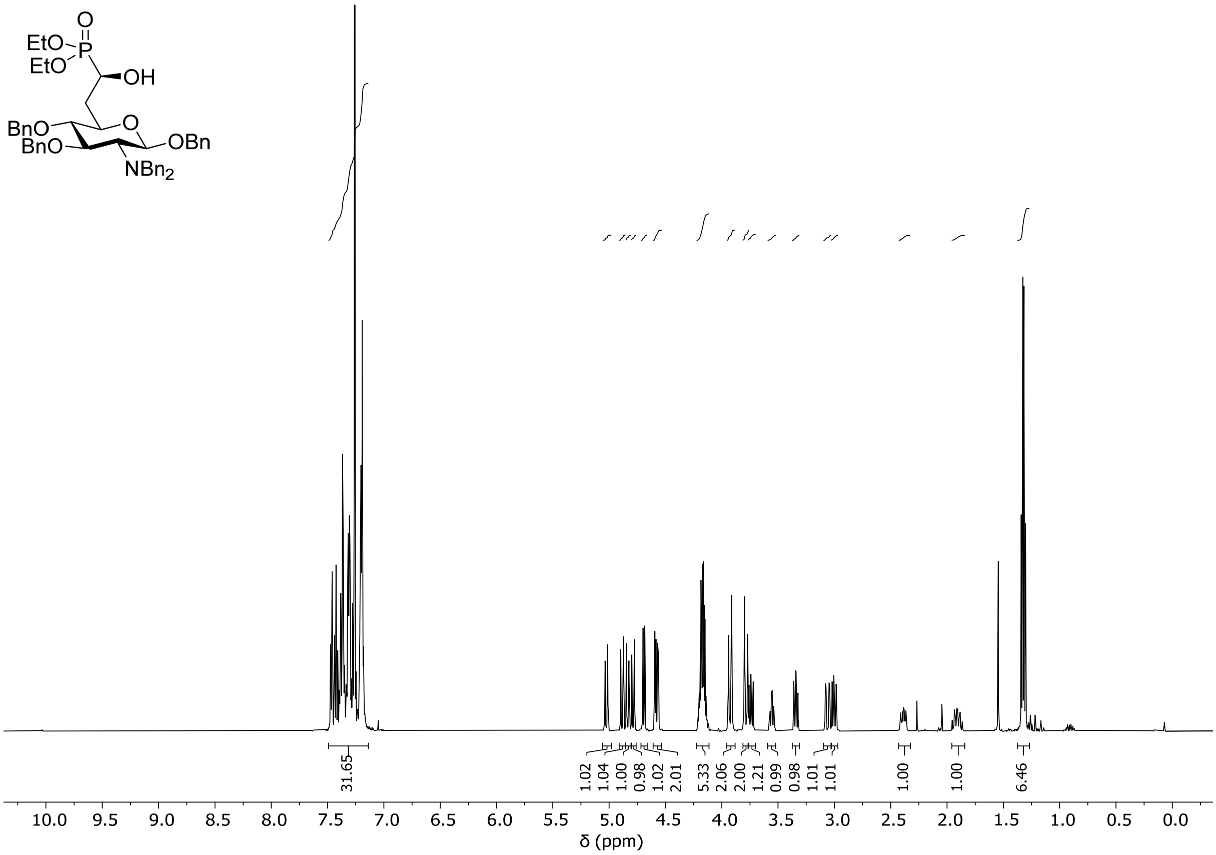

^{1}H NMR spectrum (500 MHz, CDCl$_3$) of (*R*)-**8**

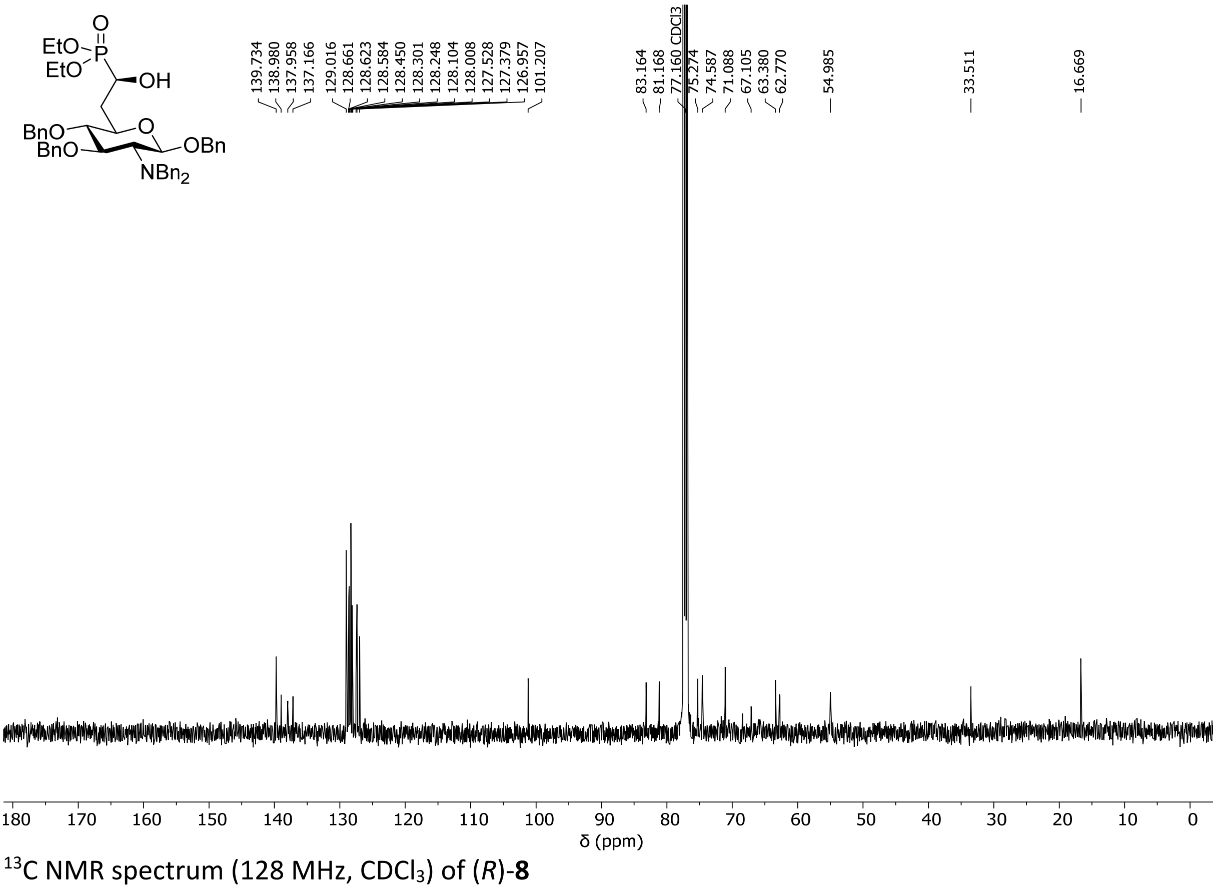

^{13}C NMR spectrum (128 MHz, CDCl$_3$) of (*R*)-**8**

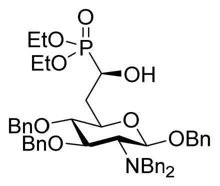

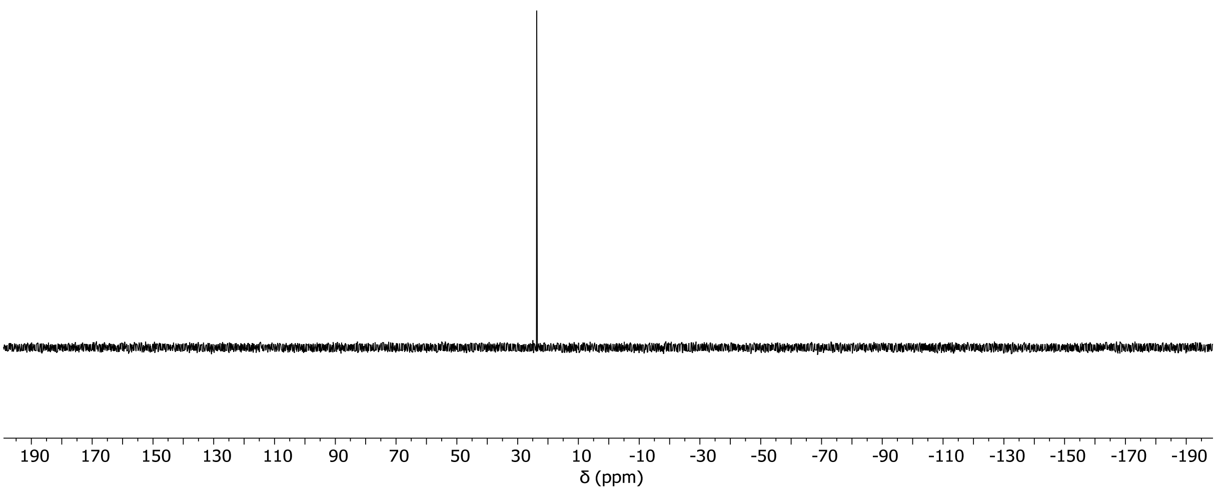

^{31}P NMR spectrum (202 MHz, $CDCl_3$) of (*R*)-**8**

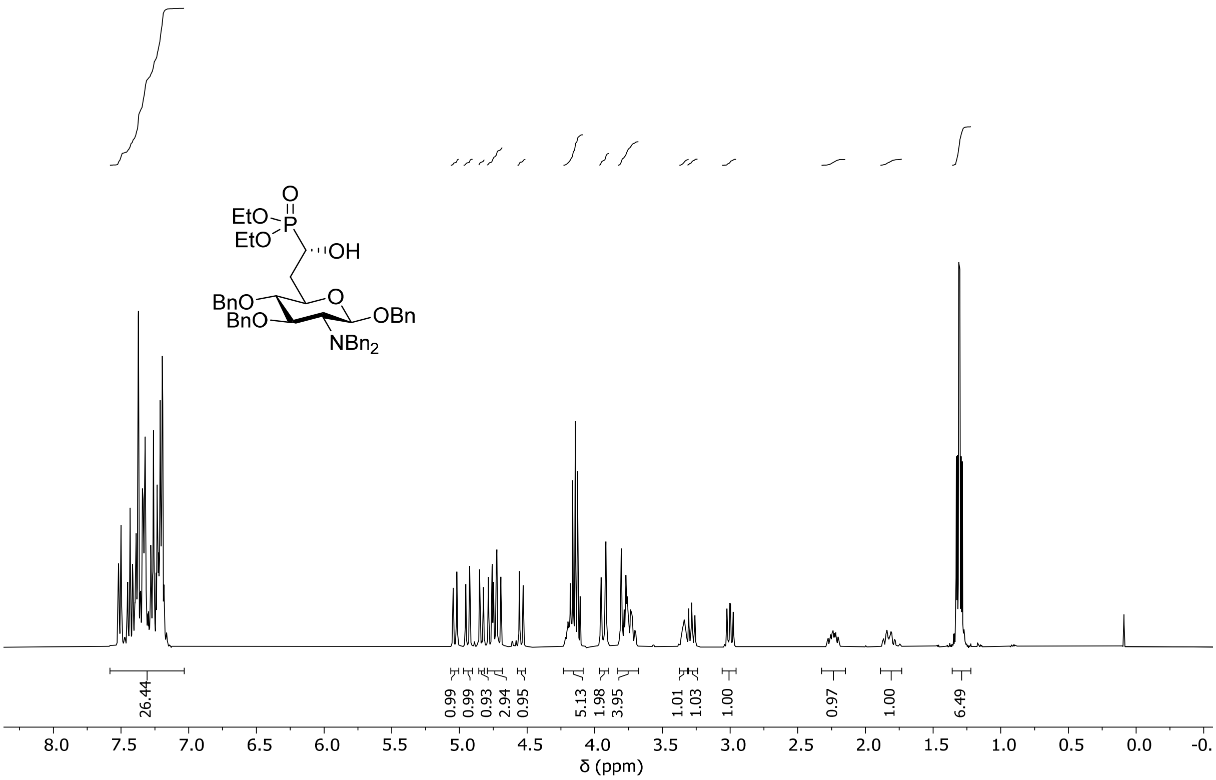

^{1}H NMR spectrum (500 MHz, $CDCl_3$) of (*S*)-**8**

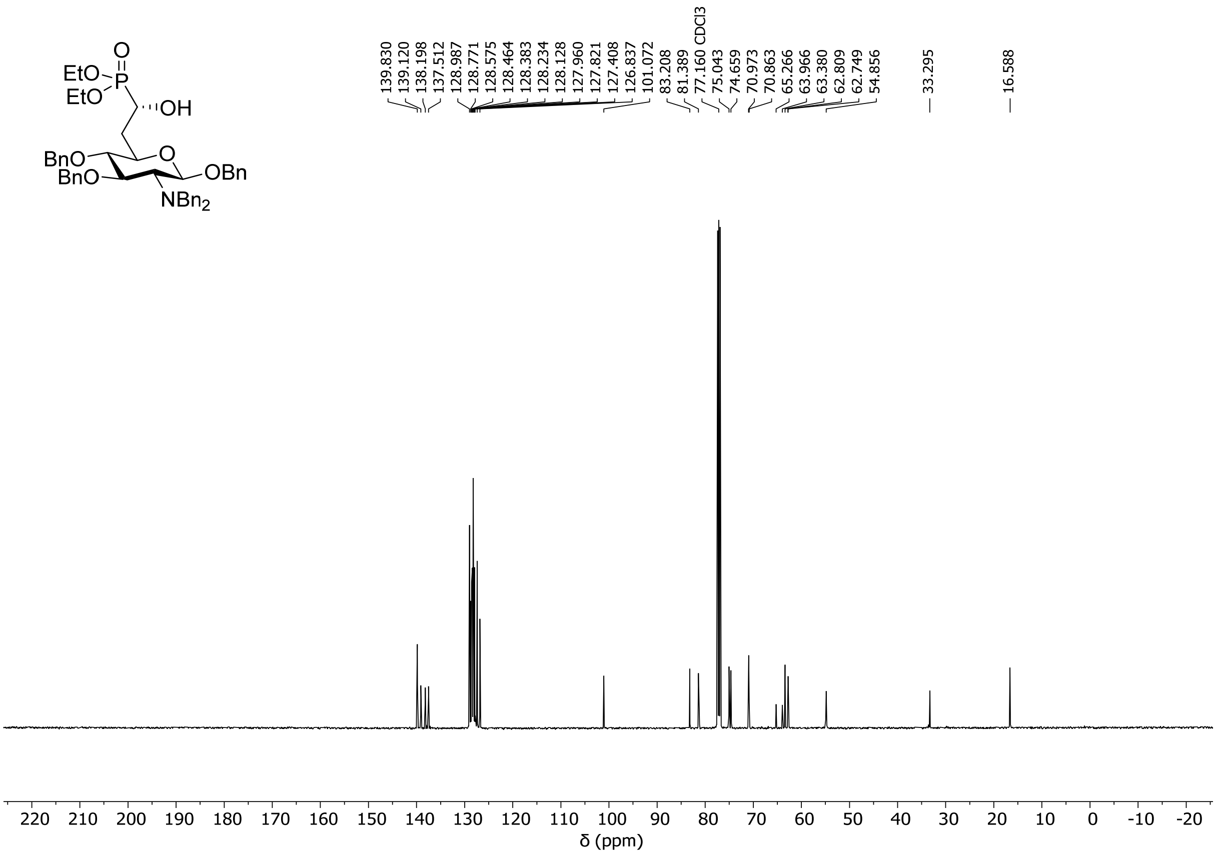

^{13}C NMR spectrum (128 MHz, $CDCl_3$) of (*S*)-**8**

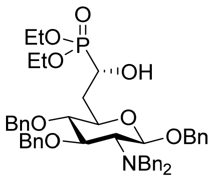

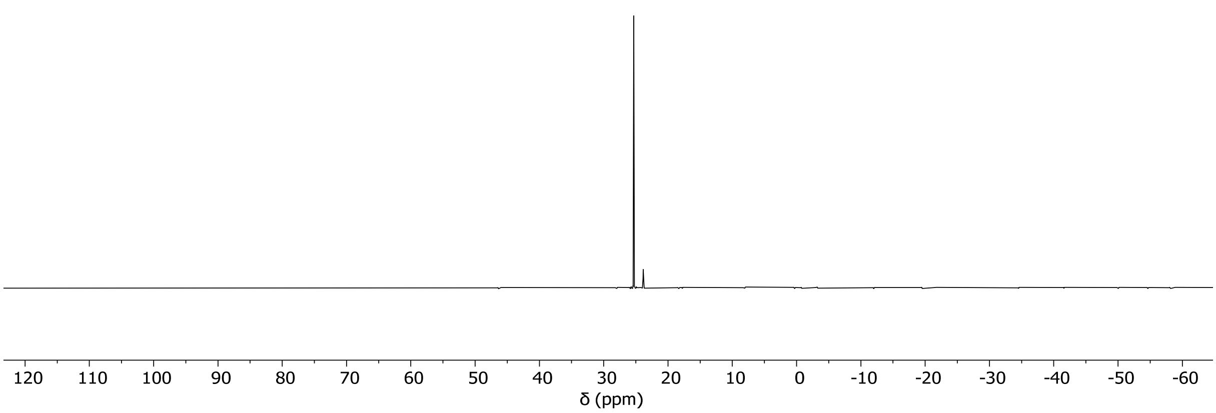

^{31}P NMR spectrum (202MHz, $CDCl_3$) of (*S*)-**8**

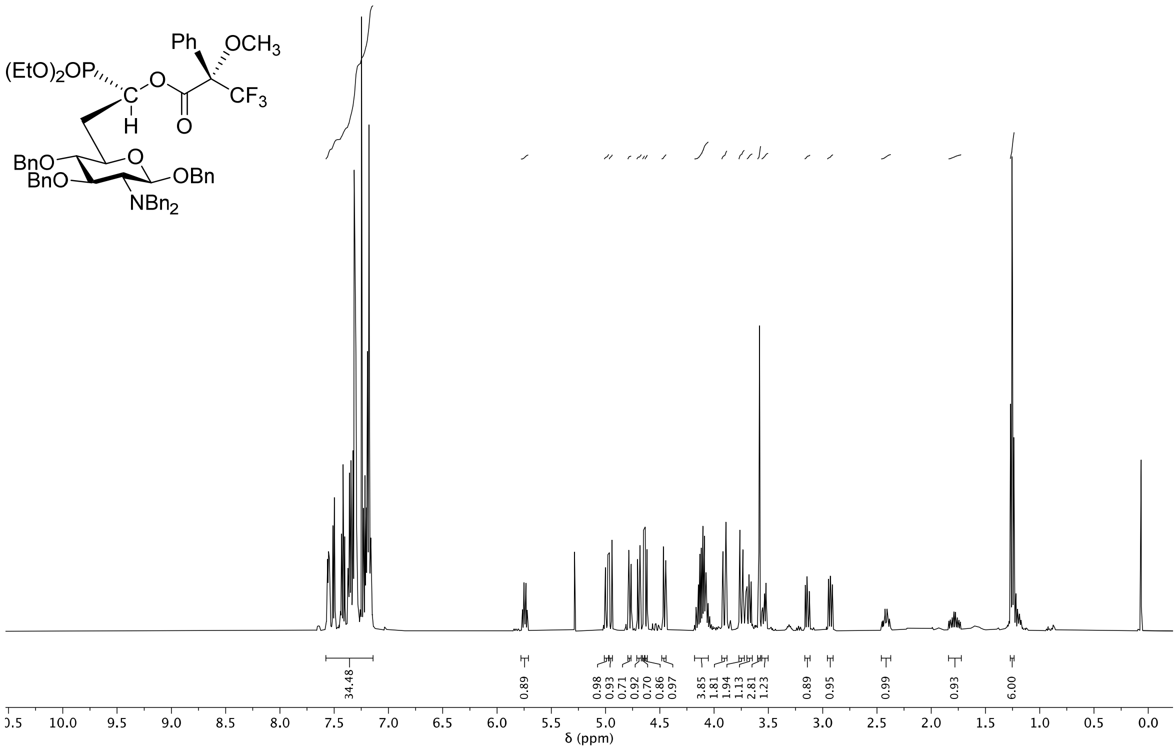

^{1}H-NMR (400 MHz, $CDCl_3$) spectrum of (*R*)-**8**-(*S*)-MTPA ester

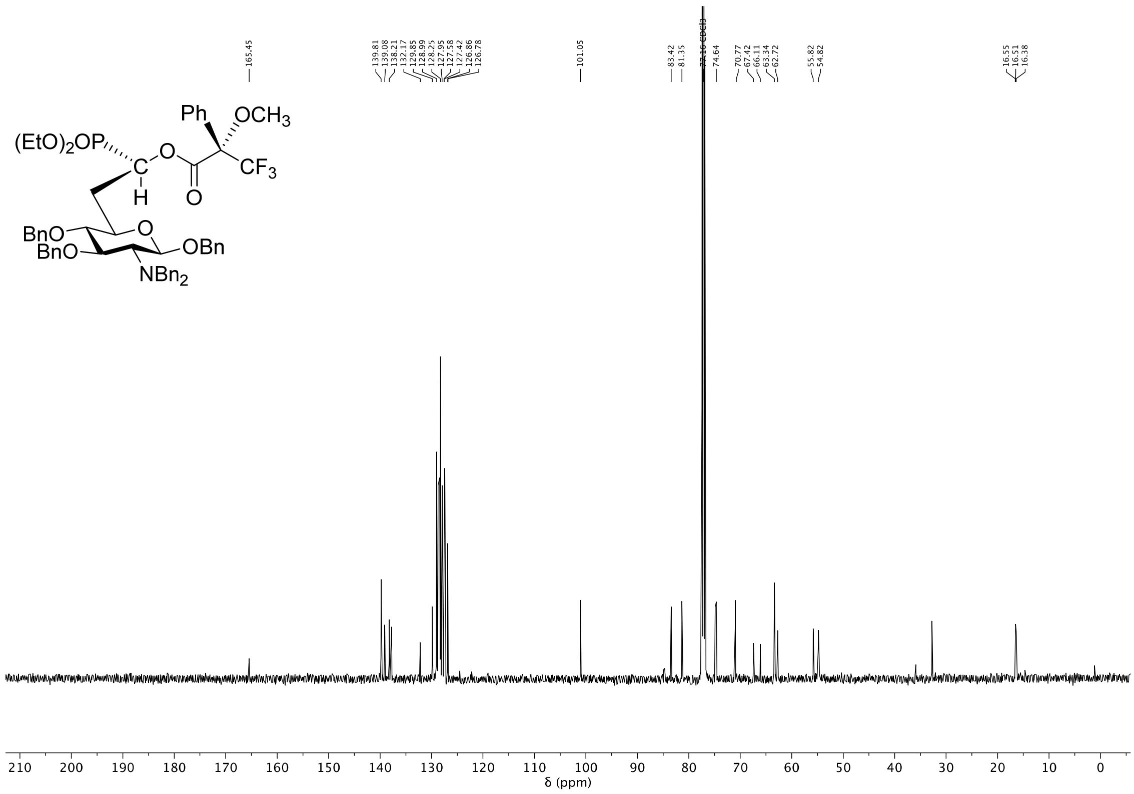

^{13}C-NMR (377 MHz, $CDCl_3$) spectrum of (*R*)-**8**-(*S*)-MTPA ester

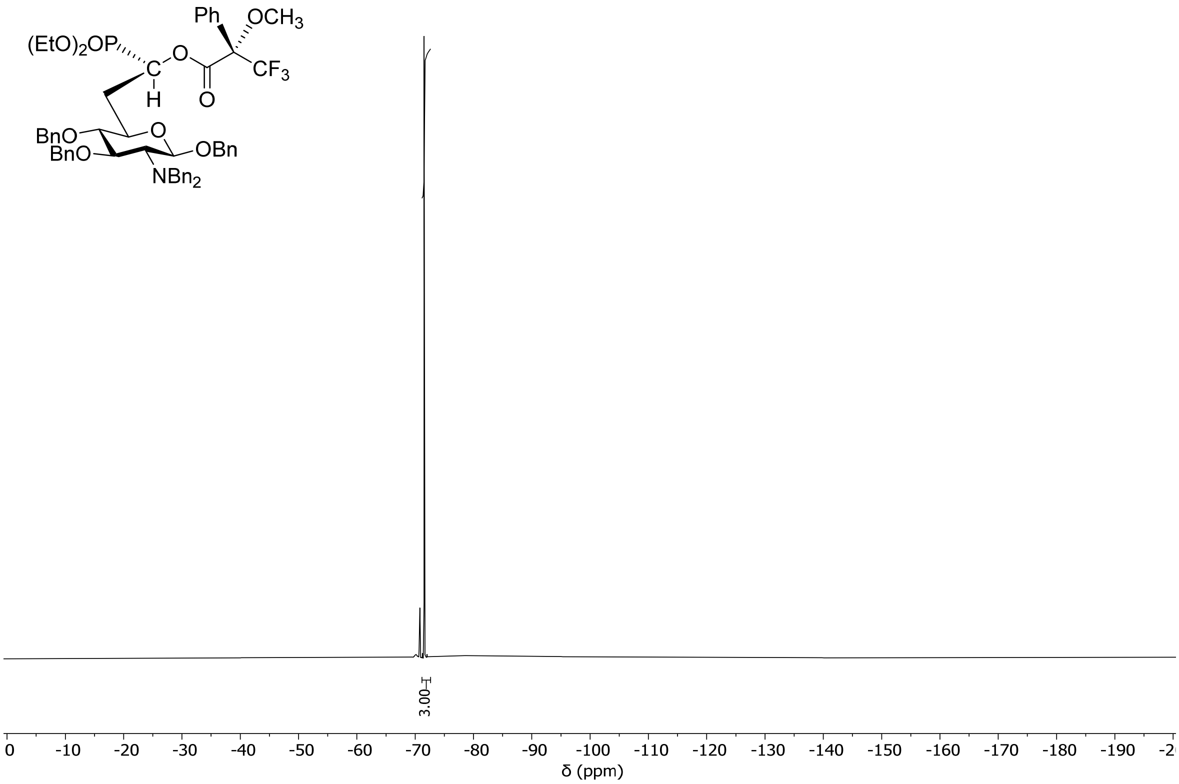

^{19}F NMR spectrum (376 MHz, $CDCl_3$) of (*R*)-**8**-(*S*)-MTPA ester

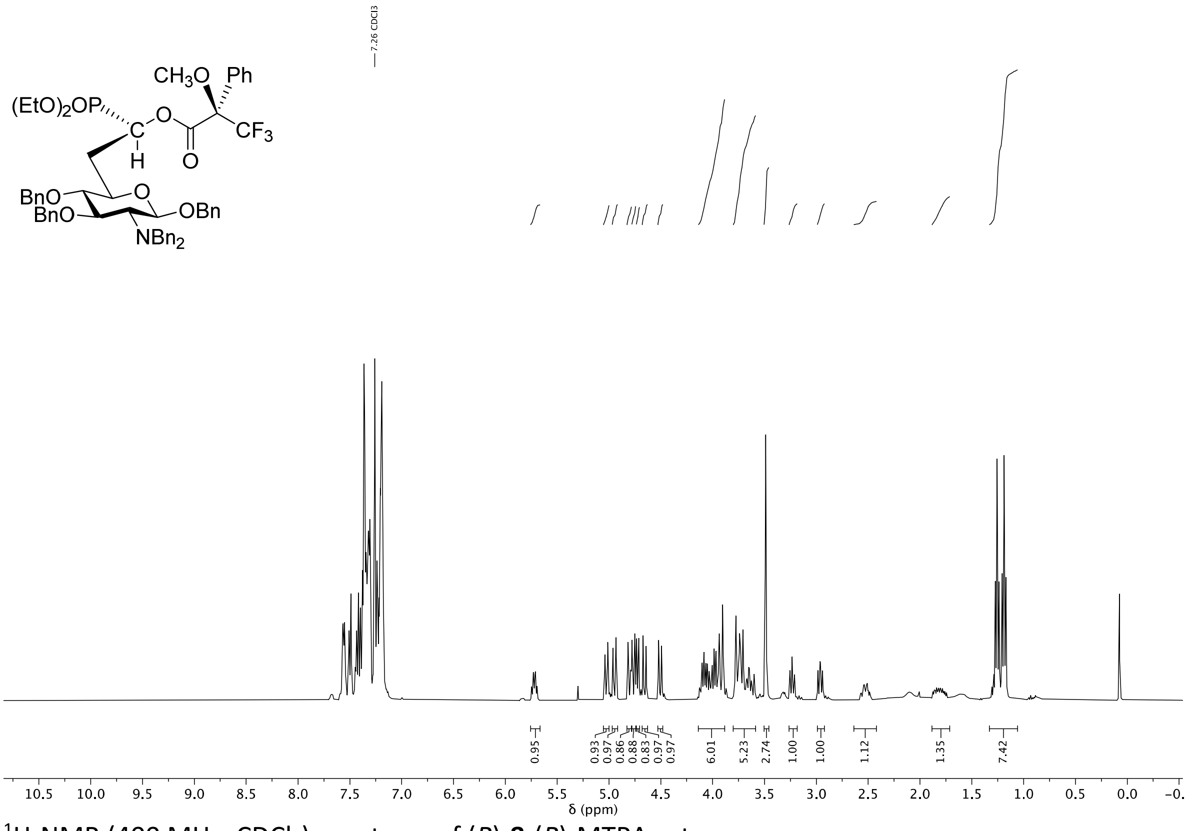

^{1}H-NMR (400 MHz, $CDCl_3$) spectrum of (*R*)-**8**-(*R*)-MTPA ester

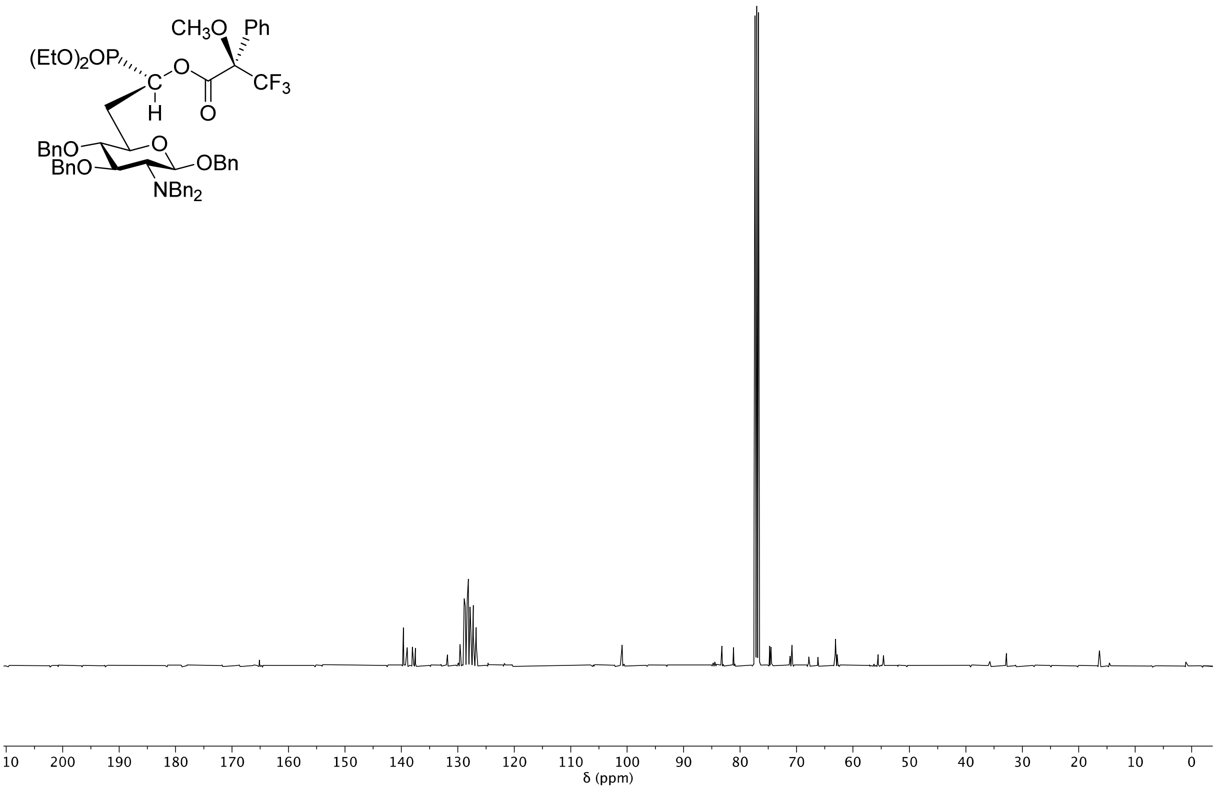

^{13}C-NMR (101 MHz, $CDCl_3$) spectrum of (*R*)-**8**-(*R*)-MTPA ester

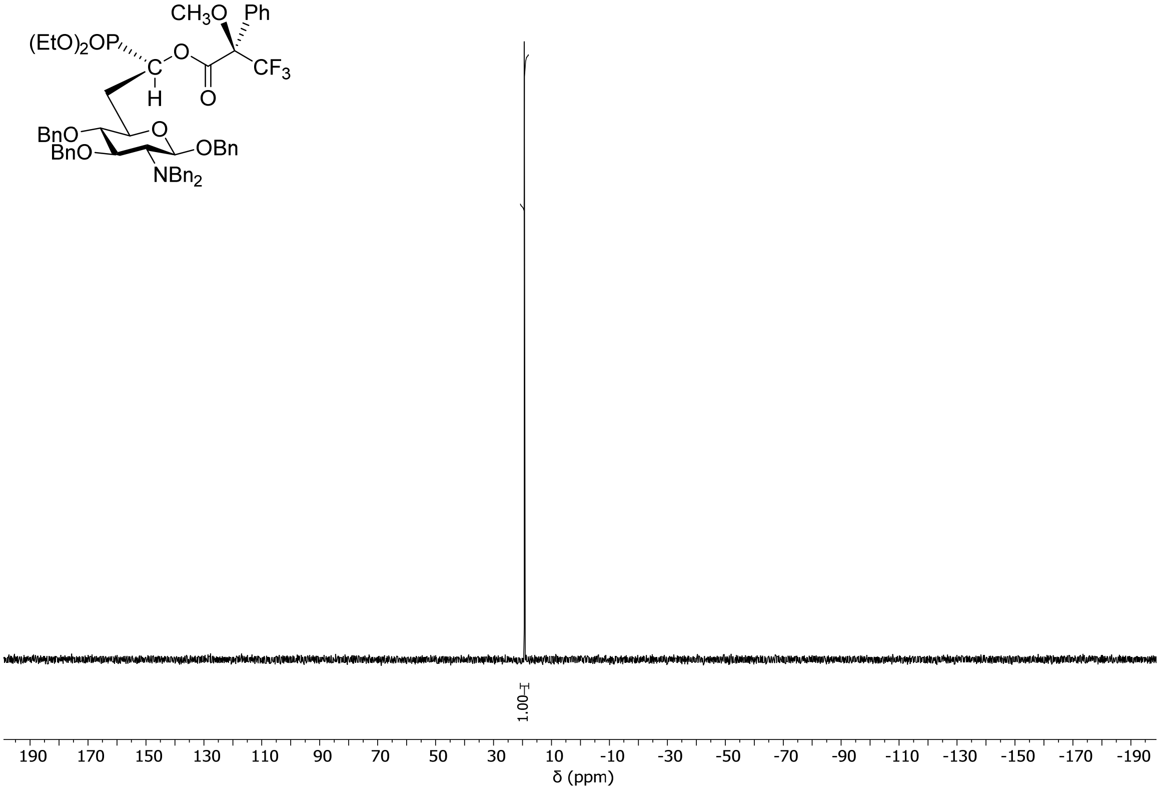

^{19}F NMR spectrum (471 MHz, $CDCl_3$) of (*R*)-**8**-(*R*)-MTPA ester

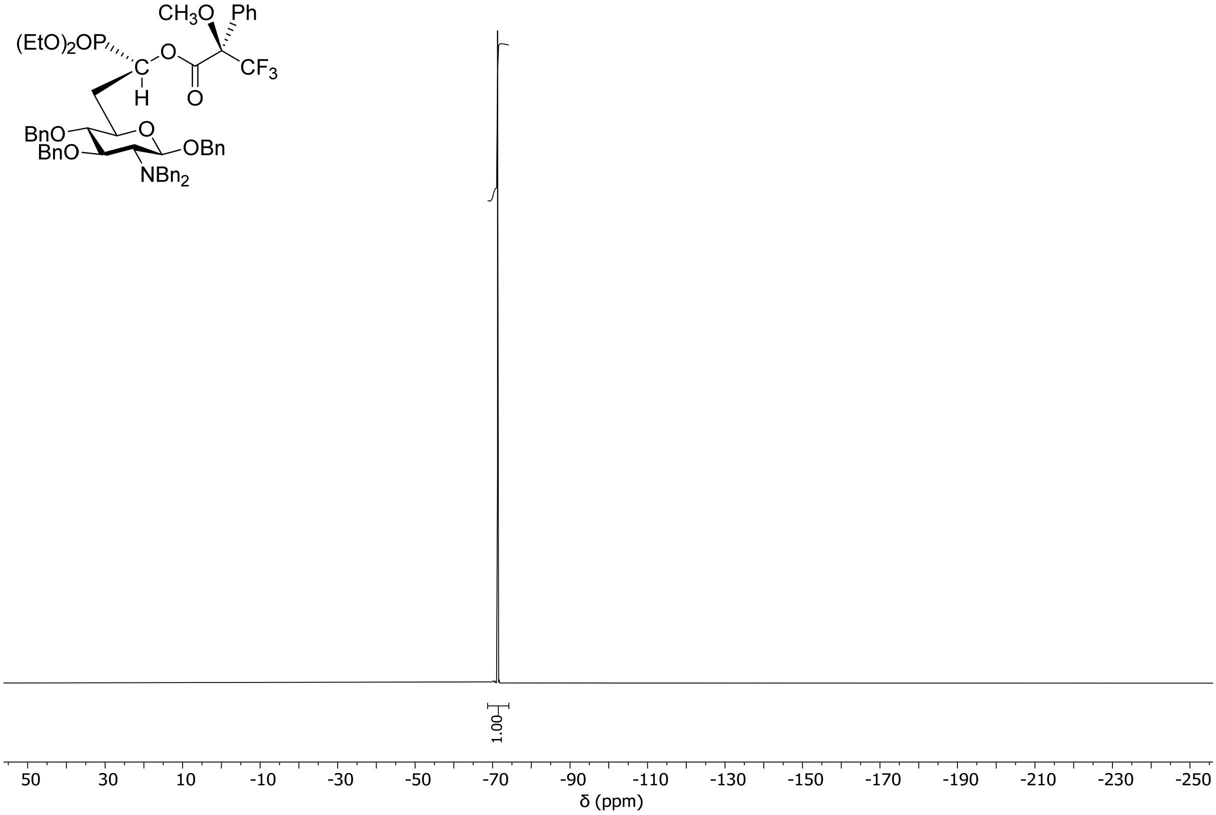

^{31}P NMR spectrum (202MHz, $CDCl_3$) of (*R*)-**8**-(*R*)-MTPA ester

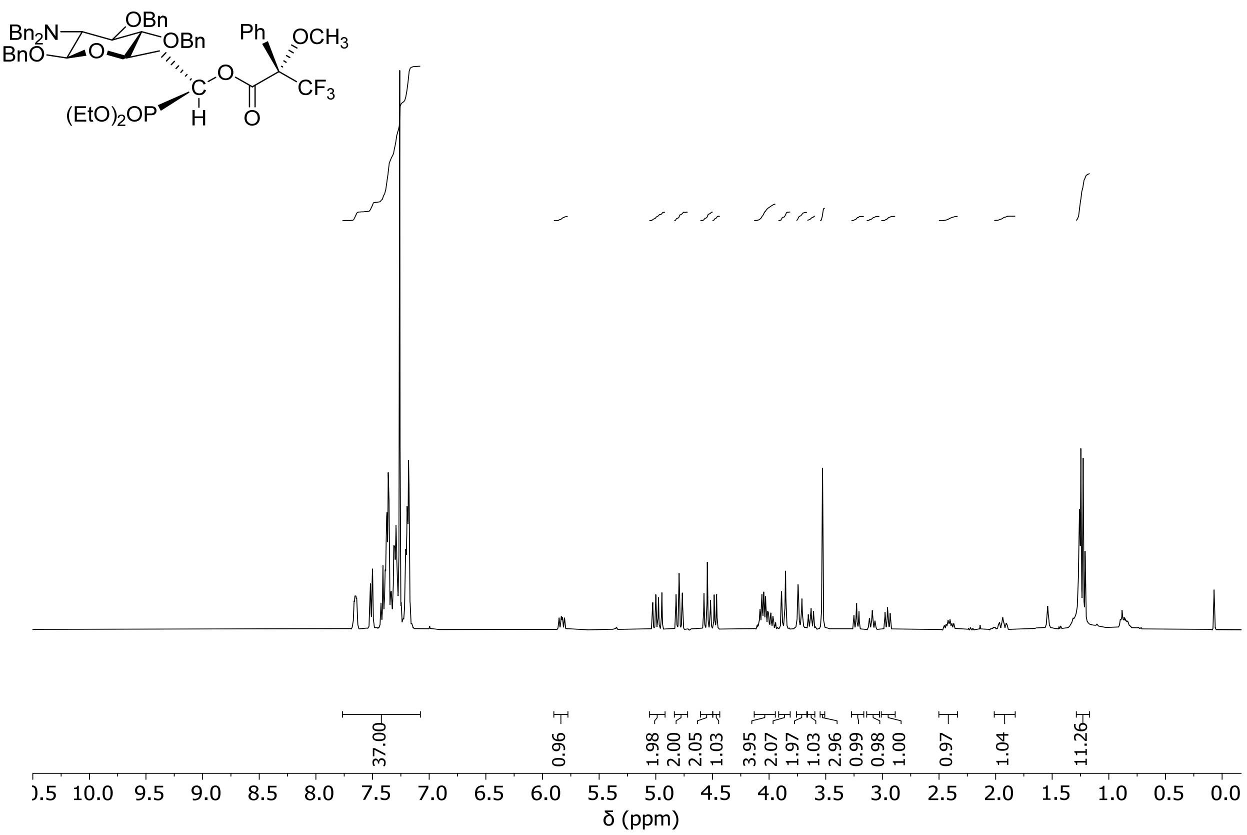

^{1}H-NMR (400 MHz, $CDCl_3$) spectrum of (*S*)-**8**-(*S*)-MTPA ester

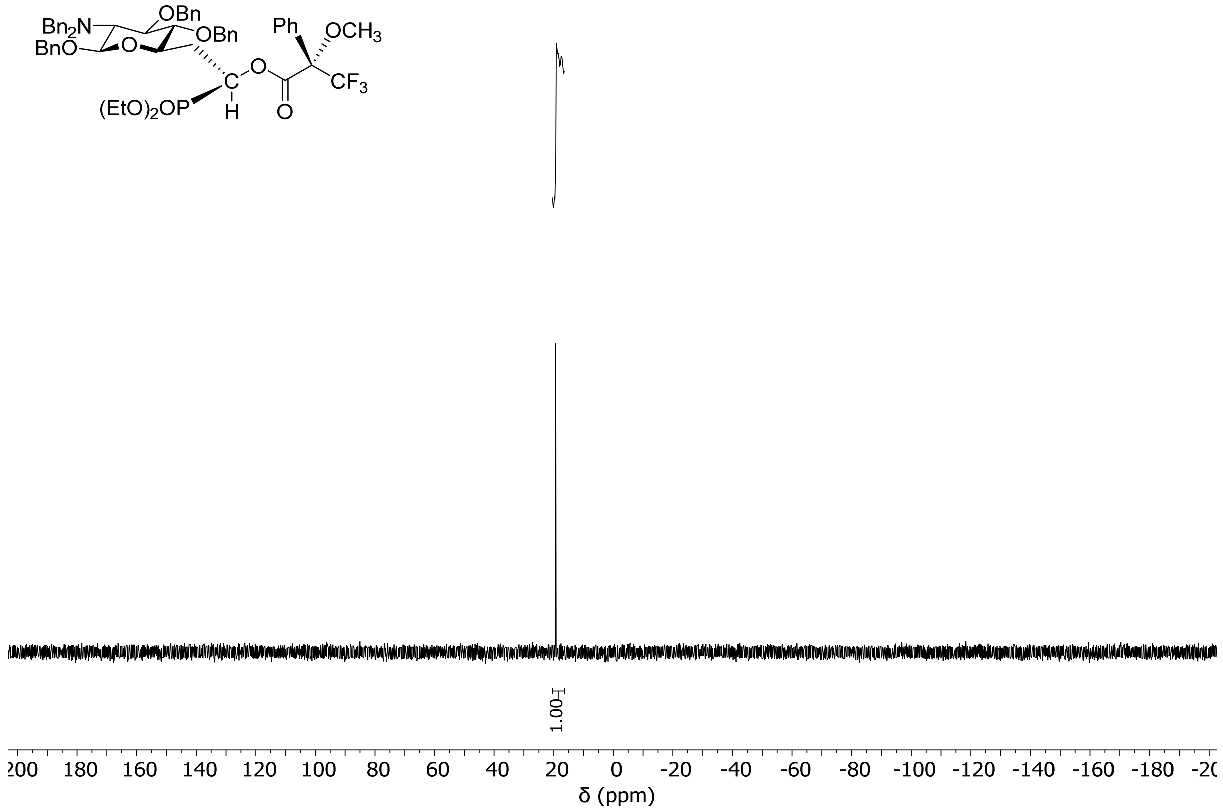

^{31}P NMR spectrum (202MHz, $CDCl_3$) of (*S*)-**8**-(*S*)-MTPA ester

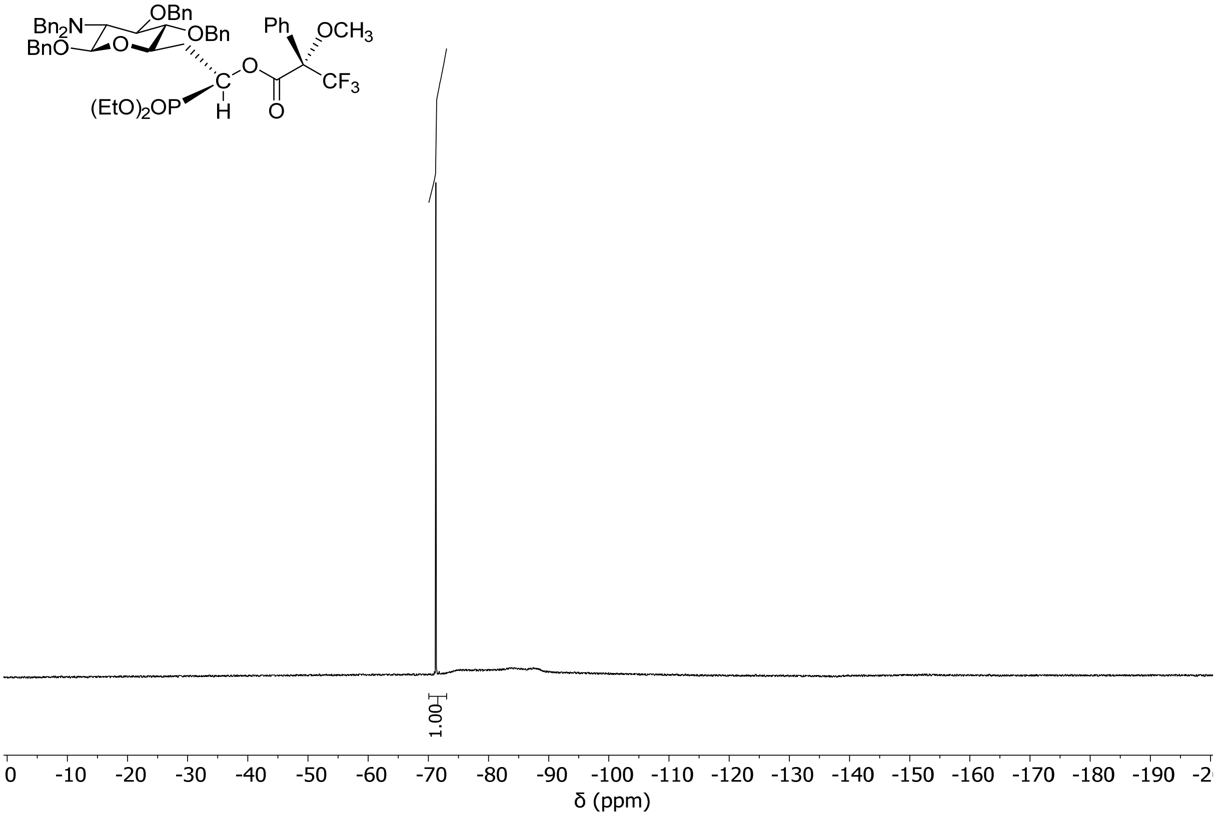

^{19}F NMR spectrum (471 MHz, $CDCl_3$) of (*S*)-**8**-(*S*)-MTPA ester

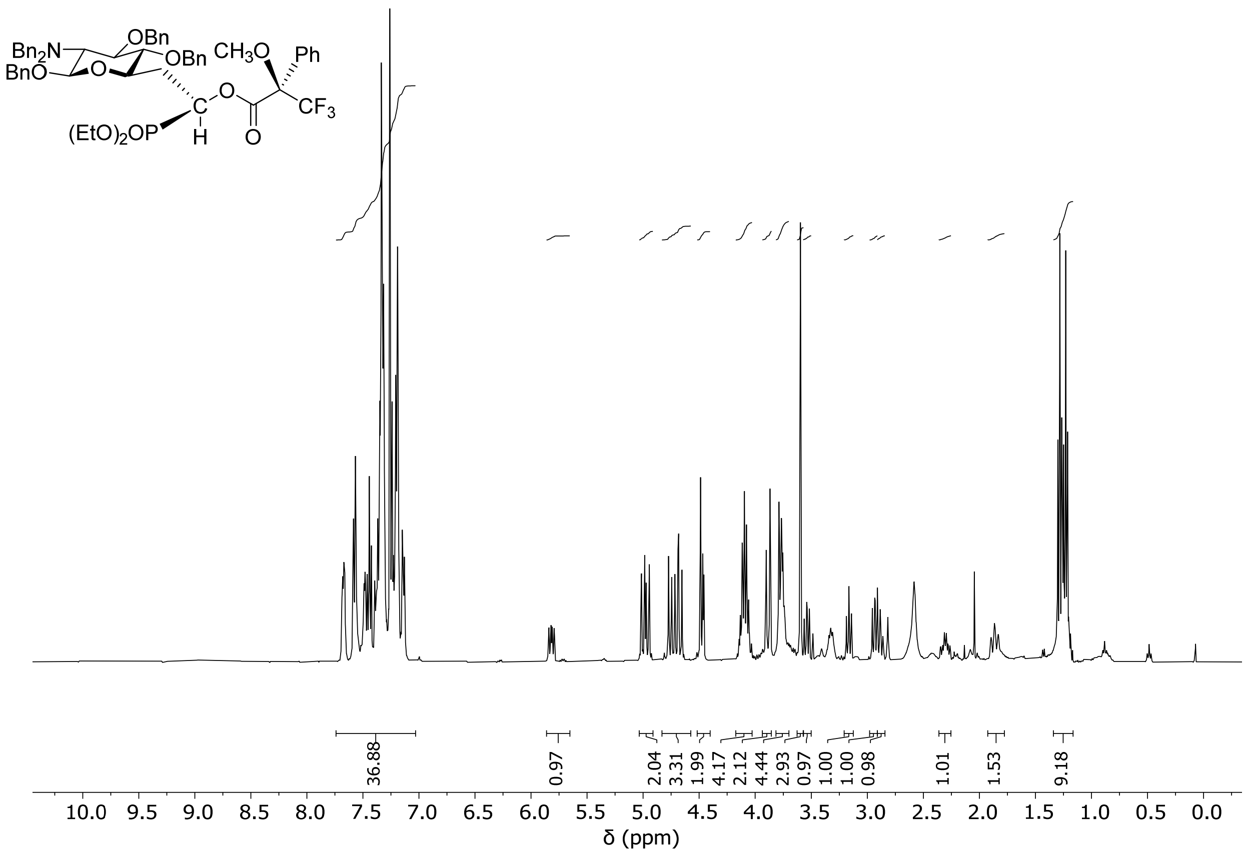

^{1}H-NMR (400 MHz, $CDCl_3$) spectrum of (*S*)-**8**-(*R*)-MTPA ester

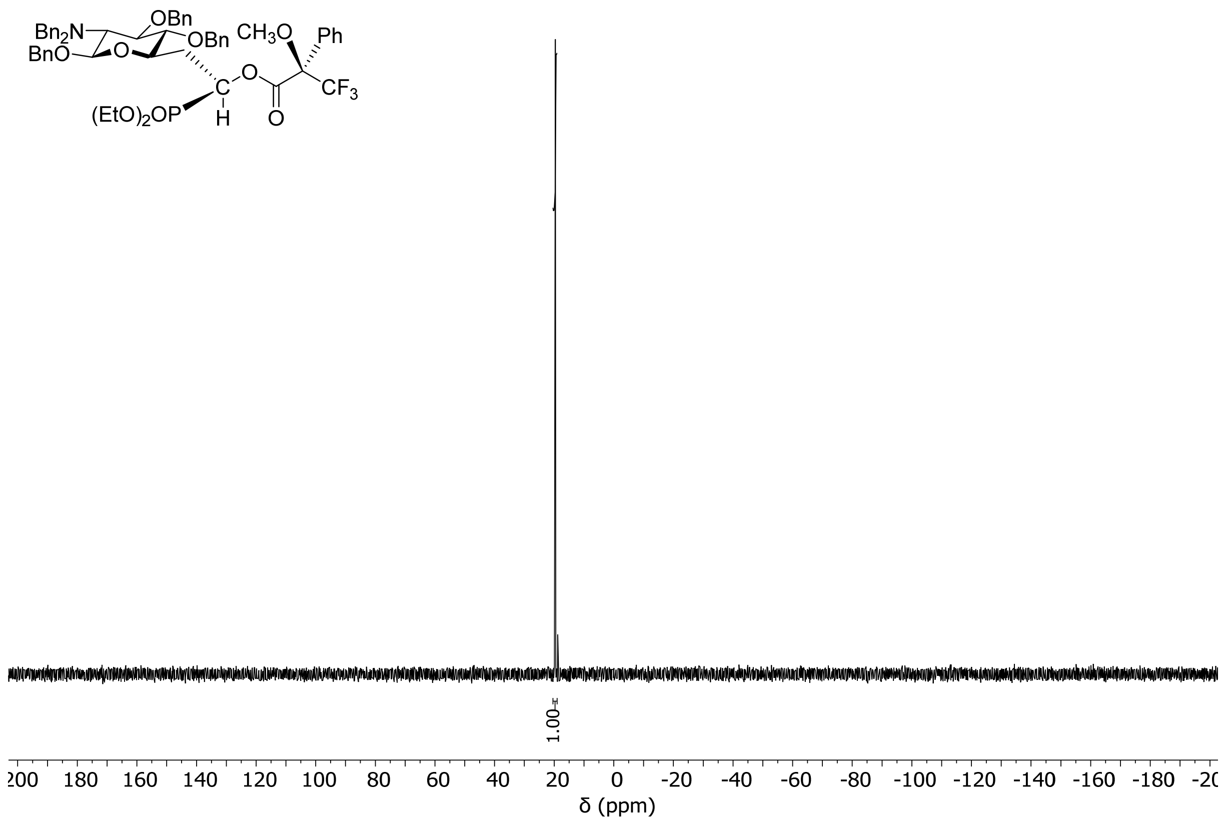

^{31}P NMR spectrum (202MHz, $CDCl_3$) of (*S*)-**8**-(*R*)-MTPA ester

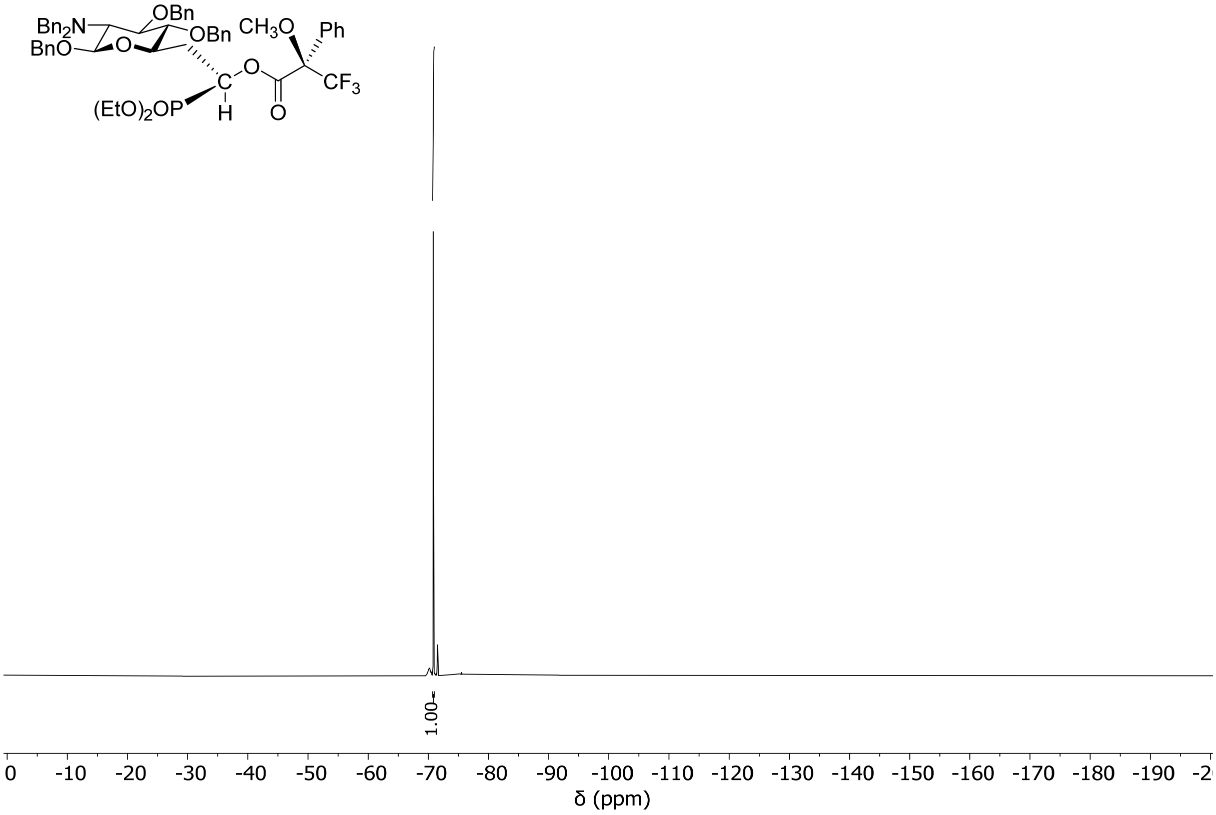

^{19}F NMR spectrum (471 MHz, $CDCl_3$) of (*S*)-**8**-(*R*)-MTPA ester

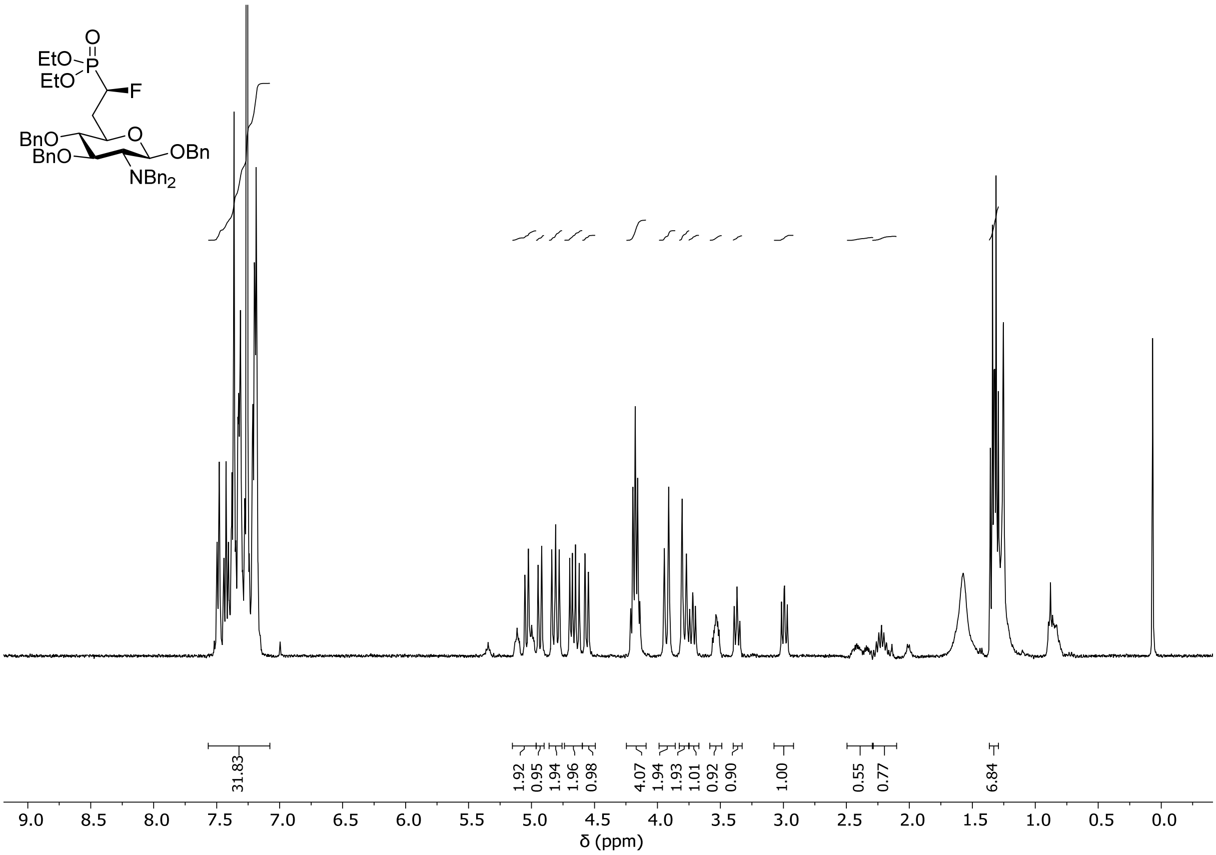

^{1}H NMR spectrum (400 MHz, $CDCl_3$) of (*R*)-**9**

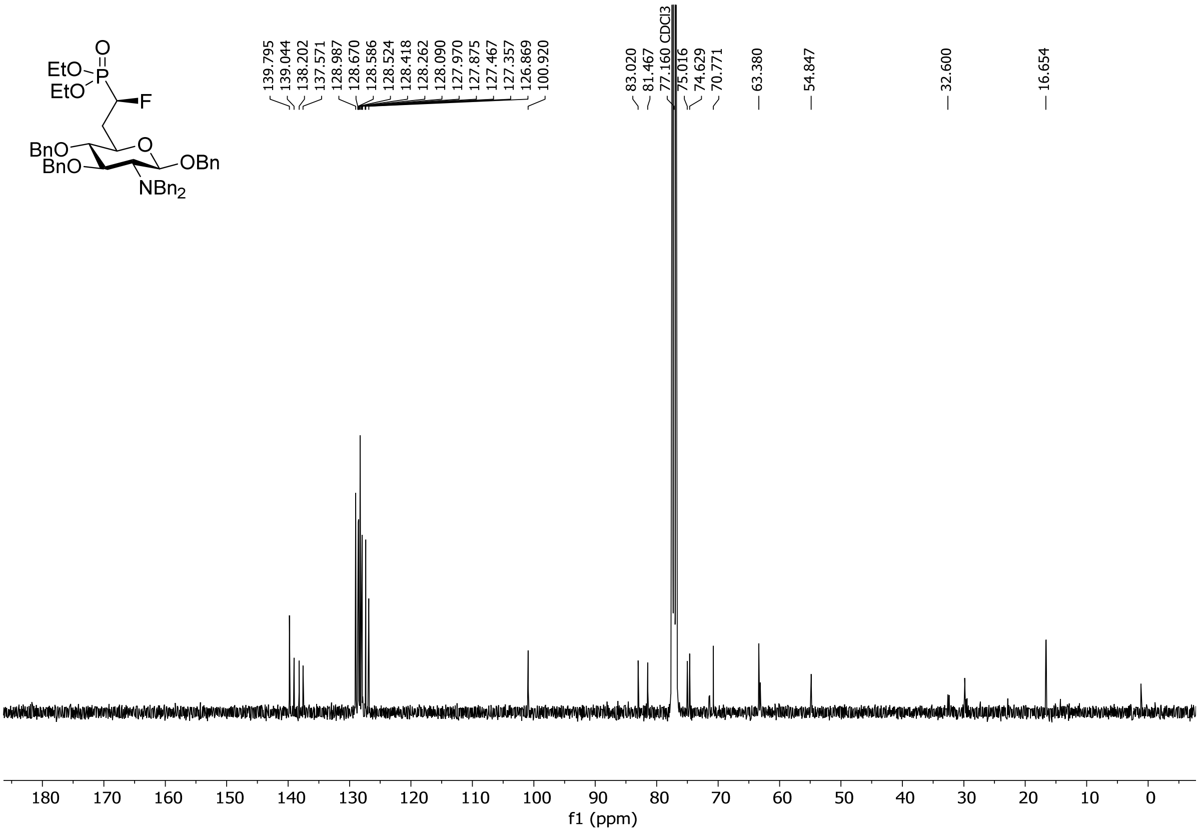

^{13}C NMR spectrum (101 MHz, $CDCl_3$) of (*R*)-**9**

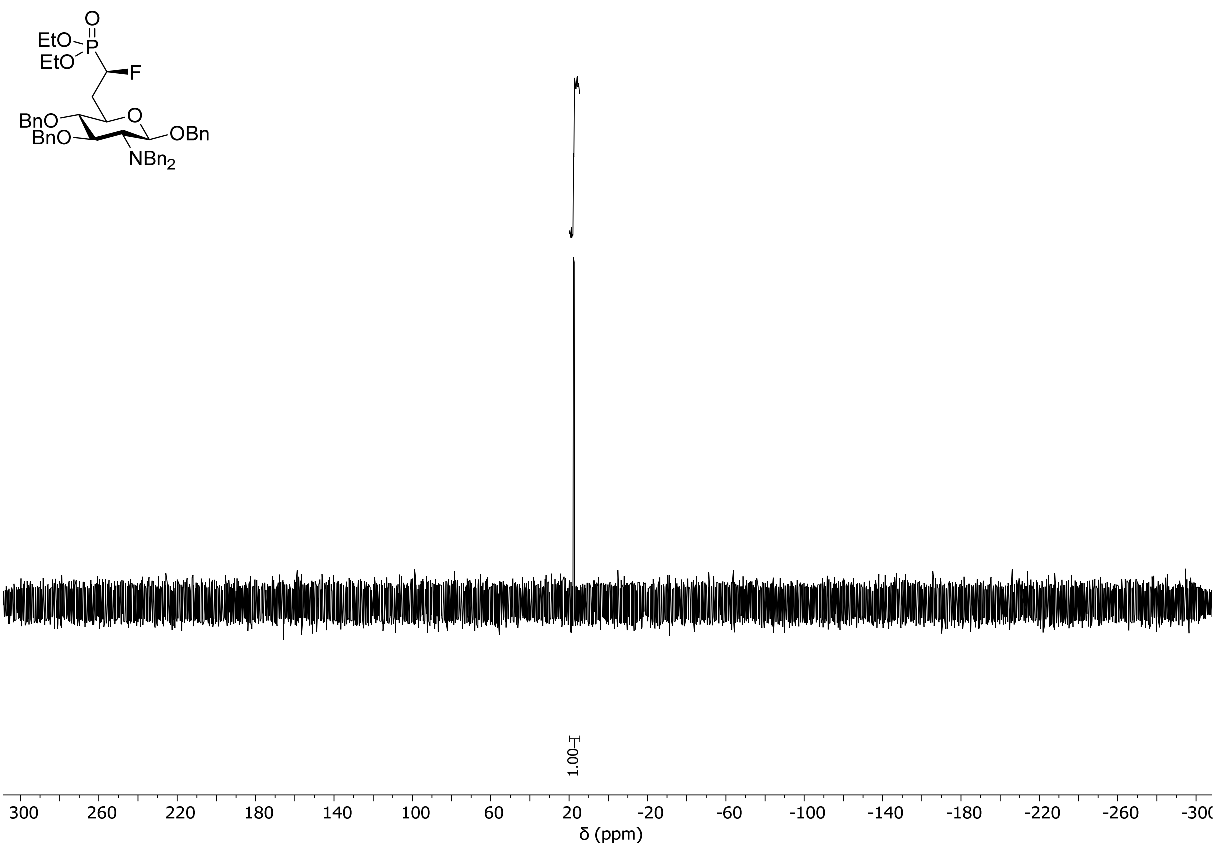

^{31}P NMR spectrum (162 MHz, $CDCl_3$) of (*R*)-**9**

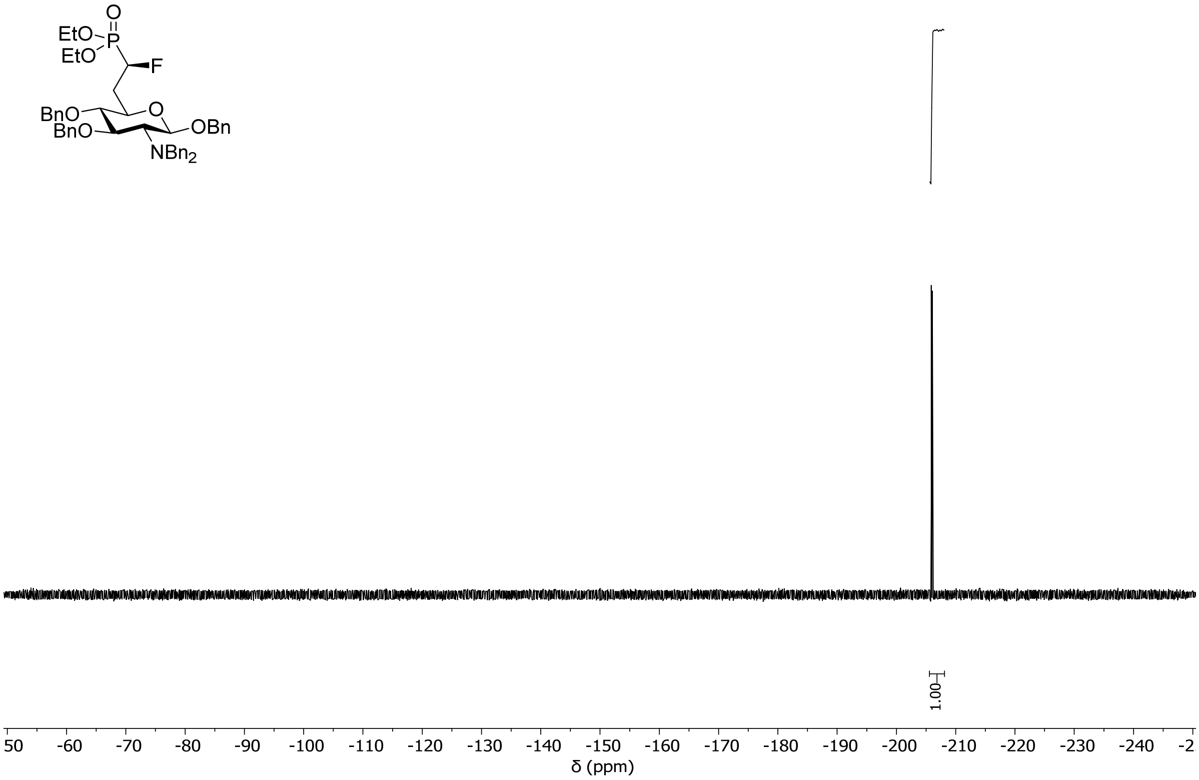

^{19}F NMR spectrum (377MHz, $CDCl_3$) of (*R*)-**9**

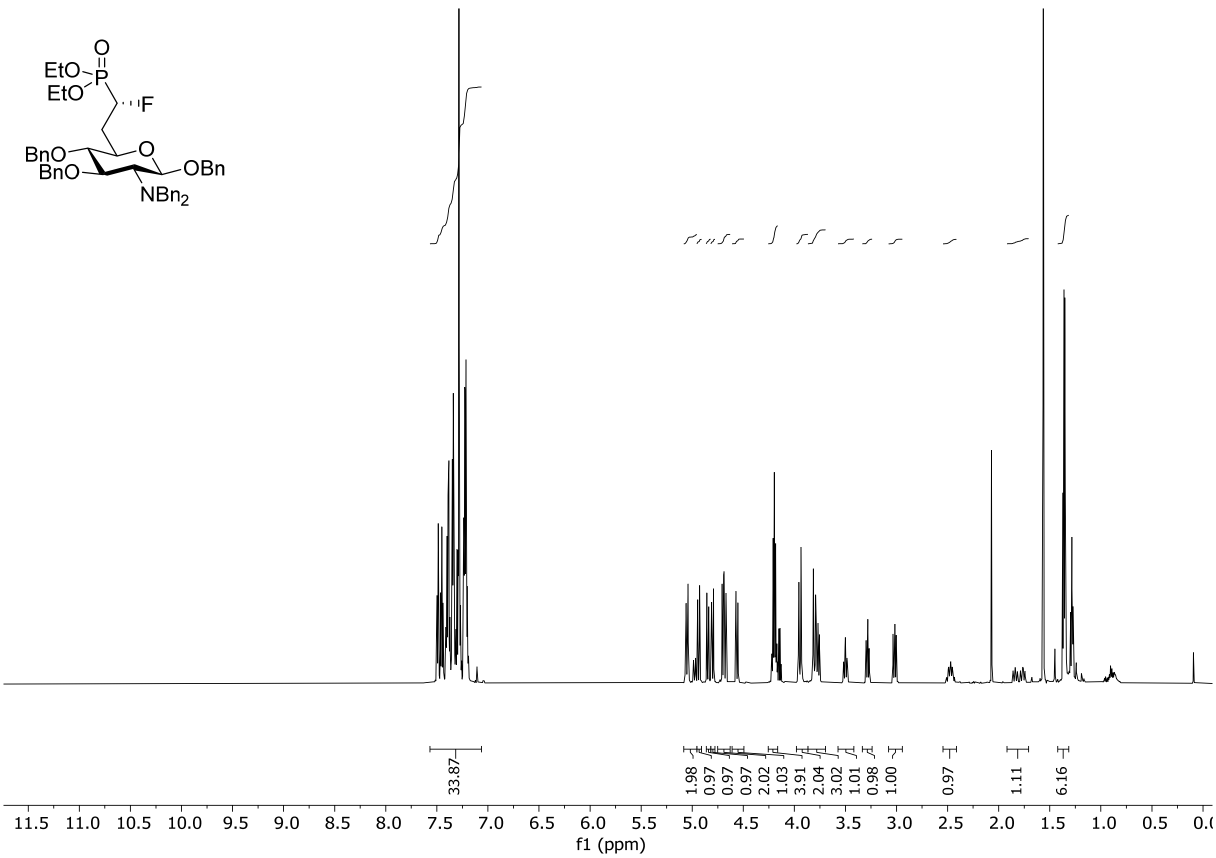

^{1}H NMR spectrum (600 MHz, CDCl$_3$) of (*S*)-**9**

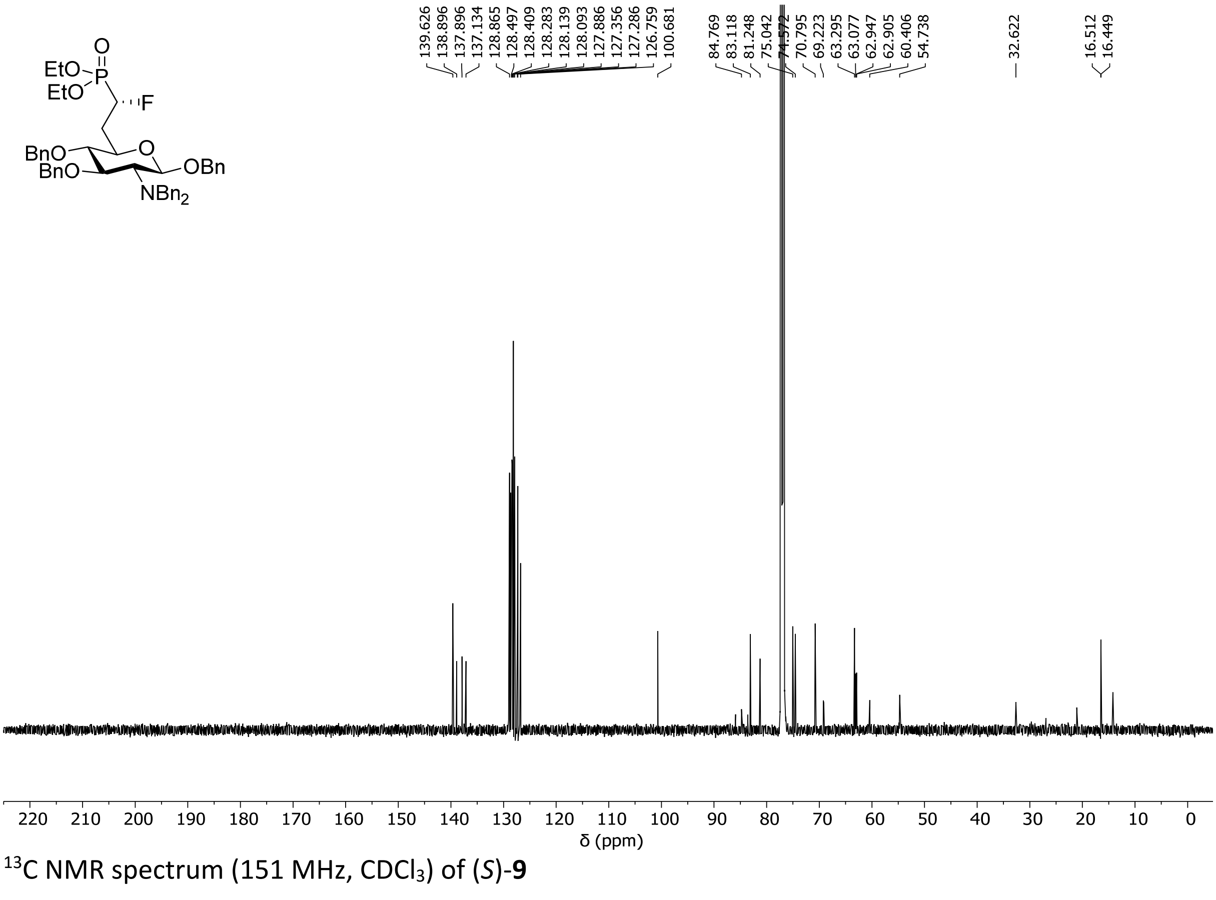

^{13}C NMR spectrum (151 MHz, CDCl$_3$) of (*S*)-**9**

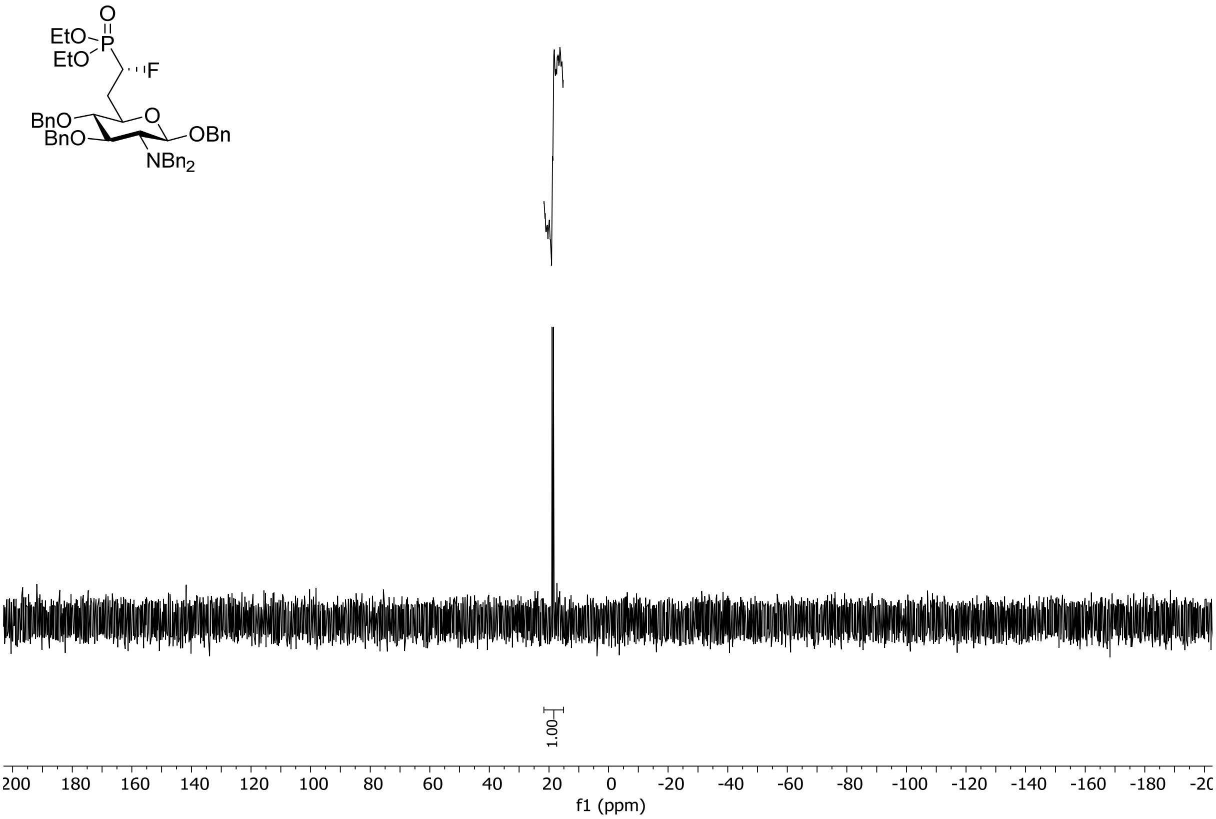

^{31}P NMR spectrum (162 MHz, $CDCl_3$) of (*S*)-**9**

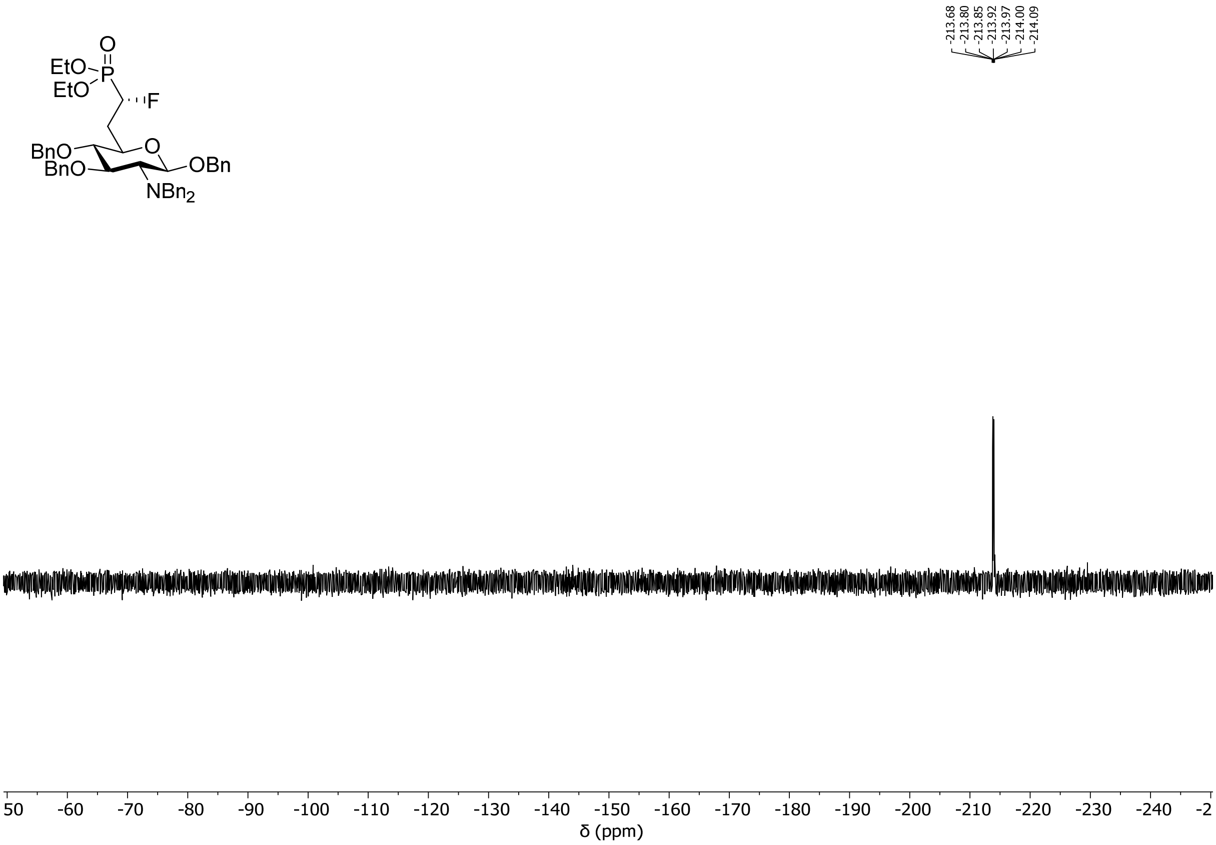

^{19}F NMR spectrum (377MHz, $CDCl_3$) of (*S*)-**9**

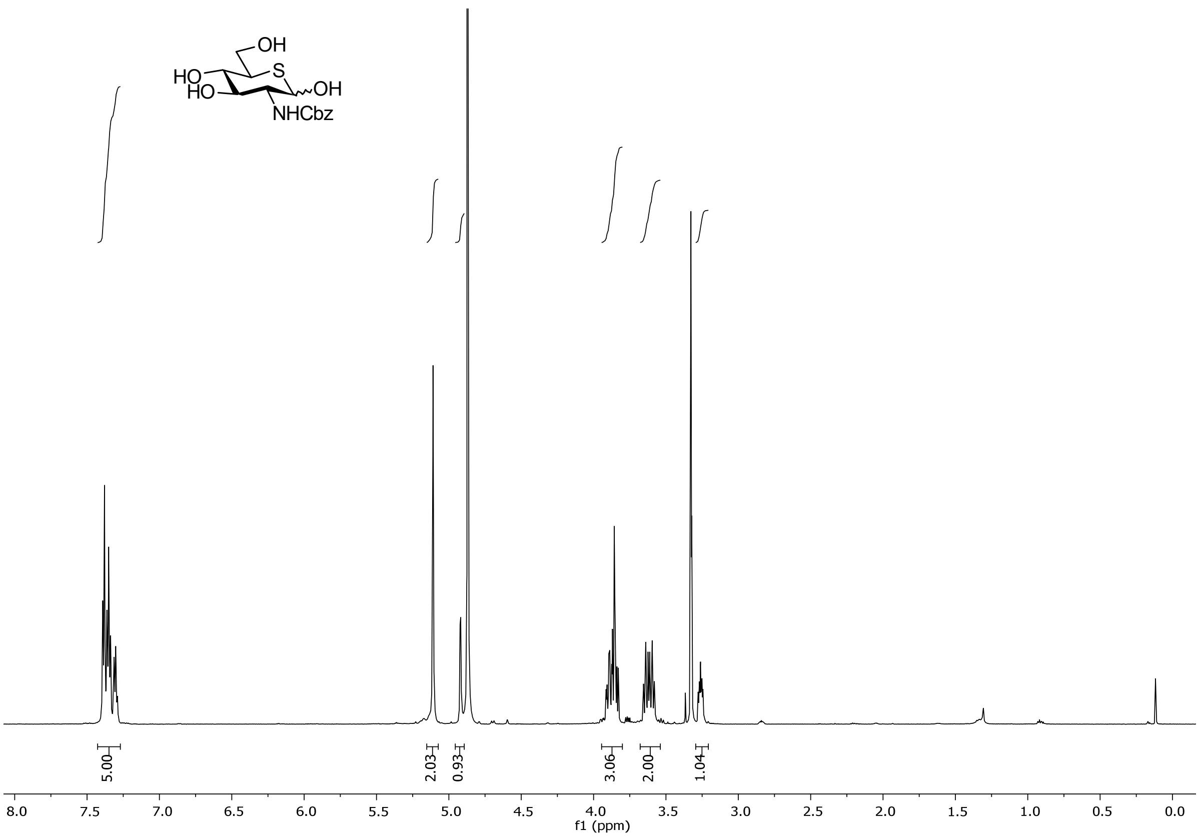

^{1}H NMR spectrum (600 MHz, MeOD) of **12**

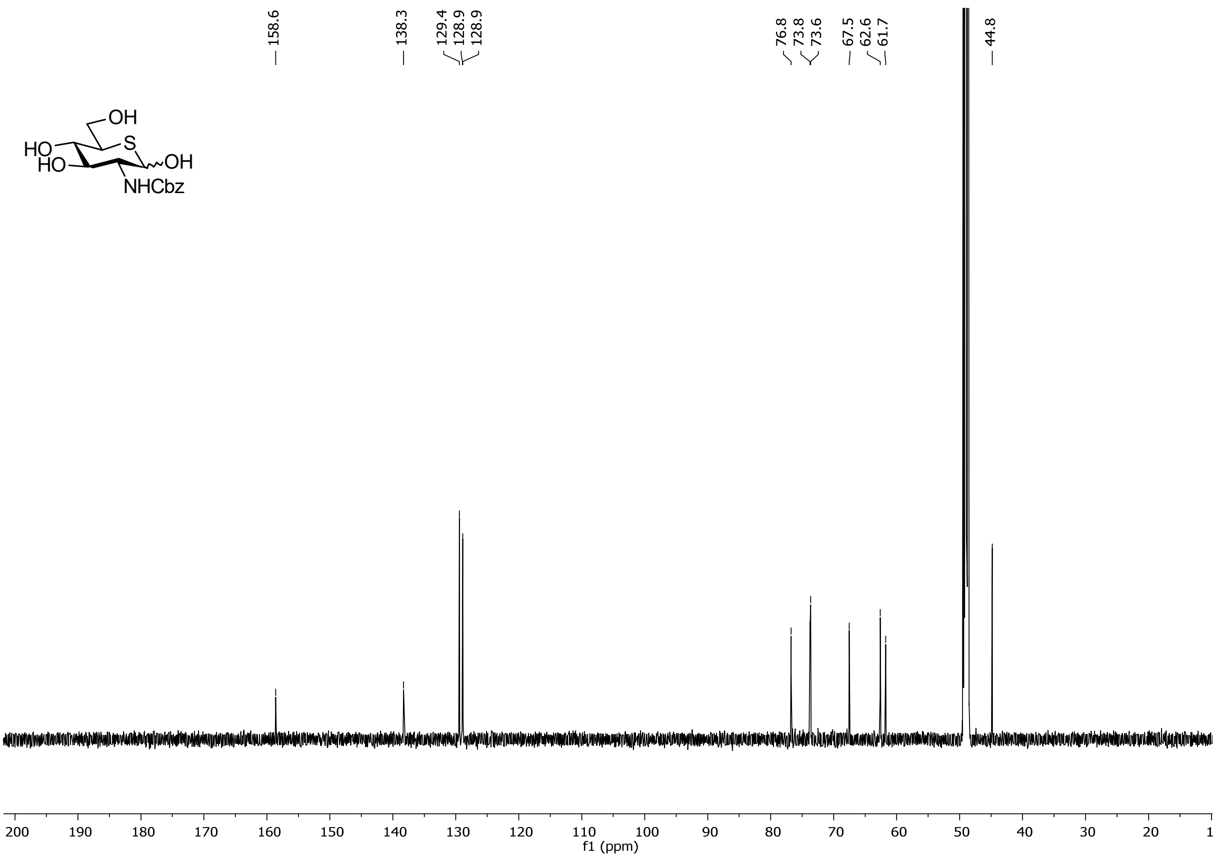

^{13}C NMR spectrum (151 MHz, D_2O) of **12**

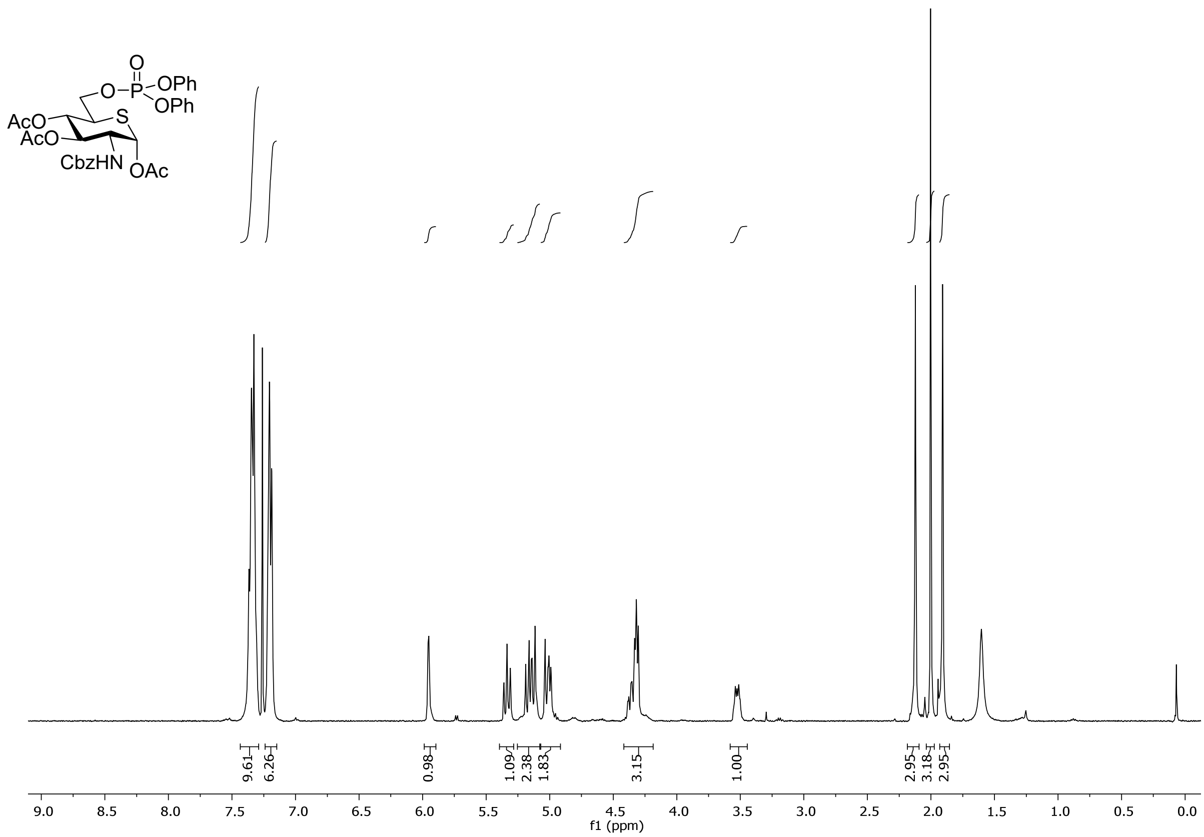

^{1}H NMR spectrum (400 MHz, $CDCl_3$) of **13**

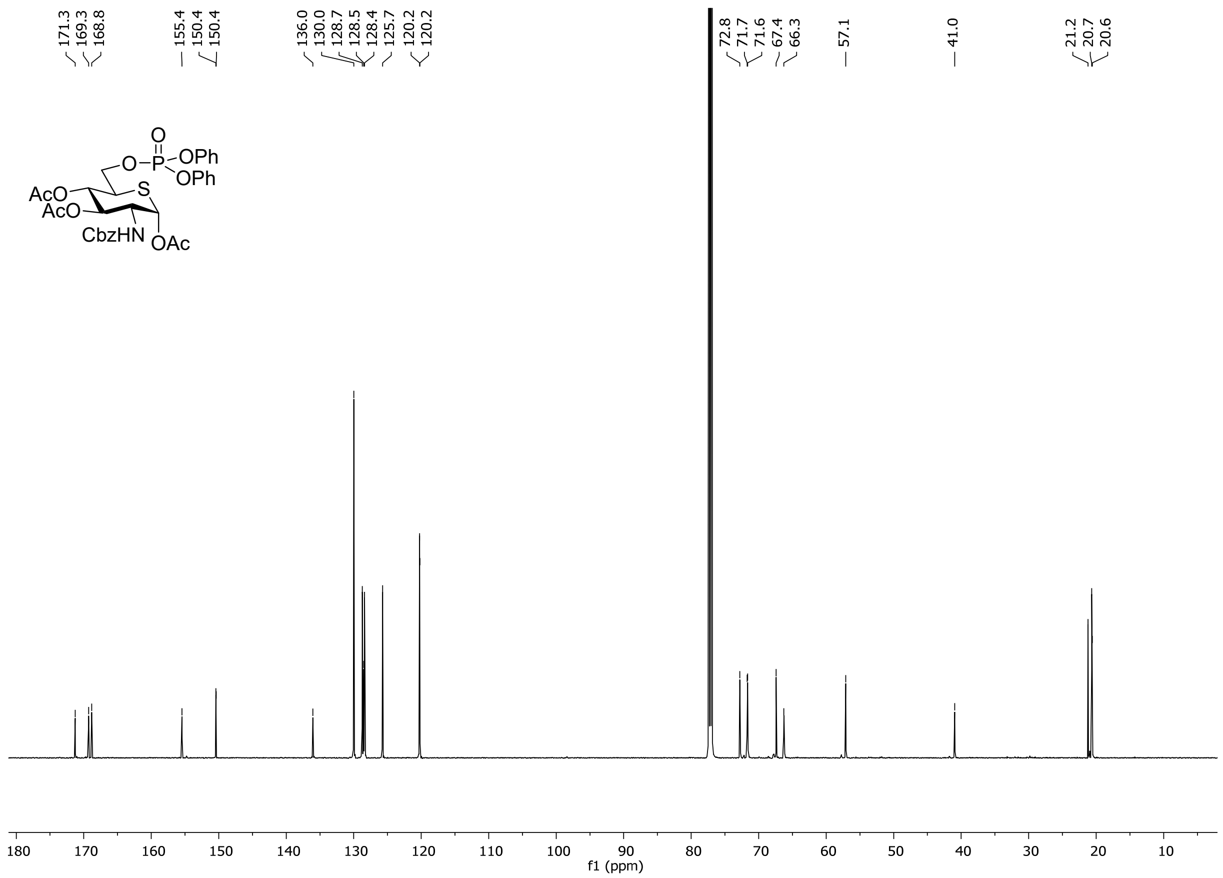

^{13}C NMR spectrum (151 MHz, $CDCl_3$) of **13**

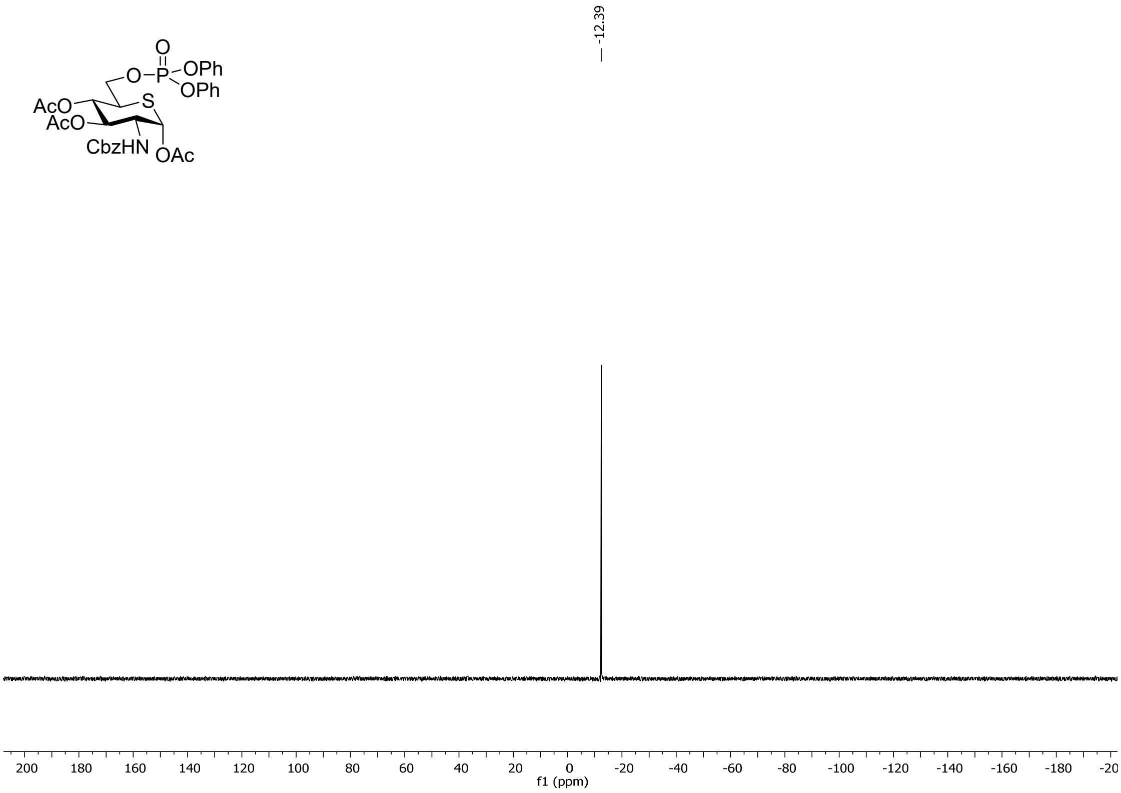

^{31}P NMR spectrum (162 MHz, $CDCl_3$) of **13**

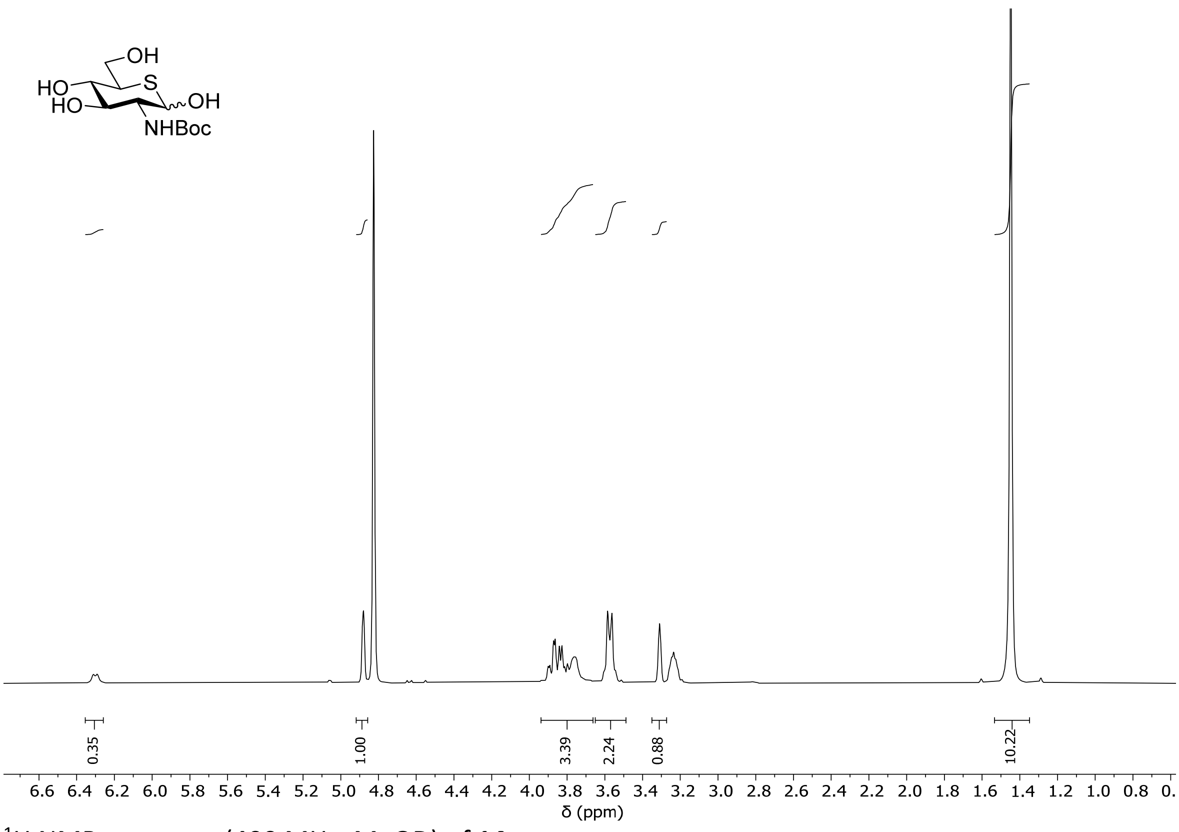

^{1}H NMR spectrum (400 MHz, MeOD) of **14**

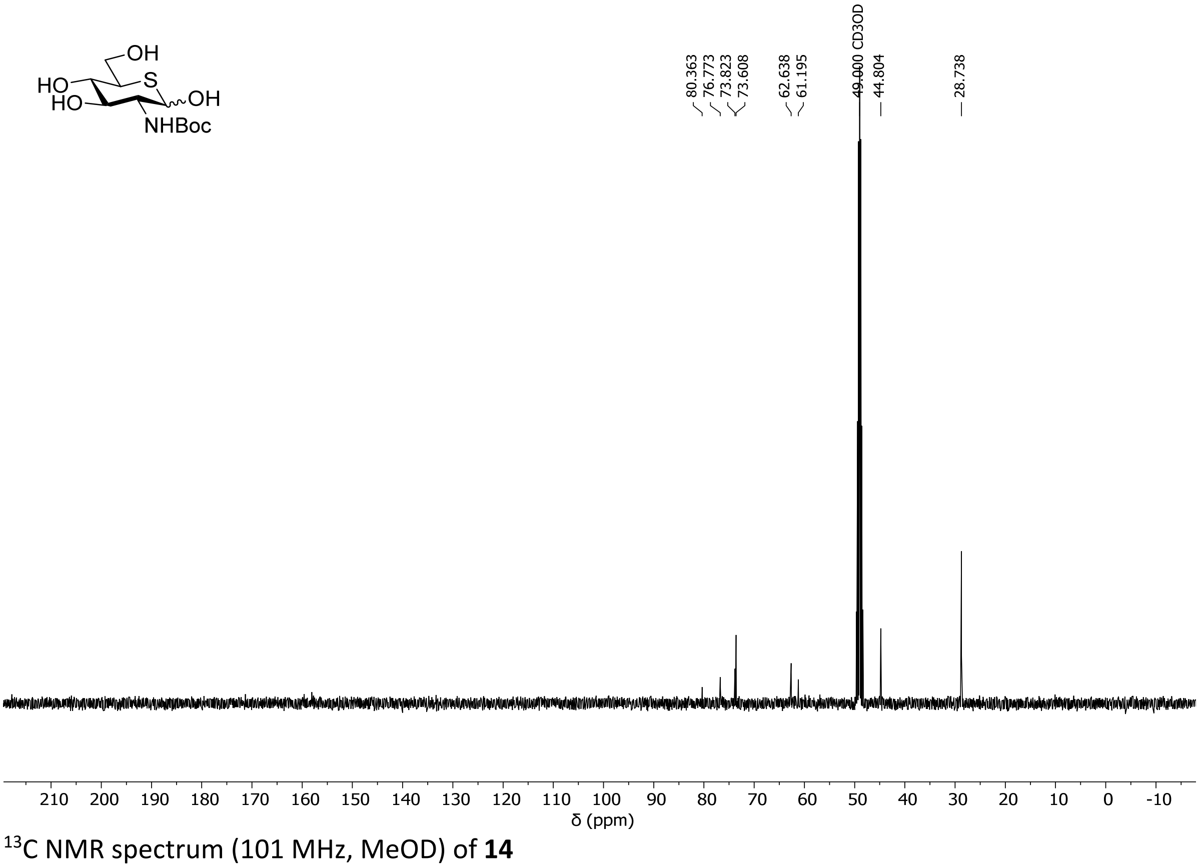

^{13}C NMR spectrum (101 MHz, MeOD) of **14**

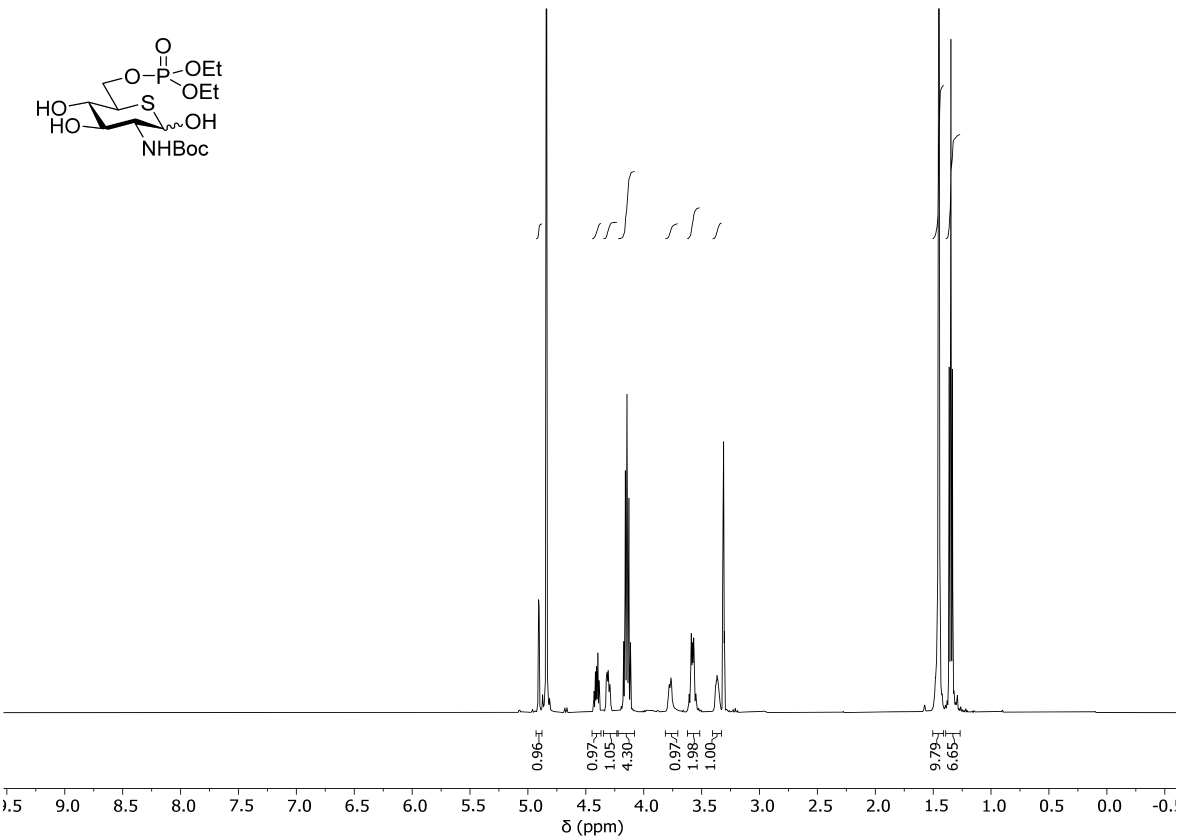

^{1}H NMR spectrum (500 MHz, MeOD) of **15**

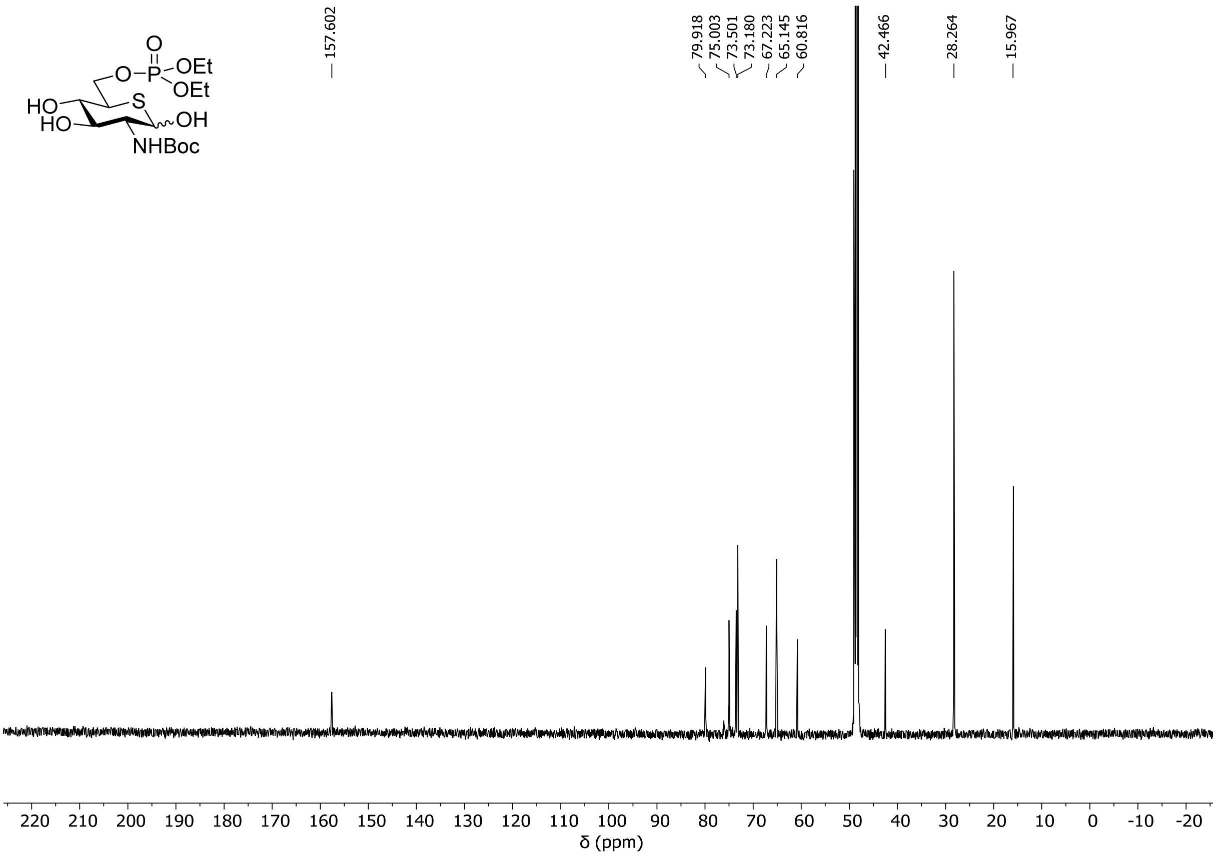

^{13}C NMR spectrum (126 MHz, MeOD) of **15**

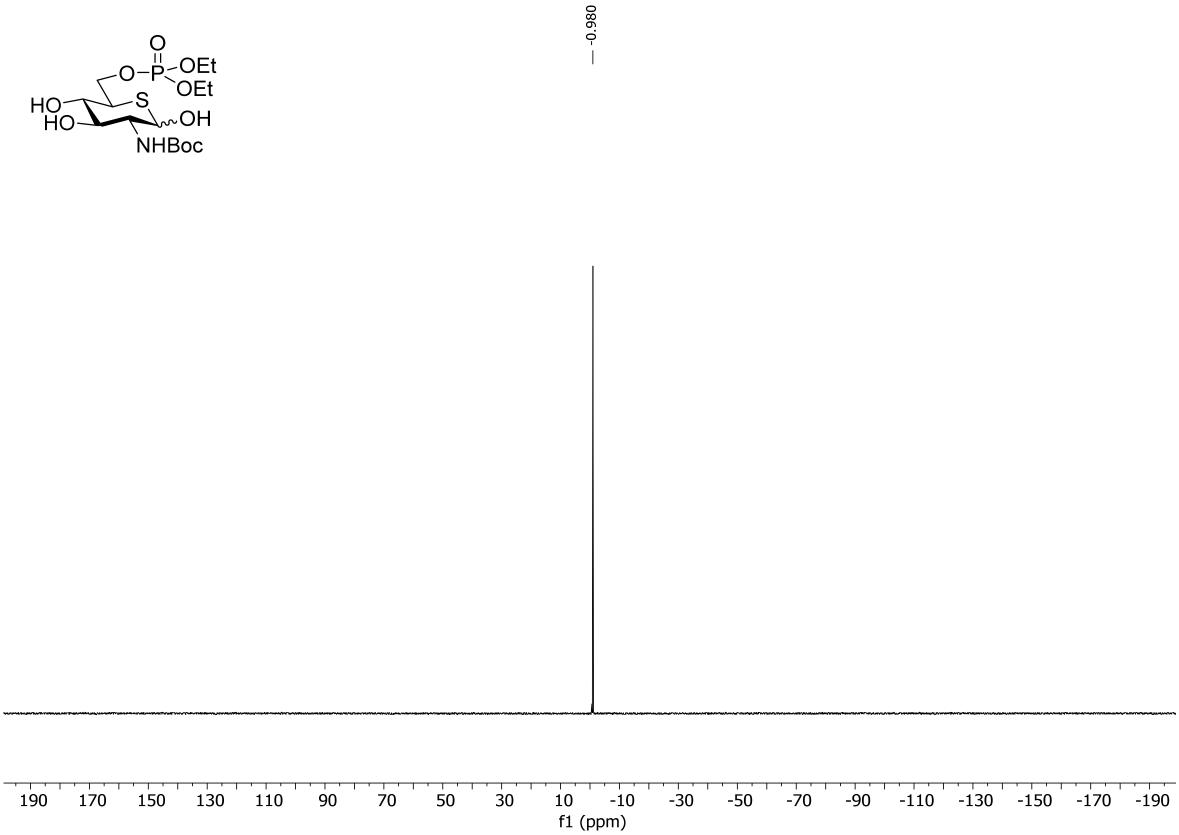

^{31}P NMR spectrum (202 MHz, MeOD) of **15**

8. References

1. Seco, J. M.; Quiñoá, E.; Riguera, R., The Assignment of Absolute Configuration by NMR. *Chem. Rev.* **2004,** *104* (1), 17-118, DOI: 10.1021/cr000665j.
2. Dale, J. A.; Mosher, H. S., Nuclear magnetic resonance enantiomer regents. Configurational correlations via nuclear magnetic resonance chemical shifts of diastereomeric mandelate, O-methylmandelate, and α-methoxy-α-trifluoromethylphenylacetate (MTPA) esters. *J. Am. Chem. Soc.* **1973,** *95* (2), 512-519, DOI: 10.1021/ja00783a034.
3. Swartz, M. A.; Tubergen, P. J.; Tatko, C. D.; Baker, R. A., Experimental Determination of pK_a Values and Metal Binding for Biomolecular Compounds Using ^{31}P NMR Spectroscopy. *J. Chem. Educ.* **2018,** *95* (1), 182-185, DOI: 10.1021/acs.jchemed.7b00508.
4. Wang, G. N.; Lau, P. S.; Li, Y. F.; Ye, X. S., Synthesis and evaluation of glucosamine-6-phosphate analogues as activators of glmS riboswitch. *Tetrahedron* **2012,** *68* (46), 9405-9412, DOI: 10.1016/j.tet.2012.09.015.
